# Functional Profiling of a *Plasmodium* Genome Reveals an Abundance of Essential Genes

**DOI:** 10.1016/j.cell.2017.06.030

**Published:** 2017-07-13

**Authors:** Ellen Bushell, Ana Rita Gomes, Theo Sanderson, Burcu Anar, Gareth Girling, Colin Herd, Tom Metcalf, Katarzyna Modrzynska, Frank Schwach, Rowena E. Martin, Michael W. Mather, Geoffrey I. McFadden, Leopold Parts, Gavin G. Rutledge, Akhil B. Vaidya, Kai Wengelnik, Julian C. Rayner, Oliver Billker

**Affiliations:** 1 Wellcome Trust Sanger Institute, Wellcome Genome Campus, Hinxton, Cambridgeshire, UK; 2 Research School of Biology, Australian National University, Canberra, Australia; 3 Drexel University College of Medicine, Philadelphia, PA, USA; 4 School of Biosciences, University of Melbourne, Royal Parade, Parkville, Australia; 5 DIMNP, CNRS, INSERM, University Montpellier, Montpellier, France

**Keywords:** carcinoma-associated fibroblast (CAF), tumor microenvironment, triple negative breast cancer, interferon, lncRNA, cancer inflammation, cancer immunology, myeloid cells, dendritic cells, radiation resistance

## Abstract

The genomes of malaria parasites contain many genes of unknown function. To assist drug development through the identification of essential genes and pathways, we have measured competitive growth rates in mice of 2,578 barcoded *Plasmodium berghei* knockout mutants, representing >50% of the genome, and created a phenotype database. At a single stage of its complex life cycle, *P. berghei* requires two-thirds of genes for optimal growth, the highest proportion reported from any organism and a probable consequence of functional optimization necessitated by genomic reductions during the evolution of parasitism. In contrast, extreme functional redundancy has evolved among expanded gene families operating at the parasite-host interface. The level of genetic redundancy in a single-celled organism may thus reflect the degree of environmental variation it experiences. In the case of *Plasmodium* parasites, this helps rationalize both the relative successes of drugs and the greater difficulty of making an effective vaccine.

## Highlights

• Two-thirds of *Plasmodium berghei* genes contribute to normal blood stage growth
• The core genome of malaria parasites is highly optimized for rapid host colonization
• Essential parasite genes and pathways are identified for drug target prioritization
• Low functional redundancy reflects the constant environment encountered by a parasite

## Graphical Abstract

**Figure:**
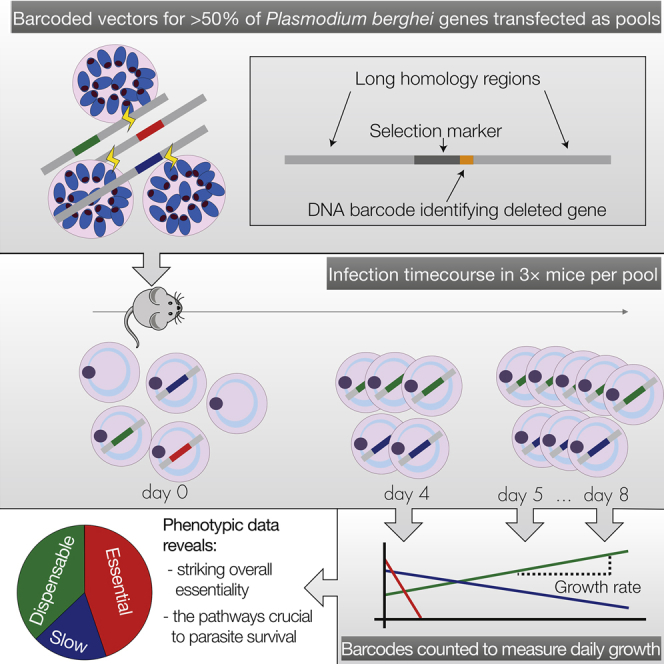

## Teaser

An in vivo genetic screen in a mouse model of malaria reveals the essential genes and pathways required by *Plasmodium* parasite, with a surprising two-thirds of the genome being required for normal parasite growth in the blood.

## Introduction

Throughout the tree of life, the evolution of parasitism has been accompanied by drastic reductions in genome size and gene numbers (Jackson et al., 2016, Vivarès et al., 2002, Wolf and Koonin, 2013). Prominent examples are found in the phylum Apicomplexa, one of the most successful taxa of parasitic protozoa. The Apicomplexa include important parasites of livestock and human pathogens, such as *Toxoplasma gondii*, which infects approximately one-third of the human population and causes pathology in immunodeficient individuals and malaria parasites (genus *Plasmodium*), among which *P. falciparum* remains the biggest killer of all parasites, leading to an estimated 214 million clinical cases and 438,000 deaths annually (WHO, 2015).

The evolution of Apicomplexa from a free-living, photosynthetic ancestor with a red algal plastid and a broad repertoire of genes was accompanied by drastic gene losses (Woo et al., 2015). However, genomic reduction is not limited to parasites and may be the predominant mode of genome evolution overall (Wolf and Koonin, 2013). The consequences of shrinking genomes on the importance of the individual genes that are retained has never been explored systematically. In free-living organisms such as *Escherichia coli*, *Saccharomyces cerevisiae*, *Drosophila melanogaster*, or *Caenorhabditis elegans*, the majority of genes have no loss-of-function phenotypes when tested under laboratory conditions (Dietzl et al., 2007, Gerdes et al., 2003, Giaever et al., 2002, Kamath et al., 2003, Winzeler et al., 1999), even though many genes are highly conserved in evolution and must therefore fulfil important functions. In *S. cerevisiae*, many loss-of-function phenotypes were only revealed through pairwise gene disruptions, leading to the notion of genetic buffering, i.e., that networks of functionally overlapping genes provide protection against mutation (Boone et al., 2007, Costanzo et al., 2010). Functions for almost all genes were found by varying the conditions under which growth assays were conducted, supporting an alternative concept that most genes in single-celled eukaryotes contribute to survival only in specific environments (Hillenmeyer et al., 2008).

Whether these same models hold true in the reduced genomes of parasites is unknown, and there are plausible counter arguments. On the one hand, parasites have outsourced important metabolic functions to the host, and the smaller range of challenges faced in an intracellular environment might predict that each gene retained by the parasite should, on average, contribute more to survival. On the other hand, for malaria parasites to gain access to the relatively predictable environment inside a red blood cell, they need a complex life cycle to travel between hosts. This involves transmission through a blood-sucking mosquito vector in which the parasite reproduces sexually and lives extracellularly, including being exposed directly to the immune system of the vector. In the mosquito, and during the obligatory intracellular liver stage that precedes colonization of the blood, malaria parasites experience very different environments, each requiring genes to fulfil stage-specific functions (Aly et al., 2009). These factors could clearly lead to a gain in genome complexity and a consequent increase in overall redundancy.

Understanding the pressures that influence gene function in parasites is of more than theoretical importance. The repeated and rapid evolution of antimalarial drug resistance remains the largest single challenge to malaria control, with resistance to artemisinin-combination therapies, the current front-line treatment, now well established and spreading in Southeast Asia (Saunders and Lon, 2016). The identification of drug targets is therefore a continual and urgent need. Knowing the genes and biochemical pathways that contribute to parasite growth during the asexual blood stages, which cause all the symptoms and pathology of malaria, will be critical to guide the discovery and prioritization of targets for curative drugs. Furthermore, the chance of finding compounds that act on multiple life-cycle stages, a key priority for antimalarial drug development (Burrows et al., 2017), will be directly related to the number of essential genes with stage-transcending functions.

*Plasmodium* species possess compact genomes of 18–30 Mb, which encode 4,600–4,700 core protein-coding genes, along with a varying number of largely subtelomeric multigene families, distributed across 14 chromosomes (e.g., Otto et al., 2014). No genome scale functional screen has been carried out in any *Plasmodium* species, which are hard to transfect, have low homologous recombination rates, and whose genomes are so AT-rich and repetitive that manipulating their DNA in *E. coli* is challenging. Recent innovations in the genetic system of *P. berghei*, a malaria parasite infecting rodents, have overcome major roadblocks for large-scale reverse genetic screens in this species. Recombinase-mediated engineering of AT-rich DNA (Pfander et al., 2011) has enabled the creation of the *Plasmodium* genetic modification community resource, *Plasmo*GEM, which contains knockout vectors that integrate efficiently and carry gene-specific molecular barcodes (Schwach et al., 2015). Here, we use this resource to measure growth rate phenotypes in mice for 2,578 *P. berghei* genes, representing more than half of its protein-coding genome. We estimate that almost two-thirds of parasite genes contribute to normal asexual growth of the blood stage in vivo, an unexpectedly large number for a single phase of a complex life cycle, which we propose is a consequence of genomic reduction during the evolution of parasitism.

## Results

### Determining Growth Rate Phenotypes from Barcode Counts

**Figure 1:**
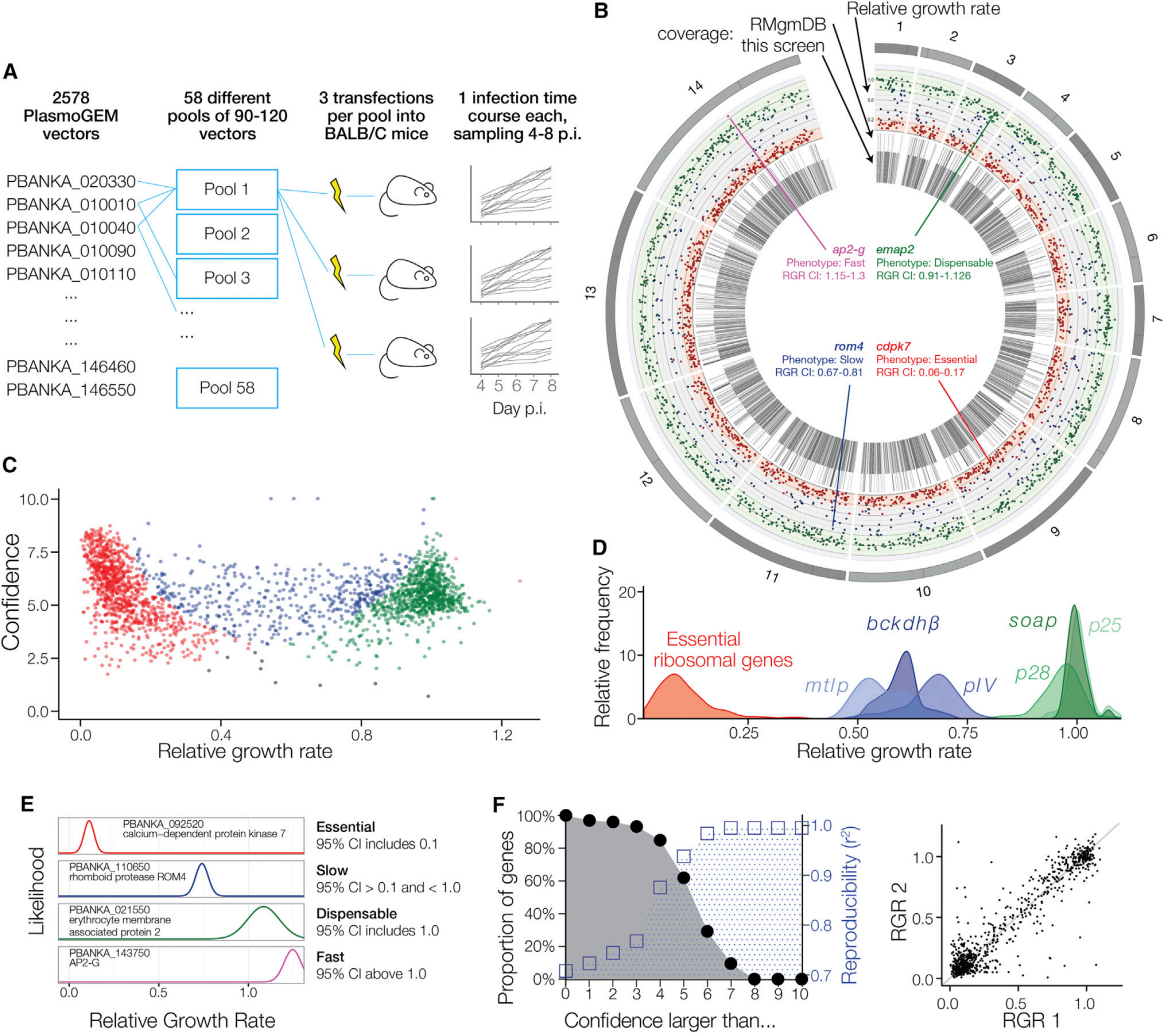
A BarSeq Screen for Parasite Growth Rate (A) Schematic illustration of screen design. (B) Circos plot showing, from the outer to inner circle, chromosome, mutant RGR (color coded by phenotype as in E), extent of previous phenotyping as reported on RMgmDB, and coverage in our screen. (C) Screenshot of the *Plasmo*GEM phenotype viewer showing RGR measurements plotted against confidence expressed as the negative logarithm of their variance. See (E) for color coding of phenotypes. (D) Frequency distribution of the >50 RGR replicates available for six control genes compared to essential ribosomal genes. (E) Asexual growth phenotypes defined using confidence intervals (CI) and illustrated with representative genes. (F) Graph on the left showing percentage of genes with at least one RGR measurement at a given confidence limit (black circles) and how confidence relates to experimental reproducibility (blue squares). Reproducibility of independent duplicates is illustrated by the regression plot on the right for confidence 4 or above, a level reached by at least one measurement for 85% of genes in the screen. See also Figure S1 and Table S1.

In the absence of a continuous culture system for asexual blood stages, creating arrayed libraries of knockout clones would be impractical in *P. berghei.* To increase scale, we therefore generated pools of mutants by simultaneously co-transfecting multiple barcoded vectors. The optimal pool size was determined by the overall transfection efficiency and by the variance in integration rates between individual vectors. The library was screened in 58 pools of ∼100 barcoded vectors in inbred mice, as illustrated in Figure 1A.

Following elimination of wild-type parasites by drug selection, the relative growth rate (RGR) for each mutant was determined by counting barcodes in daily blood samples on a next-generation sequencer (Gomes et al., 2015). Changes in barcode abundance were normalized against seven control vectors each pool. Some mutants increased over time, while others dropped out on different days depending on their competitive fitness and their initial abundance. We therefore aggregated data from at least three replicate transfections into a single best estimate of RGR and calculated a 95% confidence interval (CI) for each mutant. To validate the initial screen the majority of vectors (69.6%) were retransfected in at least one additional pool. These validation pools were configured with different compositions to assess the impact of pool composition on phenotype, with the most abundant mutants from the first rounds excluded from rescreening to make rarer mutants more readily detectable.

RGR measurements were generated for 2,578 genes (Table S1), increasing more than 5-fold the number of *P. berghei* genes for which phenotype data had previously been recorded (RMgmDB) (Khan et al., 2013). The screen covered all 14 chromosomes evenly (Figure 1B), with the exception of the subtelomeric regions, as discussed below. In Figure 1C, growth measurements for all mutants are plotted against a measure of confidence, which we derive from a model of the variance underlying the barcode counts. The observed variance between independent measurements of the control genes was low (Figure 1D), showing RGR values to be robust. Four growth phenotypes could be distinguished: essential genes, slow growing mutants, dispensable genes, and fast growers. These are color-coded in Figure 1C and defined and illustrated with examples in Figure 1E. A phenotype database was built to browse, search, download, and analyze RGR measurements, which also offers access to the raw daily barcode ratios from individual transfections (http://plasmogem.sanger.ac.uk). The computed confidence score for RGR in one pool of vectors was a strong predictor of reproducibility for the same gene tested in a second validation pool, showing phenotypes could be measured reliably in a way that was largely independent of pool composition and validating both the re-screening and analysis strategy (Figure 1F).

### Most *P. berghei* Genes Are Required for Normal Growth of Asexual Blood Stages In Vivo

**Figure 2:**
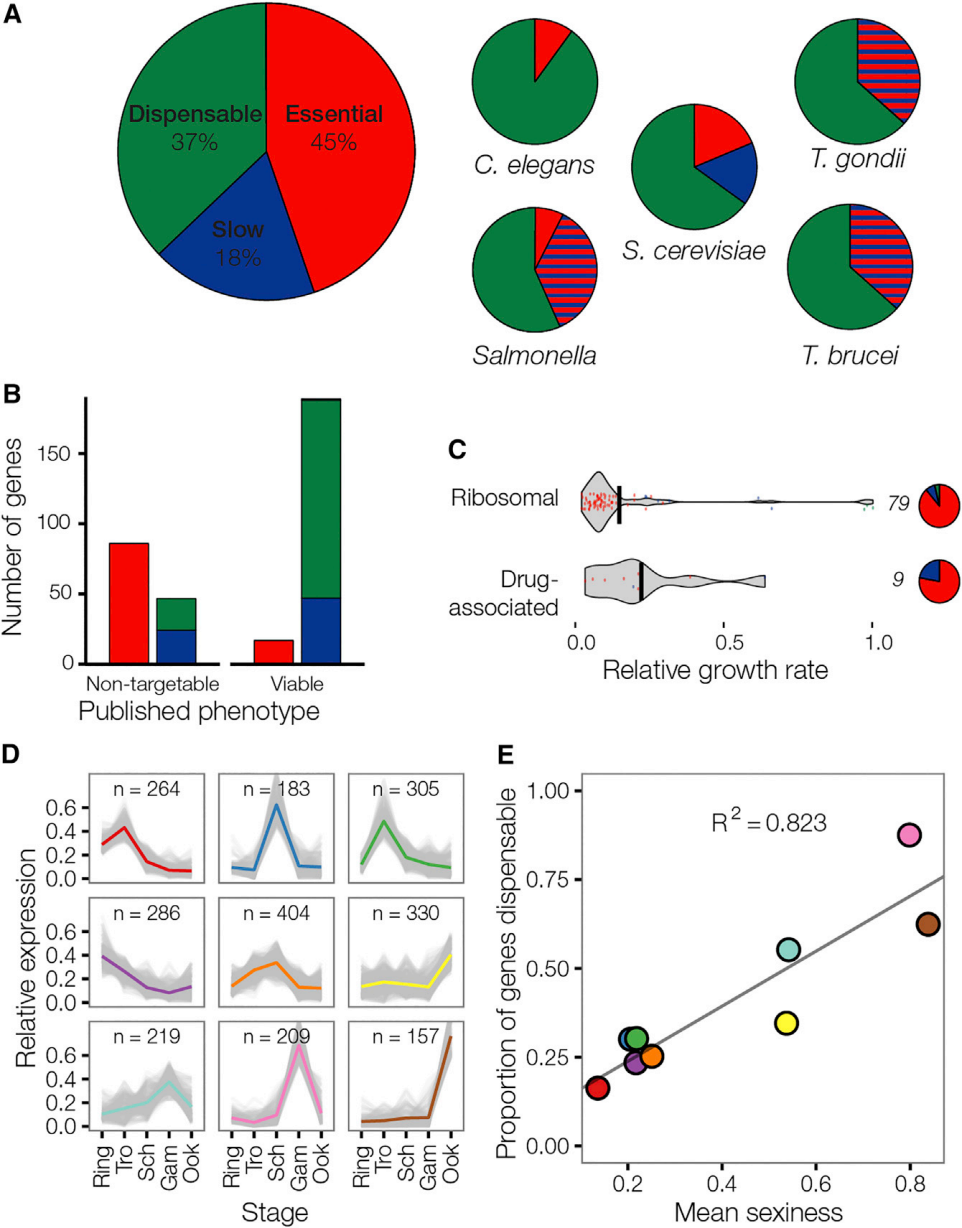
Most *P. berghei* Genes Are Required for Normal Growth of Asexual Blood Stages In Vivo (A) Frequency distribution of phenotypes in *P. berghei* compared to published data from other eukaryotes. Data from yeast are for growth in rich medium. Data from *T. gondii* are from a CRISPR-Cas9 screen in human foreskin fibroblasts. Genes required for normal growth are hatched red/blue since lethality and reduced growth phenotypes were not distinguished. *Salmonella* Typhimurium genes are for normal growth in medium (red) or additionally required for oral colonization of farm animals (hatched). *T. brucei* data is from an RNAi in vitro screen. See text for data sources. (B) BarSeq phenotypes compared to published data from RMgmDB. (C) Average RGR and phenotype distribution for predicted essential genes. Gene numbers per category shown next to pie charts. Enrichment for essential genes is significant at p < 0.001 for ribosomal genes and p < 0.1 for known drug targets. (D) All expressed genes covered by the screen were grouped into nine clusters depending on their relative expression across five life-cycle stages. All genes within a cluster were weighted equally. (E) For each cluster, the proportion of normalized read counts from sexual (gametocyte and ookinete) versus asexual developmental stages was calculated (“sexiness”) and plotted against the proportion of dispensable genes in that cluster. Ring, ring stage parasites; Tro, trophozoites; Sch, schizonts; Gam, gametocytes; Ook, ookinetes. See also Figures S2 and S3 and Tables S3 and S4.

We found 44.9% of genes were essential, and a further 18.0% of mutants showed reduced growth (Figure 2A). Thus, 62.9% of genes were required for normal asexual growth of *P. berghei* in mice, an unexpectedly large proportion considering that genome-wide knockout screens in other organisms showed much lower levels of essentiality (Figure 2A; Table 1) (Alsford et al., 2011, Barquist et al., 2013, Giaever et al., 2002, Kamath et al., 2003, Sidik et al., 2016).

**Table 1:** Gene Essentiality and Genome Size of Selected Species

Species	Genome Size (Mb)	Protein Coding Genes	Genes Contributing to Normal Growth	%	Assay	Method	Reference
*S. cerevisiae*	12.1	5,916	1,105 essential	34.9	competitive growth in rich medium	barcoded mutants quantified on nucleotide arrays	Giaever et al., 2002, Deutschbauer et al., 2005
			962 slow				
*Trypanosoma brucei*	35.0	7,500	2,745	36.6	6-day extracellular blood stream forms in vitro	RNA interference	Alsford et al., 2011
*Toxoplasma gondii*	63.0	8,158	3,263^b^	40.0	intracellular tachyzoites in cultured fibroblasts	CRISPR gRNA screen	Sidik et al., 2016
*P. berghei*	18.5	4,616^a^	2,123^b^ essential	62.9^b^	relative asexual intraerythrocytic growth in vivo	BarSeq	this study
			803^b^ slow				

a Core genome according to Otto et al. (2014).

b Extrapolated from the covered genome.

**Figure S1:**
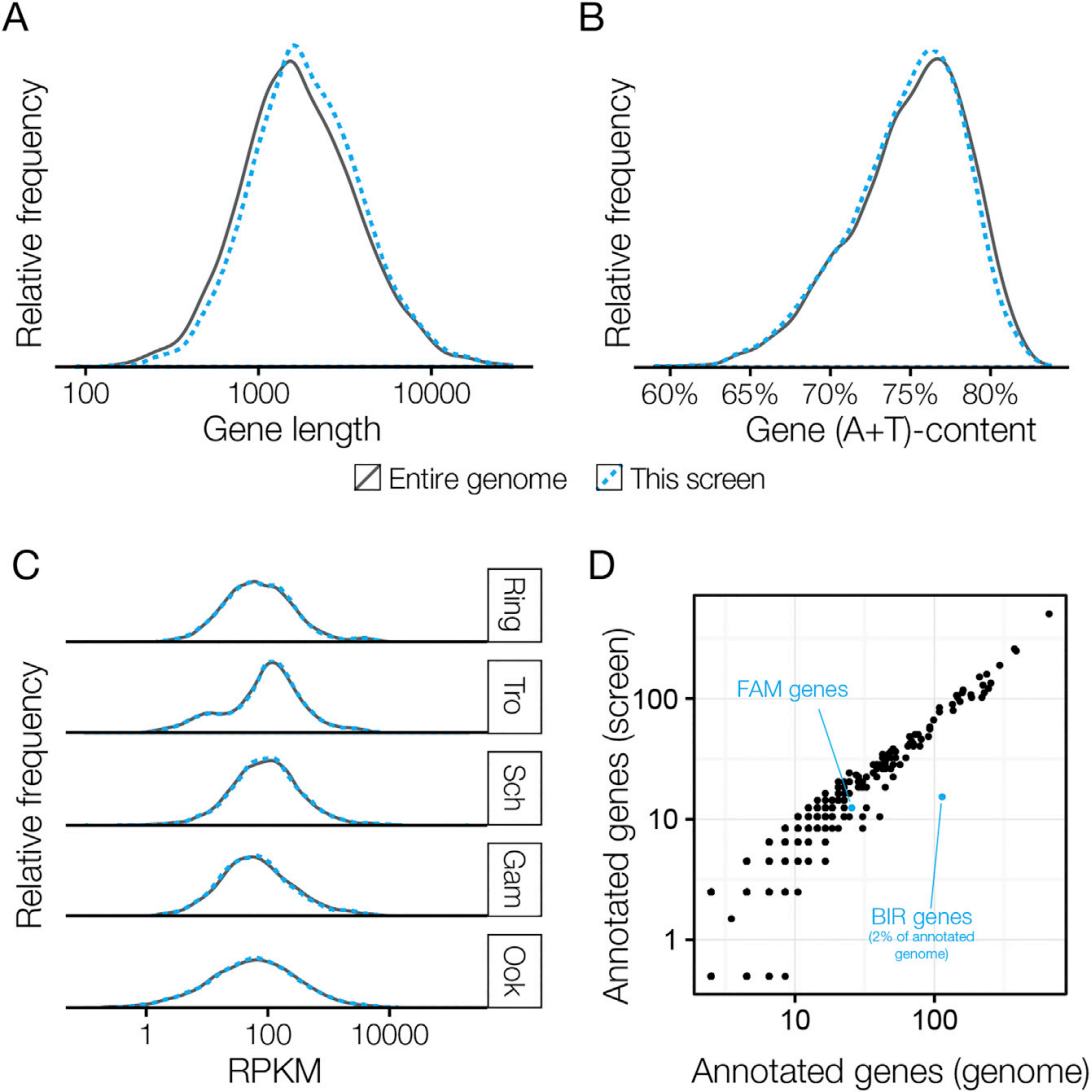
Genes in the Screen Are Largely Representative of the Genome, Related to Figure 1 and Table S2 (A and B) Genes for which targeting vectors could be generated were similar (A) in size and (B) in A+T nucleotide content. (C) Targeted genes had expression levels representative of the genome across all life stages (RPKM data from Otto et al., 2014). (D) Relative representation of GO terms and multigene families in the genome and and the screen. The screen was largely representative with the exception of the *P. berghei pir* family (*BIR*) which we suspect is underrepresented due to its subtelomeric genomic location and repetitive nature. However, the well represented FAM family is shown for comparison.

Genes in the screen were representative of the genome with respect to all parameters tested, including gene length, AT content, expression profiles, and functional annotation. The only exception were members of a large multigene family of *Plasmodium* interspersed repeat (*pir*) genes with roles in immune evasion, which were underrepresented in the vector resource, probably due to their repetitive DNA sequence (Figures S1A–S1D; Table S2). The *pir* family accounts for only 2.0% of *P. berghei* genes (Otto et al., 2014). The impact of the underrepresentation of *pir* genes on the unexpected phenotype distribution is therefore minimal, although we find most *pirs* unsurprisingly dispensable.

**Figure S2:**
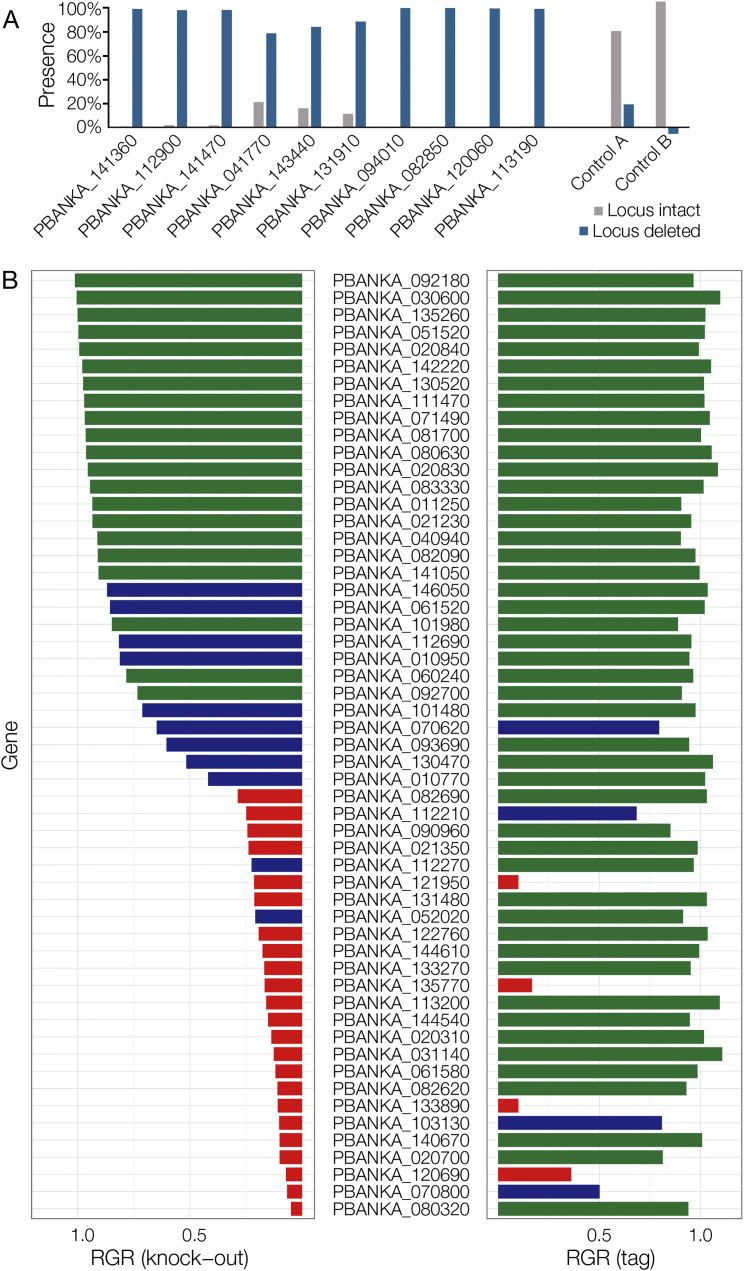
Validation of Dispensable and Essential Phenotypes, Related to Figure 2 (A) For 10 genes whose disruption had previously failed (RMgmDB), quantitative PCR was used to measure the proportion of parasites that retained the wild-type locus following transfection of individual deletion vectors. In each case the population was dominated by viable mutant parasites, validating the screen result. Controls A and B show a reciprocal primer swap for mutants in PBANKA_082850 and PBANKA_120060. (B) Relative growth rates were determined using KO or C-terminal tagging vectors for the same set of genes. Phenotype calls are color coded. Green = RGR not significantly different from of 1. Red = RGR not significantly different from 0.1 Blue = Intermediate RGR.

Comparing RGR data with previously published data showed the rate of technical failure was low, as the screen correctly classified 190 of 207 known viable *P. berghei* mutants recorded in RMgmDB (Figure 2B). It additionally identified 48 viable mutants for genes that had previously been targeted without success. Most of these new viable mutants had attenuated growth phenotypes, which provides a rationale for previous failed attempts to create and clone these mutants. Ten of these unexpectedly dispensable genes were selected for individual experimental validation and all were confirmed as dispensable in single transfections (Figure S2A), suggesting the longer homology arms of *Plasmo*GEM vectors increase targeting efficiency and reveal phenotypes previously masked by technical failure. In such past experiments, locus inaccessibility may have been mistaken for gene essentiality. We tested this by transfecting matched C-terminal tagging vectors for one pool of mutants (Figure S2B). These were able to target the majority of essential genes, indicating that essential loci were generally accessible. In summary, experimental validation and benchmarking against published data lead us to conclude that only 2%–3% of all genes in the screen are misidentified as essential. Estimates for how many essential genes were falsely identified as dispensable are more difficult to generate, because very few *Plasmodium* genes have been confirmed as essential by strong experimental evidence due to the relatively recent development of tightly regulatable conditional genetic systems. However, we believe the number of false dispensable genes to be low, as the list of essential genes concurred with previous findings. The screen correctly recognized ribosomal genes and known drug targets as important for parasite growth (Figure 2C; Table S3), and the ratio of sexual versus asexual expression of a gene (Figure 2D; Table S4) was a strong predictor of phenotype in the asexual blood stage, as would be expected. Genes with high expression in asexual blood forms were characterized by very low dispensability of ∼25%, rising to 88% in a cluster of 209 genes with highest expression in gametocytes (Figure 2E). In summary, we conclude that RGR phenotypes are reproducible and reflect biological function for a large majority of genes.

Of 2,578 mutants, just two grew significantly faster than wild-type parasites. The top hit (RGR CI: 1.15–1.34) is the knockout of *ap2-g*, the master regulator of gametocytogenesis, a transcription factor that functions as master regulator of gametocytogenesis by diverting a proportion of replicating asexual parasites into non-proliferative gametocytes (Sinha et al., 2014). Spontaneous mutations in *ap2-g*, which increase their rate of asexual growth, occur if parasites are not regularly transmitted through mosquitoes to select against loss of gametocyte production (Sinha et al., 2014). A second gene, PBANKA_145120 (RGR CI: 1.01–1.25) is barely significant and may represent a false positive. Other genes required for gametocyte formation (Boisson et al., 2011, Guttery et al., 2014) do not provide a growth advantage in the current screen because they act after the point when commitment to sexual development has become irreversible. This scarcity of fast growing mutants was expected, because kin selection would create a strong evolutionary pressure to minimize mutational targets that allow parasites to grow faster at the expense of ending transmission from a host.

**Figure S3:**
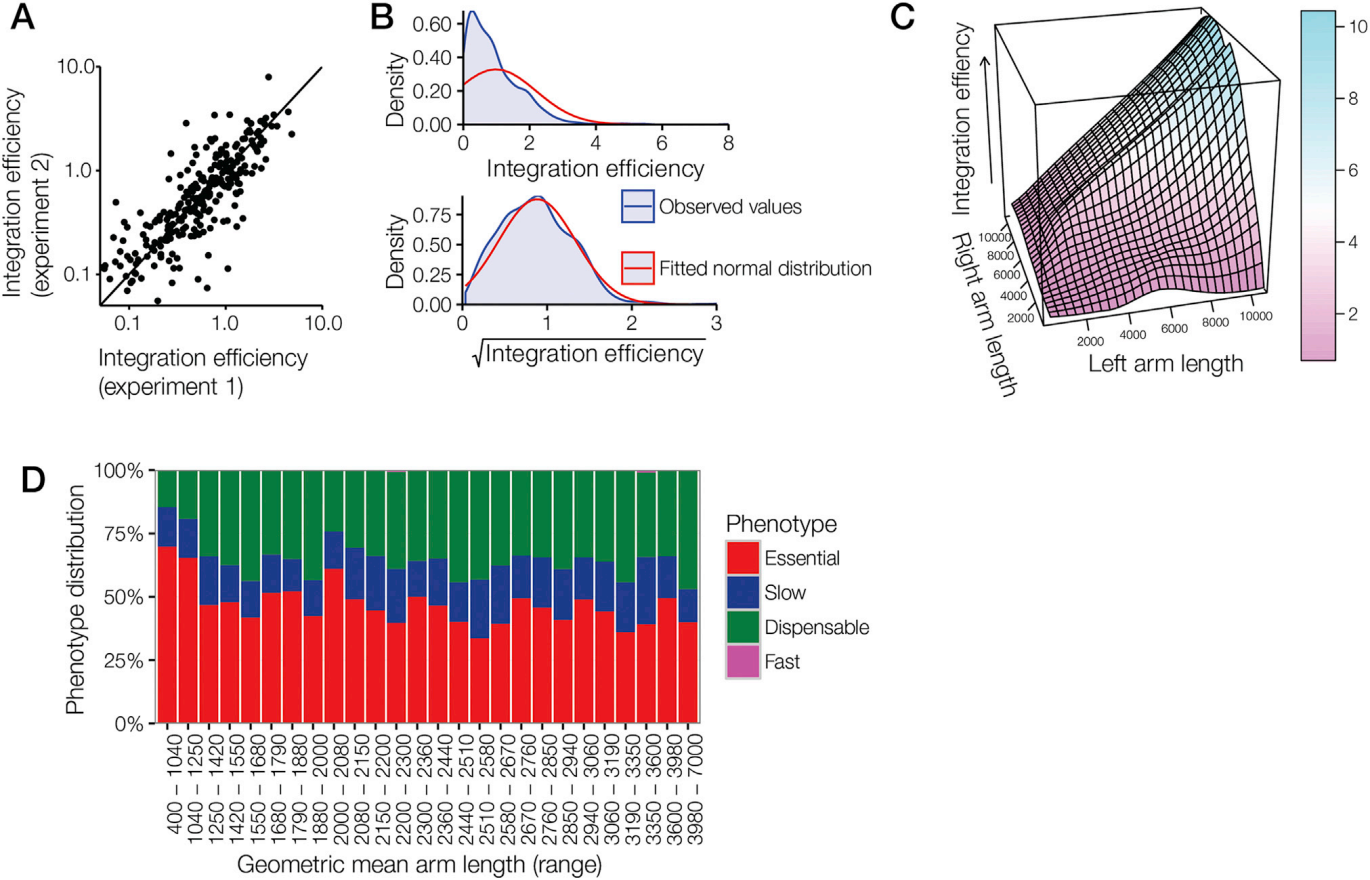
Vector Properties Determine Homologous Integration Rates, Related to Figure 2 Vector-specific integration efficiencies were calculated for the set of 915 dispensable genes by normalizing the relative abundance of a mutant during the infection to the relative abundance of the vector measured from the electroporation cuvette, and by using the four normally growing controls to normalize between experiments. (A) Vector integration efficiencies were highly reproducible between independent experiments (log-log R^2^: 0.76). (B) Relative abundances of dispensable mutants became normally distributed after applying a square root function, suggesting targeting efficiency might be the result of two independent variables interacting in a multiplicative fashion. (C) Modeling the effect on targeting efficiency of homology arm lengths, which in the PlasmoGEM resource varies from 400 bp to 14.8 kb. Initial analyses revealed the length of each homology arm to be independently linked with integration efficiency. This effect plateaus at around 5 kb due to the confounding fact that the lengths of the two homology arms are inversely correlated, since they trade off against each other for space on the vector. The graph shows a three dimensional model fitted to the data, and illustrates increasing targeting efficiency of vectors with arm length up to at least 10 kb. The product of homology arms lengths explained around 60% of the overall variation in targeting efficiency (log-log R^2^: 0.42). The remaining non-stochastic variation may be due to DNA structure and chromatin state, but combining a number of data sources with machine learning approaches failed to model these factors to improve predictive accuracy. (D) Assessment of calculated phenotypes across a range of geometric-mean homology arm lengths (groups are, as far as possible, of equal sizes). There is an even phenotype distribution across the space of homology arms, with the possible exception of a technical bias toward essential calls for vectors with a geometric mean homology arm length less than 1.25 kb. As a result, this set of vectors was discarded when calculating overall genome essentiality.

A total of 915 mutants lacked a significant growth phenotype, but their relative abundance nevertheless varied reproducibly over two orders of magnitude (Figure S3A), mainly because vector-specific rates of homologous recombination were determined by the length of their homology arms (Figures S3B and S3C). Integration efficiency was poor for a small number of vectors with geometric mean arm length below 1.25 kb, the minimum arm length for robust BarSeq screening (Figure S3D), but continued to increase with increasing arm length up to at least 10 kb (Figure S3C). The phenotype distribution shown in Figure 2A was calculated with 200 suboptimal vectors omitted to avoid biasing the result toward essential genes.

### Phenotype Distribution Is Determined by Phylogeny, Not the Experimental System Used

**Figure 3:**
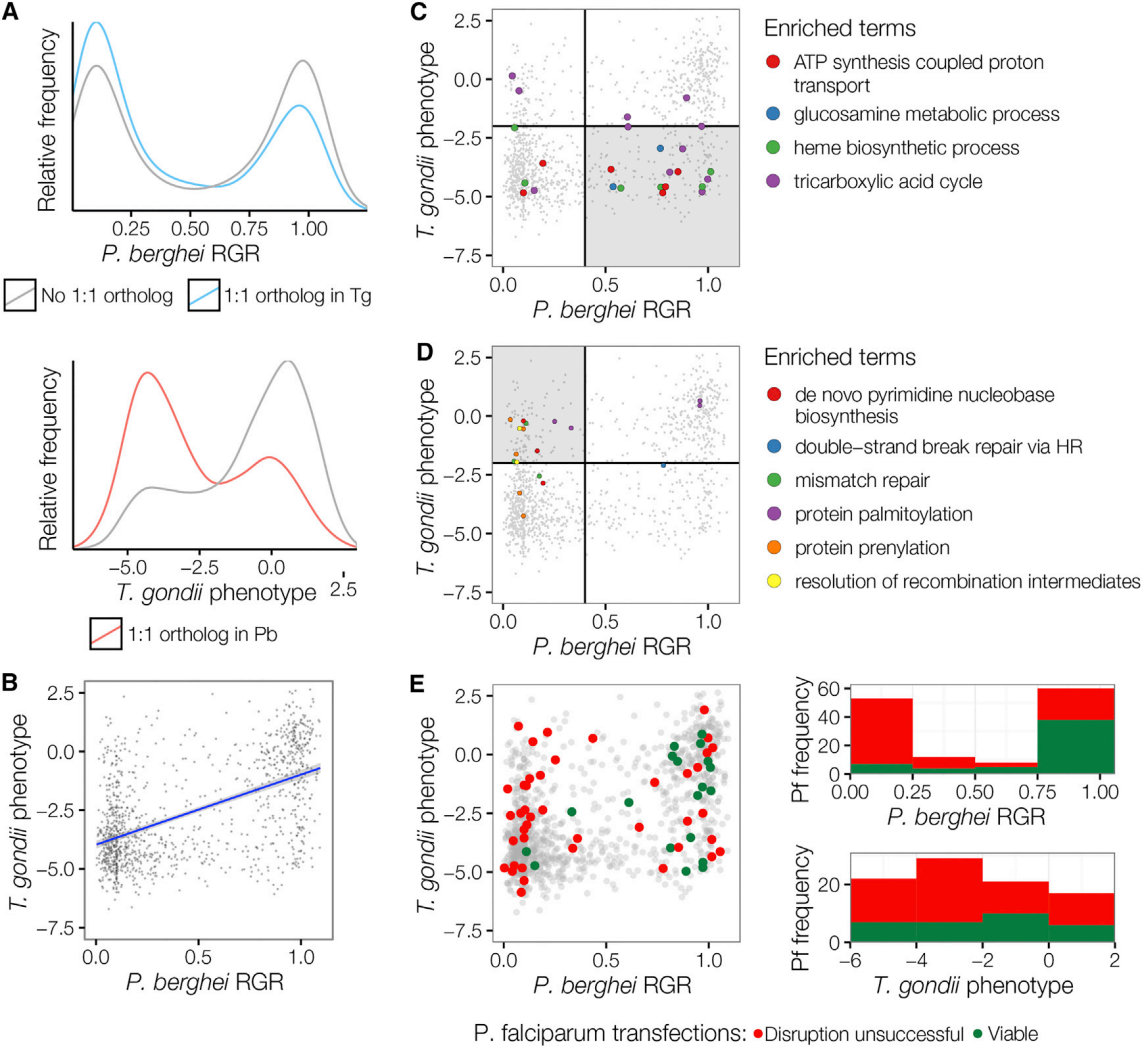
Comparison of Phenotypes for Orthologous Genes across Three Apicomplexan Species (A) The distribution of 1:1 orthologous genes in *P. berghei* and *T. gondii* (data from Sidik et al., 2016) is significantly shifted toward essentiality as compared to non-shared genes. (B) There is highly significant correlation of phenotypes between species for orthologous genes, but there are also numerous genes without conserved phenotypes. (C) All significantly enriched GO terms among genes that are more dispensable in *P. berghei* (shaded area). (D) Same as in (C) but for genes more dispensable in *T. gondii* (shaded area). (E) Left: data as in (B) but overlaid with published *P. falciparum* phenotypes (Sanderson and Rayner, 2017). Right: data from pairwise comparisons. *P. falciparum* phenotypes are color coded (green, viable mutant; red, confirmed essential or disruption failed). See also Table S5.

A recent CRISPR screen in *T. gondii* grown in cultured human cells identified a much lower proportion of genes contributing to normal growth (Sidik et al., 2016). The higher rate of essential and slow growing mutants in our screen, which was carried out in vivo, raised the possibility that in vitro systems may fail to reveal phenotypes for many loss-of-function mutations. If true, this would limit the utility of the important and widely used in vitro culture system for *P. falciparum*, so we systematically compared the *P. berghei* and *T. gondii* data. Of 2,034 genes with 1:1 orthologs in *P. berghei* and *T. gondii*, 1,140 have phenotypic data in both screens (Table S5). The proportion of genes important for normal growth is increased in this conserved set (Figure 3A), but gene functions are only moderately correlated between species overall (Figure 3B). GO terms enriched in genes with discordant phenotypes (Figures 3C and 3D) either recapitulate known biological differences between species, such as an increased importance of mitochondrial energy metabolism in *T. gondii* (essential tricarboxylic acid cycle) (MacRae et al., 2012, Ke et al., 2015), confirm anticipated results (homology-based repair of double strand breaks is more important in *P. berghei*, which lacks the alternative NHEJ pathway) (Kirkman et al., 2014), or reveal unexpected differences (e.g., less redundancy among prenylation and palmitoylation enzymes in *P. berghei*).

The considerable level of disagreement between phenotypes in *T. gondii* and *P. berghei* might reflect differences between taxa or between experimental conditions (in vitro versus in vivo). We therefore asked which of these screens best agreed with a curated database of *P. falciparum* gene disruption attempts (Sanderson and Rayner, 2017), all conducted in vitro. We saw that genes important for growth in *P. berghei* were almost invariably impossible to disrupt in *P. falciparum* (50 out of 57 genes), irrespective of their phenotype in *T. gondii* (Figure 3E). When the analysis was extended beyond 1:1:1 orthologs to all homologous pairs (Figure 3F), *P. berghei* remained by far the best predictor of gene function in *P. falciparum*. While many dispensable *P. berghei* genes do have failed disruption attempts reported in *P. falciparum*, we interpret this as reflecting the more challenging genetic system in *P. falciparum.* Collectively, this analysis shows that the high number of essential genes in *P. berghei* probably extends to *P. falciparum*, and it reflects the selection pressures under which parasite genomes evolve, rather than the experimental system under which they are assayed.

### Opposing Evolutionary Pressures Are Shaping the *Plasmodium* Genome

**Figure 4:**
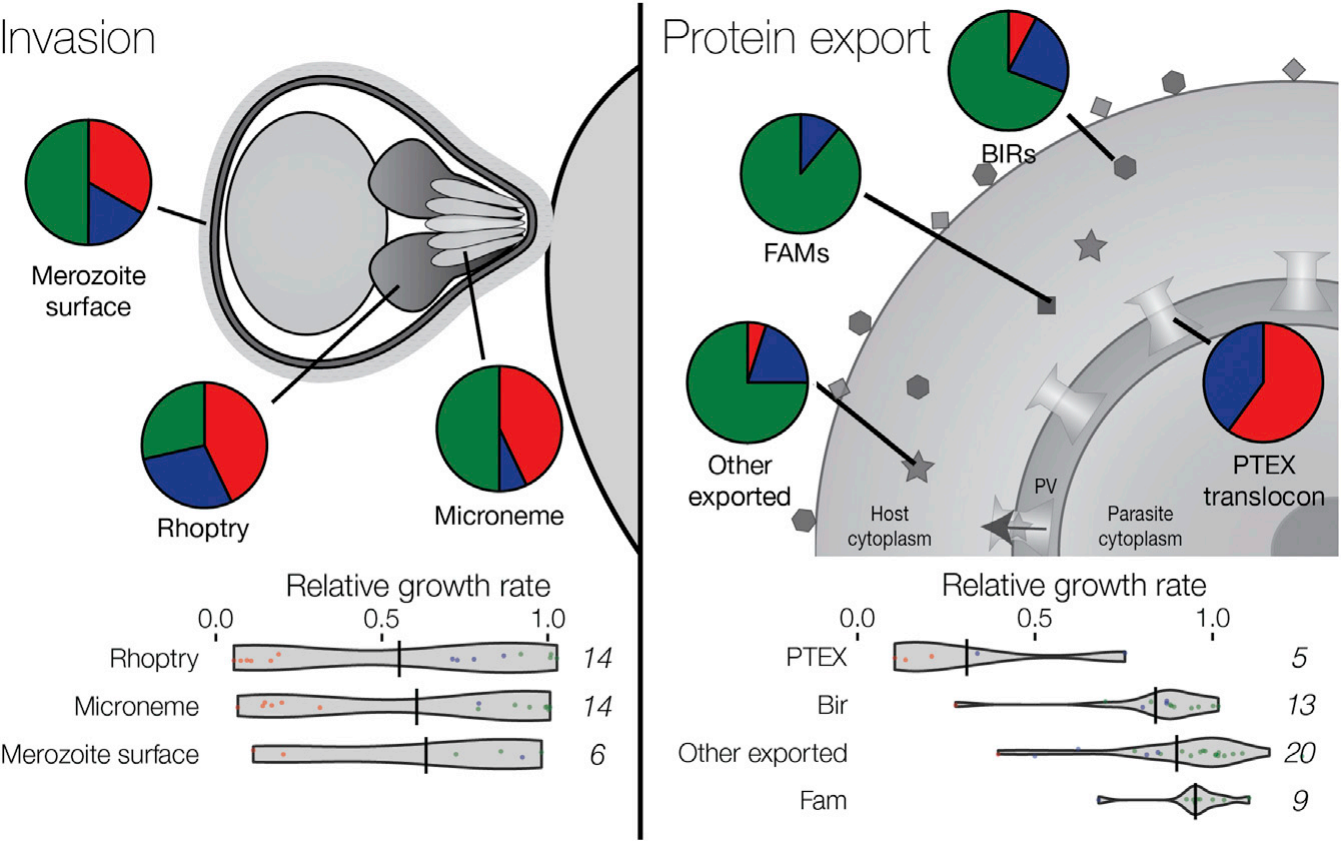
Reduced Essentiality among Genes Involved in Direct Interactions with the Host Phenotype distributions for secreted and surface proteins of the invasive merozoite and for proteins exported from the asexual intraerythrocytic parasite are shown as pie charts using the same colors as in Figure 2. Gene numbers in each category are next to violin plots showing RGR. Enrichment in dispensable genes is significant for *bir* (p < 0.02), *fam*, and “other exported” (both p < 0.01). See also Table S3.

The high proportion of essential genes in *P. berghei* seems at odds with the fact that many well-studied aspects of *Plasmodium* pathogenesis involve expanded gene families that allow parasites to use alternative erythrocyte invasion pathways (e.g., Duraisingh et al., 2003) or mechanisms for cytoadherence and immune evasion (Maier et al., 2008). Consistent with current concepts, we find that many proteins on the merozoite surface or secreted from micronemes during host cell invasion are dispensable (Figure 4; Table S3). Gene products exported into the host erythrocyte contain an even larger proportion of dispensable genes, while the conserved machinery for exporting these proteins from the parasite (plasmodium translocon of exported *proteins* [PTEX]) is predictably important (Figure 4).

**Figure 5:**
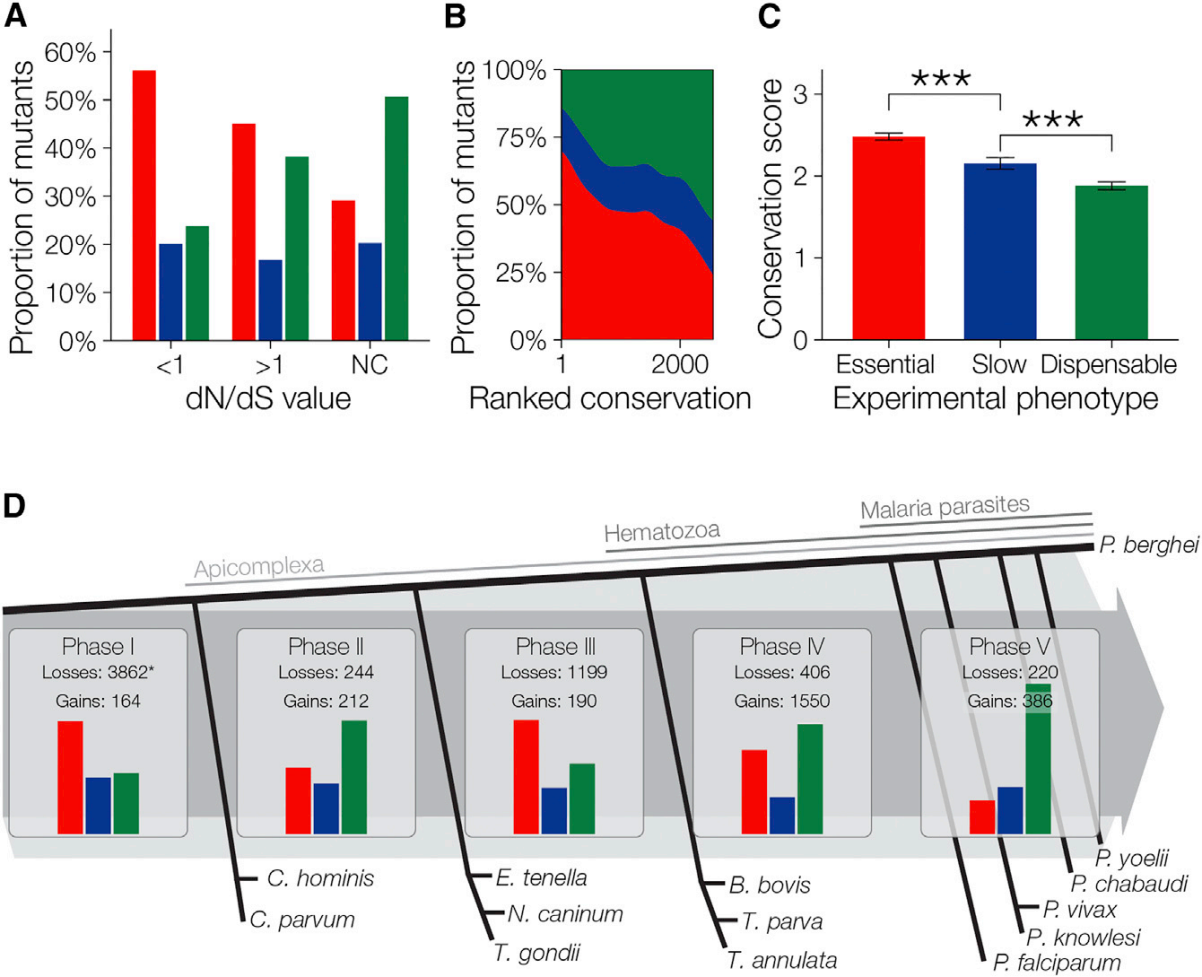
Genomic Reduction and High Gene Essentiality during the Evolution of Hematozoa (A) Phenotype distributions for the *P. berghei* orthologs of genes under purifying selection in *P. falciparum* (dN/dS <1) and genes potentially under positive selection in *P. falciparum* (dN/dS >1) compared to *P. berghei* genes without orthologs in *P. falciparum*. (B) Genes highly conserved between *Plasmodium* species are enriched for essential phenotypes. Genes are ranked by their conservation between *P. chabaudi* and *P. falciparum* (according to the MalariaGEN Plasmodium falciparum Community Project, 2016) with the phenotype distribution of *P. berghei* orthologs and different conservation levels plotted. (C) Phenotype is significantly predictive of inter-species conservation score (^∗∗∗^p < 0.0001). (D) The evolutionary history of *P. berghei is* shown through the gain and loss of groups of orthologs as reconstructed using a Dollo parsimony model (^∗^ taken from Woo et al. [2015]). Bar charts show relative distribution of *P. berghei* phenotypes among extant genes belonging to the orthologous groups gained during each phase: Phase I, from the free-living protoapicomplexan to the first apicomplexan; Phase II, from the first apicomplexan to the ancestor of the piroplasms and coccidians; Phase III, from this ancestor to the first hematozoan; Phase IV, from the first hematozoan to the first malaria parasite; and Phase V, from the first malaria parasite to *P. berghei*. See also Table S6.

Most redundant gene products acting at the parasite-host interface have evolved recently in the genus, and we therefore asked more generally how the evolutionary history of genes was related to their current phenotype. Genes under purifying selection in African populations of *P. falciparum*, or characterized by a higher degree of amino acid conservation, contribute more strongly to normal growth in *P. berghei* (Figures 5A–5C; Table S6). Looking beyond the genus *Plasmodium*, the history of genes during apicomplexan evolution predicted gene function in *P. berghei* more generally. In particular, genes acquired during two major genomic reduction events, i.e., the loss of 3,862 ortholog groups during the emergence of the Apicomplexa and then the loss of a further 1,199 with the acquisition of an intraerythrocytic lifestyle by the Hematozoa (Woo et al., 2015), were much more likely to contribute to asexual stage growth than genes acquired at other points during the ancestral lineage leading to *Plasmodium* (Figure 5D). A substantial group of 1,174 genes has resisted gene loss and are present in all hematozoan genomes analyzed to date. Eighty-six percent of this large class contributes to blood stage growth, including many functionally unannotated genes (Table S6), which may have conserved roles in the erythrocytic life style of Hematozoa. Together, these data link the dramatic reduction of the genome during hematozoan evolution to a marked functional optimization, probably resulting in the high degree of genetic essentiality in the conserved core genome.

### Growth Rate Phenotypes Identify Essential Biological Pathways

**Figure S4:**
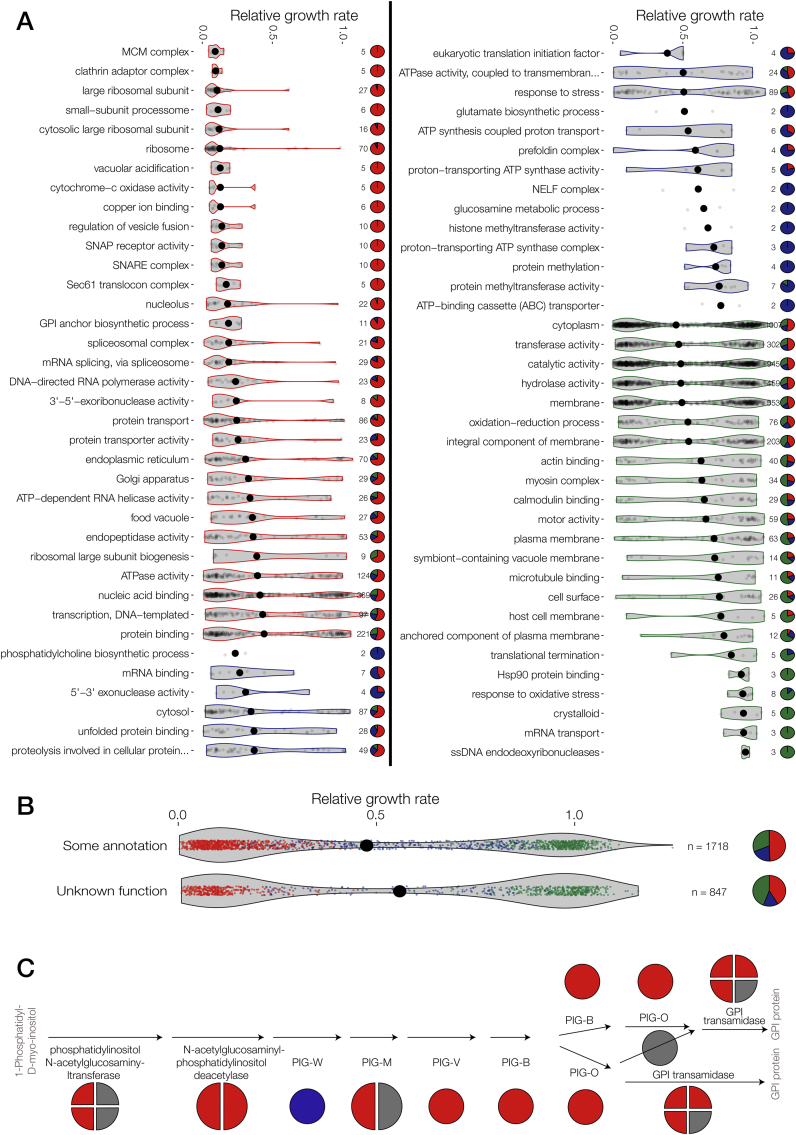
Analysis of Gene Functions Associated with Phenotypes, Related to Figure 6 and Table S7 (A) GO terms significantly enriched for genes with essential, slow or dispensable phenotype (p < 0.05). See Table S7 for genes in all significant categories. (B) Phenotypes of genes of unknown function. In both panels violin plots show RGRs, with the median indicated by a black dot. Gene numbers per category are given next to pie charts showing phenotype distributions. Color scheme as in Figure 2. (C) Phenotypes mapped onto genes involved in GPI-anchor biosynthesis.

The identification of 1,196 genes likely to contribute to normal parasite growth in vivo provides an opportunity to define the biological pathways crucial for blood-stage growth, which will help validate or deprioritize targets for future antimalarial drugs. 74 gene ontology (GO) terms and metabolic pathways are significantly enriched in essential, dispensable, or slow phenotypes (Figure S4A; Table S7). We identify potentially druggable pathways, key metabolic processes, housekeeping, and biogenesis functions. Over a third of *P. berghei* genes still lack known domains or predictable functions, but many of these are nevertheless important for normal growth (Figure S4B).

As expected, essential genes were enriched in most basic cellular processes, including transcription, mRNA splicing, translation, vesicular transport, and proteasomal protein degradation. Among the lipid metabolic pathways, glycosylphosphatidylinositol (GPI) anchor biosynthesis is the most clearly essential in blood stages (p < 0.05, Figure S4C). GPI-anchored surface proteins have important functions in specific life-cycle stages and the essential nature of the pathway, which is druggable in fungi (Mann et al., 2015), may thus provide targets for multi-stage inhibitors. Enzymes involved in phosphatidylcholine biosynthesis, which are thought to include the targets of bis-thiazolium drugs (Wengelnik et al., 2002) were associated with severely reduced growth (p < 0.05). In marked contrast, although *Plasmodium* blood stages can synthesize sphingolipids de novo and the pathway has been proposed for drug development (Pankova-Kholmyansky and Flescher, 2006), none of the implicated genes were important for normal growth in vivo, suggesting that sphingolipids may be scavenged from the host (p < 0.02).

Unstudied cellular functions enriched in attenuated growth phenotypes include biosynthetic pathways for glutamate and glucosamine (all p < 0.05), genes involved in cellular stress responses (p < 0.02), and methyltransferases with likely functions in chromatin regulation (p < 0.05), all of which may be promising targets for future drug development.

**Figure 6:**
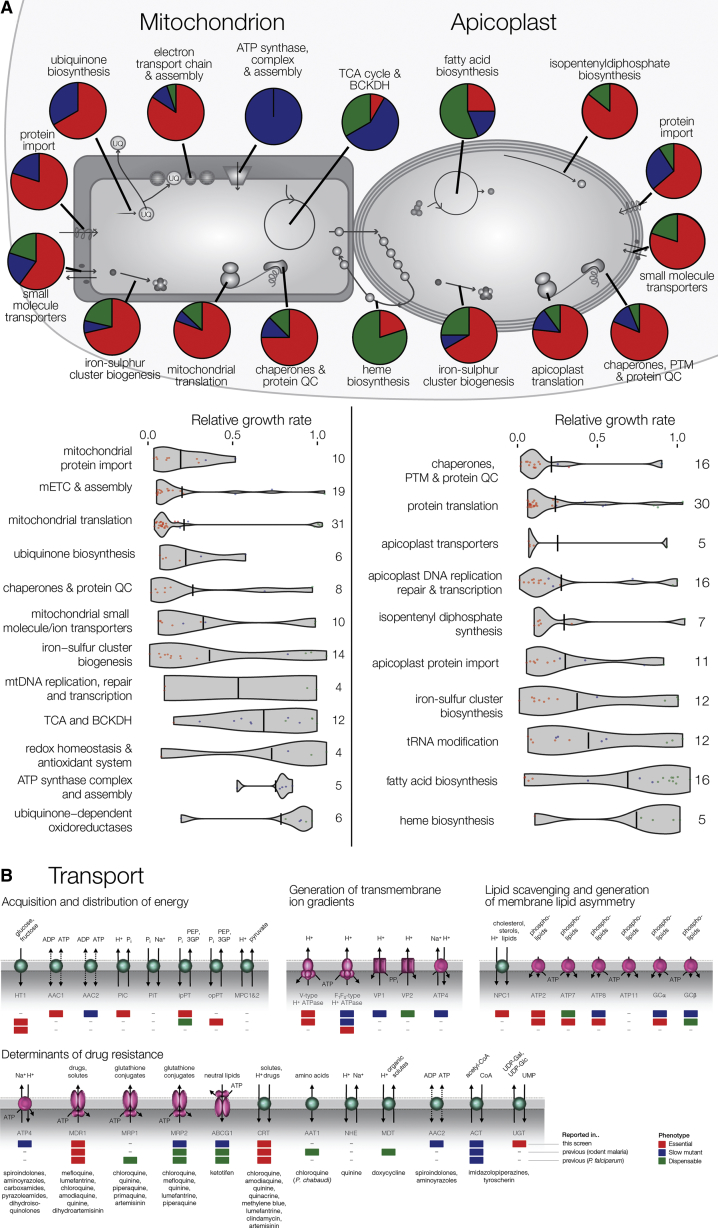
Knockout Phenotypes of Selected Organellar Pathways and Transport Functions (A) RGR and phenotype distribution of putative mitochondrial and apicoplast genes. See Table S3 for genes in each category. Violin plots show growth rate distributions with the horizontal line indicating the median. Number of genes per category is stated. (B) Knockout phenotypes of selected *Plasmodium* transporters. Dispensable, slow, and essential phenotypes are indicated by the green, blue, and red boxes, respectively. The phenotypes are from this study (top box) and from published *P. berghei* (middle box) and *P. falciparum* (bottom box) studies. Pumps (i.e., primary active transporters) are shown in pink and carriers (i.e., uniporters, symporters, and antiporters) in aqua. Examples of the antimalarial drugs affected by the resistance determinants are listed below each transporter. See also Figure S4 and Tables S7 and S8.

Apicomplexan parasites possess a highly derived mitochondrial genome, which could indicate adaptation to parasitic existence and is reflected in a reduced set of mitochondrial metabolic pathways relative to model organisms (Vaidya and Mather, 2009). *Plasmodium* mitochondria are nevertheless essential, housing parts of critical pathways supplying molecules essential for nucleic acid metabolism, DNA replication, DNA repair, transcription, and maturation of various components of the parasite’s translational apparatus (Figure 6A). The mitochondrially located enzyme dihydroorotate dehydrogenase is important for the synthesis of pyrimidines and is supported by the mitochondrial electron transport chain (mtETC). Other metabolic processes depend on a number of enzymes requiring iron sulfur cofactors, which are ultimately supplied by the mitochondrial iron-sulfur cluster biogenesis system. Thus, dihydroorotate dehydrogenase, many components of the mtETC and assembly factors, the ubiquinone biosynthesis pathway, and the iron-sulfur biogenesis pathway are essential, as are the supporting maintenance machinery, including mtDNA polymerase, mitochondrial ribosomes, the protein import machinery, and several transporters.

Asexual blood-stage parasites obtain most of their ATP via aerobic glycolysis in the cytoplasm, and glycolytic enzymes are largely essential as expected. Conversion of pyruvate from glycolysis to mitochondrial acetyl-CoA in Apicomplexa is mediated by the branched-chain keto acid dehydrogenase (BCKDH) complex (Oppenheim et al., 2014), all tested subunits of which have an attenuated growth phenotype (Figure 6A). Similarly, the mitochondrial tricarboxylic acid (TCA) cycle, which uses acetyl-CoA to power the mitochondrial electron transport chain, and the ATP synthase complex, are significantly enriched for slow growth phenotypes, although some of its enzymes are entirely dispensable for asexual blood stage growth, as reported previously for *P. falciparum* (Ke et al., 2015).

Malaria parasites have a relic plastid known as the apicoplast, which has lost the ability to photosynthesize, but fulfils crucial biosynthetic functions. Our screen confirms chemical supplementation of in vitro cultured blood stage *P. falciparum* that showed isopentenyl pyrophosphate (IPP) synthesis as a critical apicoplast function in erythrocytes (Yeh and DeRisi, 2011) (Figure 6A, p < 0.05), as well as Fe:S cluster synthesis, which supports IPP synthesis enzymes. Conversely, fatty acid synthesis, which is only essential during the liver phase in *P. berghei* (Shears et al., 2015), is clearly dispensable (p < 0.1). Heme synthesis is shared across the mitochondrion, cytosol, and apicoplast and is essential during the mosquito phase of the *P. berghei* life cycle but not the blood phase (Sigala and Goldberg, 2014), and we find it enriched for dispensable phenotypes (p < 0.1). The machinery to replicate, repair, transcribe, and translate the apicoplast genome, which encodes ∼30 proteins, is essential (p < 0.1–0.001) (Figure 6A). Also crucial, is the machinery to import ∼500 nucleus-encoded, cytosol-synthesized apicoplast proteins and chaperones and proteases to maintain quality control of these proteins (p < 0.01). Apicoplast transporters, most with unknown but apparently critical roles, are similarly indispensable. Our screen flags hundreds of priority apicoplast targets, all of which are prokaryotic in ancestry and thus predisposed to high selectivity against the human host equivalents in a similar vein to antibacterials. Table S3 lists the predicted components of apicoplast and mitochondrial pathways and their growth phenotypes.

In a parasite whose metabolism is intertwined with that of the host, solute transporters can assume particular significance, and our screen confirms that among the 79 targeted transporters of known and unknown specificity (Martin et al., 2009) 26 are essential and a further 19 are required for normal growth. Unsurprisingly, many of the transporters that support fundamental requirements of the cell, such as the acquisition and distribution of energy and the generation of transmembrane ion gradients, are essential or have slow growth phenotypes (Figure 6B). When considered alongside published knockout phenotypes (Table S8), the data also reveals that a number of the transporters with putative roles in lipid scavenging and the generation of membrane lipid asymmetry are required for normal growth (Figure 6B).

Transporters also play a central role in drug resistance, and to date, 12 *Plasmodium* transporters have been implicated as drug targets and/or mediators of resistance (Table S8). The essentiality of three of the transporters (CRT, MDR1, and UGT) was not unexpected (Figure 6B) given that resistance-conferring polymorphisms in these proteins impart fitness costs to the parasite (Rosenthal, 2013). The conflicting evolutionary forces acting upon these resistance determinants could be exploited by rationally designed drug combinations (Summers et al., 2012). The observation that many of the resistance determinants are not essential (e.g., MRP2, ABCG1, and ACT; Figure 6B) highlights the risk in dismissing transporters with slow or dispensable phenotypes as unimportant. Dispensable transporters offer the parasite the means to develop drug resistance without incurring a significant fitness cost and thus represent opportunities for evolution and adaption.

## Discussion

Obligate intracellular parasites have, until recently, posed insurmountable technical challenges for large reverse genetic screens. Here, we have demonstrated that simultaneous phenotyping of barcoded *P. berghei* mutants offers a systematic and unbiased way of measuring phenotypes, identifying gene functions on a genome scale in an intracellular parasite in vivo. Screening a representative vector library covering more than half of all *P. berghei* protein-coding genes, we identify 1,652 genes required for parasite growth in mice. By extrapolation, we predict 62.9% of the core *P. berghei* genome to be important for normal development during asexual blood stages. This is in marked contrast to studies in free-living model organisms and other parasites (Table 1) reporting a high degree of functional redundancy, which have posed a quandary because all genes must be subject to selection pressures that cause them to be retained throughout long periods of evolution.

For multicellular organisms, an explanation for the missing phenotypes was offered by data showing that most loss-of-function mutations had subtle effects on overall fitness that were only revealed either by using a broad phenotyping pipeline, as in the mouse (White et al., 2013), or by letting *C. elegans* mutants compete over many generations (Ramani et al., 2012). In yeast, substantially more phenotypes were revealed in genetic-interaction screens. Disrupting genes in a pairwise fashion identified phenotypes for most yeast genes, which gave rise to the idea that dispensable genes can provide a buffer for genetic mutations (Boone et al., 2007) allowing these to persist longer and recombine within the gene pool of a sexually-reproducing population. *Plasmodium* genomes are very polymorphic (Manske et al., 2012), and the abundance of essential genes in *P. berghei*, which undergoes obligatory sexual reproduction and is haploid in the blood stages, suggests that buffering does not play a significant role in the *Plasmodium* genome.

An alternative explanation for the abundance of dispensable genes in most eukaryotes, compared to the much reduced number in *P. berghei*, is that the robustness of genetic networks reflects an ability to survive in different environments. That most mutants will reveal a phenotype if assayed under the right condition is supported by screens of haploid yeast mutant collections under many different conditions (Hillenmeyer et al., 2008), but we have shown here that the highly-reduced *Plasmodium* genome does not pose a missing-phenotype quandary. However, parasites experience less environmental variation than most free-living organisms because their hosts act as homeostats that control their internal milieu within narrow physiological parameters, which is thought to lead to the strong trend among parasites toward genomic reduction. Our data support the model that this limited environmental variation is also responsible for the lack of functional redundancy in the *Plasmodium* genetic network.

The high degree of gene essentiality in *P. berghei* is not simply a feature of a pathogenic lifestyle per se. Screening of dense transposon libraries of *Salmonella enterica* serovar Typhimurium, for instance, has revealed that only ∼7.5% of genes are required for growth in rich medium (Barquist et al., 2013), rising to an estimated 24%–36% for effective intestinal colonization (Chaudhuri et al., 2013). In the extracellular blood parasite, *Trypanosoma brucei*, whose genome carries relatively fewer signs of genomic reduction (Jackson et al., 2016), only 36.6% of 7,435 genes contribute to normal growth in culture (Alsford et al., 2011), and in the obligate intracellular parasite, *T. gondii*, only ∼40% of ∼8,000 genes contribute to normal growth in cultured human fibroblasts (Sidik et al., 2016) and far fewer are thought to be truly essential. *T. gondii* tachyzoites can replicate in a wide range of tissues in birds and mammals, which maximizes their chances of transmission. Environmental variation may thus provide an explanation for both their larger genome and greater proportion of dispensable genes.

An alternative explanation for differences in phenotype distribution between *P. berghei* and *T. gondii* could be the use of an in vitro culture model by Sidik et al. (2016). In the absence of an efficient culture system in *P. berghei,* we could not test this possibility directly, but a comparison with published data for 324 *P. falciparum* genes generated in vitro revealed no evidence that culturing *Plasmodium-*infected erythrocytes would fail to detect large numbers of functionally-important parasite genes. This is reassuring because cell-based screens for antiplasmodial compounds rely heavily on *P. falciparum* in vitro culture models. It should be noted that the relatively small curated *P. falciparum* essential gene set may not be representative of the genome as a whole, and suspected essentiality of a *P. falciparum* gene often relies on anecdotal evidence that a gene cannot be disrupted. Despite these caveats, which illustrate the value of this first systematic genetic screen in a *Plasmodium* parasite, these considerations collectively support our interpretation that a high proportion of genes contributing to normal growth may coincide with the genomic reduction in the evolutionary lineage that led to the acquisition of an intraerythrocytic life style by the ancestral hematozoan (Woo et al., 2015). It is also intriguing that genome-scale knockout screens from protozoan parasites consistently identify ∼3,000 genes as important for normal growth of a single life-cycle stage (Table 1), regardless of the particular assay and genetic methodology used.

To find that parasite genes whose products interact directly with the host are more likely to be dispensable is no surprise, but is shown here systematically for the first time. Antigenic diversification and functional redundancy represent the response of the parasite to a changeable environment that results not only from the adaptive immune response of the host, but also from an ongoing evolutionary arms race thought to have given rise to the human ABO blood group system (Cserti and Dzik, 2007), among other polymorphisms. They also pose major challenges to malaria vaccine development (Crompton et al., 2010). Importantly, however, we also show the evolutionary trend toward functional redundancy affects only a small and well-defined part of the genome, while the majority of genes contribute significantly to normal asexual growth. This means there must be substantially more druggable targets in *Plasmodium* than, for instance, in bacteria.

The fact that *P. berghei* needs to deploy most of its reduced genome to grow optimally suggests the transcriptome must be similarly optimized, which may explain the oft-noted limited capacity of *P. falciparum* to mount adaptive transcriptional responses (e.g., Rovira-Graells et al., 2012). Even more importantly, it means that most genes must function at multiple points in the life cycle, which helps rationalize why many antiplasmodial compounds discovered through their ability to kill cultured blood stages can also function as the multi-stage drugs now considered crucial for malaria eradication (Burrows et al., 2017). Recent progress in this area can now be understood in the light of a high degree of genetic essentiality in the *Plasmodium* genome.

Further experimental support for the concept of abundant pleiotropic functions of *Plasmodium* genes will require the development of a knockout system that is both scalable and inducible to identify essential gene functions systematically at multiple life-cycle stages. In the meantime, BarSeq, with the available vector library, will enable detailed phenotyping of slow growing mutants, screens for other blood stage phenotypes, and functional characterization of dispensable genes at other life-cycle stages.

## STAR★Methods

### Key Resources Table

**Table:** 

REAGENT or RESOURCE	SOURCE	IDENTIFIER
**Critical Commercial Assays**		

Amaxa P3 Primary Cell 4D-Nucleofector X Kit S	Lonza	V4XP-3032
4D-Nucleofector Core Unit	Lonza	AAF-1002B
4D-Nucleofector X Unit	Lonza	AAF-1002X
MiSeq Reagent Kit v2 (300-cycles)	Illumina	MS-102-2002
MiSeq Sequencing System	Illumina	N/A

**Deposited Data**		

*P. berghei* relative growth rate phenotypes	This paper	http://plasmogem.sanger.ac.uk/phenotypes

**Experimental Models: Cell Lines**		

Arrayed library of *E. coli* TSA cells harboring linear plasmids containing *P. berghei* gene targeting vectors.	*Plasmo*GEM resource (http://plasmogem.sanger.ac.uk/search)	Table S1

**Experimental Models: Organisms/Strains**		

Rat: RCC Han Wistar outbred (female)	Envigo+++	RccHan:WIST
Mouse: BALB/c inbred (female)	WTSI & Envigo+++	BALB/cOlaHsd
*P. berghei:* ANKA cl15cy1 wild-type parasites	N/A	cl15cy1
*E. coli:* BigEasy-TSA	Lucigen	60224

**Recombinant DNA**		

BigEasy v2.0 Linear Cloning Kit (pJAZZ-OK Blunt Vector)	Lucigen	43036

**Sequence-Based Reagents**		

Oligonucleotide primers for barcode amplification and index tagging	Gomes et al. 2015	http://plasmogem.sanger.ac.uk/info/primers
Oligonucleotide primers for qPCR based genotyping	N/A	http://plasmogem.sanger.ac.uk/search

**Software and Algorithms**		

R	https://www.r-project.org	3.3
topGO	Bioconductor	3.4
Count	http://www.iro.umontreal.ca/∼csuros/gene_content/count.html	N/A

### Contact for Resource and Reagent Sharing

All requests may be directed to Oliver Billker (ob4@sanger.ac.uk). *Plasmo*GEM reagents are freely available under a material transfer agreement for not-for-profit research and should be requested directly from the *Plasmo*GEM resource (http://plasmogem.sanger.ac.uk/request/howto).

### Experimental Model and Subject Details

#### Parasites and animals

All experiments used the sequenced reference clone cl15cy1 of *P. berghei* ANKA. All animal research was conducted under licenses from the UK Home Office, and protocols were approved by the Animal Welfare and Ethical Review Body of the Wellcome Trust Sanger Institute. Rodents were kept in specific-pathogen-free conditions and subjected to regular pathogen monitoring by sentinel screening. They were housed in individually ventilated cages furnished with autoclaved aspen woodchip, fun tunnel and Nestlets at 21 ± 2°C under a 12:12 hr light-dark cycle at a relative humidity of 55 ± 10%. They were fed a commercially prepared autoclaved dry rodent diet and water, both available ad libitum. The health of animals was monitored by routine daily visual health checks. The parasitemia of infected animals was determined by methanol fixed and Giemsa-stained thin blood smears.

Female RCC Han Wistar outbred rats (Envigo, UK) aged eight to fourteen weeks were infected with *P. berghei* wild-type parasites by intraperitoneal injection. Infected rats served as donors for ex vivo schizont cultures typically on day four to five of infection, at a parasitemia of ∼1%–5%. Rats were housed with two cage companions. Rats were terminally anaesthetised by vaporised isoflurane administered by inhalation prior to terminal bleed. Rats were used because they give rise to more schizonts with higher transfection efficiency compared to mice. Transfection efficiency is critical when screening pools of vectors.

Mice were bred at WTSI or purchased from Envigo. Transfected parasites were injected intravenously into the tail of female adult BALB/c inbred mice aged 8-22 weeks (median age 10 weeks). This animal model was chosen to minimize host genetic variability and to obtain robust infections with a low incidence of cerebral pathology. Experimental groups consisted of three mice housed together. Three internally controlled biological replicates per parasite pool proved adequate to identify phenotypes with confidence.

### Method Details

#### Pooled transfections

Each of 2578 knockout vectors was assigned to one or more vector pools, creating a total of 58 pools of vectors. For each pooled transfection, vectors were prepared and transfected largely as described (Gomes et al., 2015) with minor modifications. Briefly, groups of ∼96 targeting vectors were prepared in parallel by growing 1 mL liquid cultures inoculated from glycerol stocks into duplicate 96X deep-well plates, incubated shaking at 37°C for 16 hr. Saturated cultures were then pooled and DNA extracted in a single midiprep reaction (QIAGEN Plus Midi Prep Kit) using one column per plate and pooling DNA from duplicates after purification. A total of 30 μg (approximately 100 ng of each vector, in triplicate), including spike-in DNA of control vectors, was digested overnight with NotI to release the targeting vector from the linear plasmid backbone.

The universal control vectors included in each transfection were as described previously (Gomes et al., 2015). Briefly, four sexual stage specific genes (*p25*, *p28*, *p230p* and *soap*) had wild-type growth phenotypes and their weighted mean growth rate on a given day was defined as 1. Three additional controls were chosen for their known reduced growth phenotypes (Gomes et al., 2015). Identification numbers for all vectors included in this study are shown in Table S1 and can be used to access details of each vector design, including quality control data, through the *Plasmo*GEM database (http://plasmogem.sanger.ac.uk).

Following restriction digests the vector pools were purified by ethanol precipitation and DNA for each triplicate pool was resuspended in 18 μL of nuclease-free water. Rat derived *P. berghei* schizonts were harvested after 22 hr in culture and purified on a Histodenz (Sigma) gradient. Purified parasites were pelleted at 450 g for 3 min, resuspended in 54 μL P3 Primary Cell 4D-Nucleofector solution (Lonza) and added to the DNA solution. Of this mixture exactly 26 μL were transferred into each of three separate wells of a 16X 4D-Nucleofector strip cuvette. Cells were electroporated using the FI115 program on the 4D-Nucleofector core system equipped with an X Unit (Lonza). Transfectant parasites were injected intravenously into mice and selected with 0.07 mg / mL pyrimethamine in drinking water (pH ∼4.5) from day one post infection (p. i.).

#### Measuring relative growth rates

Infections were sampled at the same time of each day between days four to eight p. i., except in a few instances, when mice developing signs of disease on day seven led to early termination of the experiment. Transfection mixture from the electroporation cuvettes was also sampled to verify pool compositions, so that only vectors verified in the input were included in the analysis.

To measure in vivo growth, we used the gene-specific 11 base pair barcodes included in *Plasmo*GEM vectors (Schwach et al., 2015), which were amplified linearly from genomic DNA extracts and counted on an Illumina MiSeq.

Parasite genomic DNA was extracted from tail blood samples collected daily, using the phenol/chloroform method, as previously detailed and resuspended in 50-100 μL nuclease-free water. Barcodes present in each of these samples were counted through Illumina sequencing. For that, amplicon-based Illumina sequencing libraries were prepared using a nested PCR approach that targeted constant regions flanking each gene-specific 11 nt barcode. See the Key Resources Table for information in index primers used. The resulting libraries consisted of 234 bp long amplicons containing sample-specific indexes that were pooled equimolarly, typically in groups of 32 these were then sequenced using the MiSeq Reagent Kit v2 (300 cycle) from Illumina (MS-102-2002) and diluted to 4 nM prior to loading at low cluster density (4x10^5^ clusters/mm^2^) with ∼40% of PhiX spike-in.

Validation of gene disruption for selected vectors was achieved using individual transfections and quantitative polymerase chain reaction (qPCR). Parasites were transfected as described previously (Gomes et al., 2015) with 1-5 μg of a single *Plasmo*GEM vector. Selection with pyrimethamine was initiated on day 1 post-transfection. On day 6 or 7 post-transfection gDNA was extracted from purified parasites using the QIAGEN Blood and Tissue kit. The presence of the locus in question was assessed by conducting a qPCR reaction with primers QCR1 and QCR2 for each vector - the template for these primers is only available if the gene is intact. Two control reactions were run in each case: one with generic primers (QCR1 and QCR2 for an unrelated gene - PBANKA_061520) on the extracted DNA to control for the number of parasite genomes in the sample, and one with gene-specific QCR1 and QCR2 on wild-type DNA to control for differential primer efficiencies. All reactions were carried out in duplicate or triplicate. Locus depletion was computed using the delta-delta Ct method. The proportion of parasites with an intact gene was then calculated as 2^-ΔΔCt^. Control reactions A and B consist respectively of the PBANKA_082850 knockout assessed with the PBANKA_120060 primers, and the PBANKA_120060 knockout assessed with PBANKA_082850 primers.

### Quantification and Statistical Analysis

#### Analysis of barseq results

It was necessary to develop a nuanced statistical approach for computational analysis of barcode data. Mutants varied in abundance, both due to differences in vector integration efficiency and because of differential fitness. This variation, as well as the sampling errors inherent to rare mutants and sampling variance associated with barcode counting, resulted in different accuracies for the inferred individual mutant fitnesses. This necessitated a statistical model to estimate the expected error for each read count measurement, and a way of propagating these uncertainties to the barcode ratios and the daily RGRs calculated from them. We then weighted RGR measurements from all days and replicates by the inverse of their variance, giving the most robust measurements the greatest impact on the combined value. Finally, where multiple measurements were available for the same gene, these were combined, again weighted by inverse variance.

##### Procedure

Raw reads from the sequencer were separated based on their sample (a specific mouse infected with a specific pool and sampled on a certain day, or the transfection input), as represented in Illumina index tags. The sequence data from each sample were analyzed by a script which selected reads with correct flanking sequences and then counted the barcodes carried between them. Only perfectly matching barcodes were counted. These barcodes were then transformed into gene IDs using a look-up table.

Abundances were calculated for each barcode in each sample by dividing the number of counts for that barcode by the total number of barcode counts in the sample, and any mutant with an abundance of less than 0.1% in the input sample was discarded from subsequent analysis (and abundances recalculated for remaining mutants).

To estimate sampling variation, the approximate number of parasite genomes put into the barcode PCR was calculated using the typical parasitemia per day of experiment, an estimate of the efficiency of DNA extraction, and the proportion of the extracted DNA used as template. The variance at this first stage was calculated for each mutant using the binomial distribution as *np(1 − p)* where *n* is the expected number of parasite genomes in the sample and *p* the barcode-abundance seen for the mutant. This variance was increased by a factor representing the noise added by PCR, calculated by simulating 45 cycles of PCR repeatedly on a range of input concentrations and observing the resulting distribution. It was further increased by considering a second binomial distribution representing the loading of barcodes on the PCR where *n* represented the total number of reads for the sample.

The result of this procedure was an estimate of the error associated with the barcode abundance for each mutant in each mouse on each day of the experiment. These error estimates were propagated to all downstream calculations as we calculated estimates for the relative growth rate of mutants. For each mutant in each mouse, on each day (except the final day), the barcode abundance on the following day was divided by the abundance on the current day to obtain an un-normalized relative growth rate. The error in this value was also calculated, using the estimation for propagation of variance in a ratio between random variables.

The same set of growth-rate controls were added to the transfection pool for each experiment to serve as controls. Four of these have wild-type growth rates and three have attenuated growth. The aggregate pre-normalization RGR of the wild-type growth controls was calculated on each day for each mouse by taking the inverse-variance weighted average of their values, and the variance in this aggregate value was calculated. Every RGR was now normalized by dividing it by the aggregate value of wild-type growing mutants in the sample from which it came. This leaves wild-type growing mutants with a normalized RGR of 1. The errors in both components were propagated to this normalized value.

Typically for each experiment there were 12 measurements of RGR – from 3 mice on each of 4 days, these were aggregated together using the inverse-variance weighted mean function. Variances were scaled by a constant factor of 3 at this point, which was found to improve the match between true experimental reproducibility and estimated confidence – likely reflecting the additional stochastic effects not modeled in the previous analysis. This gave a single value per mutant per experiment, with an associated variance (and hence a 95% confidence interval).

Where multiple values were available from separate experiments analyzing the same mutant, the multiple single fitness values were combined using the inverse-variance weighted mean.

Mutants were binned into phenotype categories based on statistical testing. P values were calculated representing the probability that the true value of the RGR was > 0.1 (not essential) or < 1 (not dispensable) or > 1 (fast). P values were adjusted and phenotypes were assigned as follows. If the RGR was not dispensable and not statistically different from essential it was considered *Essential*. If it was not essential and not significantly different from dispensable it was considered *Dispensable*. If it was significantly different both from dispensable and essential it was considered to give *Slow* or *Fast* growth depending on the RGR, and if it was not distinguishable from either it was considered that there was *Insufficient data* to call a phenotype.

A number of minor heuristics were added which were found to improve phenotype calling: 1) In combining the RGRs from all days into a single value, if the most confident result was also the minimum RGR result, its confidence was set to the same as that of the second-most confident. This avoided effects where one spurious sample could dominate the result with an erroneous value. 2) When calculating the apparent RGR of normal-growth parasites in order to normalize other results we found that, after conducting the analysis once, we could additionally use the RGR of the slow-growth controls by calibrating them to their known values. This allowed all 7 measurements to be used for calibration. 3) Where mutants which obtained an initial phenotype of *Insufficient data* were analyzed a second time, we observed a strong association with *Essential* phenotypes. We therefore elected to use an endpoint assay to classify a portion of *Insufficient data* mutants - those that had an abundance in the population on day 6 less than 0.1% of their abundance in the input (calibrated to a control gene) were considered to be *Essential*. 4). In light of an increased apparent ratio of essential phenotypes for vectors with a geometric mean homology arm length of less than 1.25 kb (Figure S3A), these vectors were excluded when calculating the overall phenotype ratio for the genome, and essential calls in this category were marked as low confidence.

#### Gene conservation analysis

Scores for inter-species conservation between *P. falciparum* and *P. chabaudi*, and of dN/dS in *P. falciparum* populations, were taken from (MalariaGEN Plasmodium falciparum Community Project, 2016). These were matched to *P. berghei* genes by orthology and binned into categories and plotted against *P. berghei* phenotype.

#### Reconstruction of gene losses and gains

The evolution of gene sets in the Apicomplexa was analyzed much as in Woo et al. (2015). We downloaded OrthoMCLDB/EuPathDB OG5 groups for all species shown on Figure 5D and in addition four outgroups (*Chromera velia*, *Vitrella brassicaformis*, *Tetrahymena thermophila* and *Chlamydomonas reinhardtii*) (Aurrecoechea et al., 2010). The presence or absence of orthologs in each species was then analyzed using Count (Csurös, 2010) to infer ancestral ortholog presence using Dollo parsimony.

#### GO term enrichment

Gene ontology terms were downloaded from GeneDB, both for *P. berghei* and for the better annotated *P. falciparum*. *P. falciparum* annotations were assumed to also apply to any 1:1 orthologous *P. berghei* genes. The *topGO* R package (Alexa et al., 2006) was used to calculate enrichment with the weight01 algorithm, which takes account of GO tree topology.

#### Expression analysis

FPKM data from Otto et al. (2014) was processed as follows: the average of multiple measurements from each stage was taken, these average absolute values were partitioned per gene into proportions of the total FPKM from each of the five life-stages. K-means clustering was applied to sort genes into 9 clusters. A measure of “sexiness” was calculated by dividing the FPKM from the sexual stages (gametocytes and ookinetes) by the total.

### Data and Software Availability

The majority of analysis was conducted in R. In the interest of reproducibility, we include a file archive (Data S1) containing an R script, along with the raw barcode counts for each experiment. This script will process and analyze the raw barcode data and generate the figures presented.

## Author Contributions

Conceptualization, O.B. and J.C.R.; Methodology, A.R.G., T.S., E.B., and L.P.; Software, F.S. and T.S.; Formal Analysis, T.S., F.S., and G.G.R.; Investigation, E.B., A.R.G., B.A., G.G., C.H., and T.M.; Writing – Original Draft, O.B.; Writing – Review & Editing, T.S., J.C.R., R.E.M., M.W.M, G.I.M.F., A.B.V., K.W., E.B., A.R.G., K.M., F.S., and L.P.; Visualization, T.S., R.E.M., M.W.M., G.I.M.F., and A.B.V.; Supervision, E.B., O.B., and J.C.R.; Project Administration, E.B. and K.M.

## Supplemental Information

Table S1. The Full Results of the Screen Including Vector IDs, RGR Measurements, Confidence Limits, and Phenotype Calls, Related to Figure 1 The latest data are also available to browse interactively or download as a customisable CSV at http://plasmogem.sanger.ac.uk

Table S2. The Data Underpinning the Comparison of Gene Properties in the Screen Compared with the Genome, Related to Figure S1

Table S3. Genes Clustered by Location or Function, Related to Figures 2, 4, 6, and S4

Table S4. *P. berghei* Genes Clustered by Expression Patterns, Related to Figure 2 Conserved Plasmodium genes of unknown function which belong to the ookinete cluster (cluster 9), but have a barseq phenotype in asexual blood stages are highlighted and may be filtered in column L. The expression data were from RNA sequencing experiments reported by Otto et al. (2014).

Table S5. Two-Way and Three-Way Orthologs for *P. berghei*, *T. gondii*, and *P. falciparum* and Available Phenotype Data, Related to Figure 3

Table S6. Phases at which *P. berghei* Genes Were Gained, with Phenotyping Data, Related to Figure 5 This includes genes which are required for normal asexual blood stage growth and that belong to groups of orthologs acquired by the ancestral hematozoan, suggesting they may be important for the intraerythrocytic lifestyle of malaria parasites.

Table S7. All GO Enrichment Data Including which Genes Are Responsible for Enrichment, Related to Figures 6 and S4

Table S8. Summary of the Phenotyping Data, Gene Identifiers, and Known or Putative Functions of the Transporters Shown in Figure 6B, Related to Figure 6

Data S1. Reproducible Analysis Procedure The file archive includes an R-markdown document, which will reproduce all bioinformatic procedures conducted.
