# Supplementary material for: Functional Profiling of a Plasmodium Genome Reveals an Abundance of Essential Genes
Source: Cell. 2017 Jul 13;170(2):260–272.e8. doi: 10.1016/j.cell.2017.06.030 (PMC5509546; doi:10.1016/j.cell.2017.06.030)
Supplement: Data S1. Reproducible Analysis Procedure — The file archive includes an R-markdown document, which will reproduce all bioinformatic procedures conducted. [file mmc9.zip › ReproducibleAnalysis.pdf]

Reproducible analysis procedure

Preliminary stages

Dependencies

Load required packages

```
library(scales)
library(plyr)
library(dplyr)
library(Hmisc)
library(ggplot2)
library(reshape2)
library(gridExtra)
library(topGO)
```

First we include a script containing a helper function, loadSTM() which for each experiment, performs the initial processing required to estimate RGR and its variance for each included mutant. The procedure involved is described in the Methods section.

```
source("PerExperimentAnalysis.R")
```

Definitions

We here define phenotype color scheme and factor order

```
phenolevels = c("Insufficient data", "Essential", "Slow", "Dispensable", "Fast",
    "Unselected")

phenolevelscolor = c("black", "#f90f00", "#0f3791", "#007e41", "#f83cd9", "darkgray")  #standard phenot
othercolors = c("#66c2a5", "#fc8d62", "#8da0cb", "white")  #used when another palette is needed
names(phenolevelscolor) = phenolevels
```

Analysis of each experiment separately from barcode counts

We iterate through all experiment data files calculating RGRs and variances.

```
files <- list.files(path = "./countfiles/", pattern = "*.csv", full.names = T,
    recursive = FALSE)
inputs <- lapply(files, loadSTM)
# Iterate through files loading each to make a list of dataframes
tables <- vector("list", length(inputs))
# Empty containers, table is a dataframe with main data.
for (i in 1:length(inputs)) {
    tables[[i]] <- inputs[[i]]$table

}
# Merge the many experiment dataframes into one large one:
fullSet <- ldply(tables, data.frame)
```

```
fullSet$Relative.Growth.Rate = fullSet$fitness
```

Preparing data for analysis:

Remove a small number of non-standard vectors:

```
fullSet <- fullSet[!(grepl("tag", fullSet$gene, ignore.case = TRUE)), ]
fullSet <- fullSet[!(grepl("ko", fullSet$gene, ignore.case = TRUE)), ]
```

Load supplementary data files, such as gene descriptions, ortholog IDs and the connection between a barcode and the construct used in the experiment.

```
geneinfo <- read.csv("./otherdata/geneinfo.csv")
genomiclocations <- read.csv("./otherdata/genomiclocations.csv", header = TRUE)
genomiclocations$csome = as.numeric(as.character(genomiclocations$csome))

cloneIDs <- read.table("./otherdata/homology_arm_lengths_and_cloneids.csv",
    header = TRUE)
controls <- read.table("./otherdata/controls.txt", header = TRUE)
experimentsdf <- data.frame(experiment = unique(cloneIDs$experiment), joiner = 1)
controls$joiner = 1
combined <- merge(controls, experimentsdf)  #the same controls are used in each batch so the control se
cloneIDs <- rbind.fill(cloneIDs, combined)
```

Additional helper functions:

Derivative data values:

```
addExtraData <- function(df) {
    # The key data this algorithm uses is a Relative Growth Rate and a variance.
    # But for human consumption, various derivatives are needed. This calculates
    # them. It also adds various gene-specific things. And calculates
    # 'confidence', the negative log of the variance
    df$lower = df$Relative.Growth.Rate - 2 * sqrt(df$variance)  #95% CIs
    df$upper = df$Relative.Growth.Rate + 2 * sqrt(df$variance)  #95% CIs

    comb <- merge(df, geneinfo, by.x = "gene", by.y = "Old.Gene.ID", all.x = TRUE)
    comb <- merge(comb, genomiclocations, by.x = "current_version_ID", by.y = "gene",
        all.x = TRUE)
    comb$Confidence = -log(comb$variance)
    # comb$Relative.Growth.Rate[!is.finite(comb$variance) ] =
    # sample(1:1000/1000, nrow(comb[!is.finite(comb$variance), ]),replace=TRUE)
    # #where variance is infinite supply a placeholder RGR to allow analysis to
    # proceed
    comb$Confidence[!is.finite(comb$Confidence)] = 0.1
    comb$Relative.Growth.Rate[comb$Confidence < 0.1] = NA

    comb$Confidence[comb$Confidence < 0.1] = 0.1  #floor
    comb <- mutate(comb, Confidence = ifelse(Confidence > 10, 10, Confidence))  #ceiling


    return(comb)
}
```

Function to calculate the inverse variance weighted mean and its variance. This is used to merge multiple observations of a mutant.

```
gaussianMeanAndVariance2 <- function(vals, variances) {
    # This function calculates the inverse variance weighted mean and its
    # variance
    df <- data.frame(value = vals, variance = variances)
    df <- df[complete.cases(df), ]
    vals = df$value
    variances = df$variance
    if (length(vals) == 1) {
        # If there is only one value just pass through mean as value and variance
        # unchanged
        var <- variances[1]
        mean <- vals[1]
    } else {
        precs = 1/variances
        # The weight is the precision, the inverse of the variance
        mean = sum(vals * precs)/sum(precs)
        var1 = (1/sum(precs)) * (1/(length(vals) - 1)) * sum((vals - mean)^2/variances)
        # Formula 1 for variance, more conservative in general
        var2 = 1/sum(precs)
        # Formula 2 for variance
        if (is.na(var1)) {
            var1 <- 0
        }
        var <- max(var1, var2)  #Take the max of the two variance estimates to be conservative
    }
    return(data.frame(mean = mean, var = var))
}
```

Function to assign phenotypes:

```
addPhenotypes <- function(newcomb) {

    pvalue = 0.05

    # Perform statistical test for difference to 1 (i.e. not dispensable)
    newcomb$z1 <- (1 - newcomb$Relative.Growth.Rate)/sqrt(newcomb$variance)
    newcomb$p1 <- 2 * pnorm(-abs(newcomb$z1))
    newcomb$f1 = p.adjust(newcomb$p1, method = "fdr")
    newcomb$call1 = FALSE
    newcomb[!is.na(newcomb$f1) & newcomb$f1 < 0.05, ]$call1 = TRUE
    # Perform statistical test for difference to 0.1 (i.e. not essential)
    newcomb$z0 <- (0.1 - newcomb$Relative.Growth.Rate)/sqrt(newcomb$variance)
    newcomb$p0 <- pnorm(-abs(newcomb$z0))
    newcomb$f0 = p.adjust(newcomb$p0, method = "fdr")
    newcomb$call0 = FALSE
    newcomb[!is.na(newcomb$Relative.Growth.Rate) & newcomb$f0 < pvalue & newcomb$Relative.Growth.Rate >
        0.1, ]$call0 = TRUE
    newcomb$phenotype = "None"
    newcomb$phenotype[is.finite(newcomb$variance) & newcomb$call0 & newcomb$call1 &
        newcomb$Relative.Growth.Rate > 1] <- phenolevels[5]
    # If not dispensable and RGR>1 then fast
    newcomb$phenotype[newcomb$call0 & newcomb$call1 & newcomb$Relative.Growth.Rate <
```

```
        1 & newcomb$Relative.Growth.Rate > 0.1] <- phenolevels[3]
    # If not dispensable and not essential and RGR<1 then slow
    newcomb$phenotype[newcomb$call0 & !newcomb$call1] <- phenolevels[4]
    # If not essential and could be dispensable then dispensable
    newcomb$phenotype[!newcomb$call0 & newcomb$call1] <- phenolevels[2]
    # If not dispensable and could be essential then essential
    newcomb$phenotype[!(newcomb$call0 | newcomb$call1)] <- phenolevels[1]
    # If could be essential or dispensable then insufficient data

    # The section below is a heuristic applied to 'insufficient data' vectors,
    # use an endpoint test: check the amount of the vector on day 7 compared to
    # the amount in the input. If this is less than 1% of the control vector
    # then consider the vector essential but with very low confidence.

    extraessentials = (newcomb$phenotype == phenolevels[1] & (is.na(newcomb$normd7toinputA) |
        newcomb$normd7toinputA < 0.01))
    newcomb$type = ifelse(extraessentials, "extra", "normal")
    newcomb$Confidence = ifelse(extraessentials, 1, newcomb$Confidence)
    newcomb$Relative.Growth.Rate = ifelse(extraessentials, 0.1, newcomb$Relative.Growth.Rate)
    newcomb$phenotype = ifelse(extraessentials, phenolevels[2], newcomb$phenotype)


    # There is a possible bias towards essential genes (see later figure) in the
    # small number of vectors with geometric mean homology arm length below 1250
    # bp, we therefore flag these
    newcomb$type = ifelse(sqrt(newcomb$left_arm_length * newcomb$right_arm_length) <
        1250 & newcomb$phenotype == "Essential", "shortarm", newcomb$type)
    newcomb$phenotype = factor(as.character(newcomb$phenotype), levels = phenolevels)
    return(newcomb)
}
```

Merge multiple observations of each mutant

```
fullSet <- addExtraData(fullSet)
fullSet <- merge(fullSet, cloneIDs, by = c("gene", "experiment"), all.x = TRUE)

# Exclude genes which were not meant to be in the experiment in which they
# were read, and may represent contamination
fullSet <- fullSet[!is.na(fullSet$cloneid), ]

singlecomb <- fullSet
# This retrieves a version of the dataset with one row per gene per
# experiment, in contrast to that below which combines multiple observations
# of the same gene
```

Define a function to merge multiple experiments:

```
mergeExperiments <- function() {
    # Take the version with one row per gene and aggregate using the
    # inverse-variance weighted mean

    fullSet2 <- singlecomb
    if (is.null(fullSet2)) {
```

```
        return(NULL)
    }
    fullSet3 <- fullSet2 %>% group_by(gene) %>% do(gaussianMeanAndVariance2(.$Relative.Growth.Rate,
        .$variance)) %>% transmute(Relative.Growth.Rate = mean, variance = var)
    fullSet4 <- fullSet2 %>% group_by(gene) %>% summarise(cloneid = paste(unique(cloneid),
        sep = ",", collapse = ","), experiments = paste(unique(experiment),
        sep = ",", collapse = ","), timesAnalysed = length(Relative.Growth.Rate),
        normd7toinputA = mean(normd7toinputA, na.rm = T), normd6toinputA = mean(normd6toinputA,
            na.rm = T), normd6toinputB = mean(normd6toinputB, na.rm = T), normd6toinputC = mean(normd6t
            na.rm = T), left_arm_length = mean(left_arm_length, na.rm = T),
        right_arm_length = mean(right_arm_length, na.rm = T))
    fullSet3$gene = as.character(fullSet3$gene)
    fullSet4$gene = as.character(fullSet4$gene)
    fullSet2 <- merge(fullSet3, fullSet4, by = c("gene"))
    fullSet2 <- addExtraData(fullSet2)
    return(fullSet2)
}
```

Results

Overall screen statistics

```
multicomb <- mergeExperiments()
main <- addPhenotypes(multicomb)
HowManyGenesAssayed = length(main$gene)
HowManyGenesAssayed
```

```
## [1] 2578
```

```
InHowManyPools = length(unique(singlecomb$experiment))
InHowManyPools
```

```
## [1] 58
```

Confidence volcano-like plot showing RGR and associated error

```
data <- addPhenotypes(multicomb)

data <- filter(data, type == "normal")
data <- filter(data, is.finite(variance))
ggplot(data, aes(x = Relative.Growth.Rate, y = Confidence, color = phenotype)) +
    geom_point(size = 0.1, alpha = 0.5) + scale_color_manual(values = phenolevelscolor) +
    labs(color = "Growth phenotype", x = "Relative growth rate") + theme_classic() +
    theme(axis.line.x = element_line(color = "black", size = 0.5), axis.line.y = element_line(color = "
        size = 0.5)) + guides(colour = guide_legend(override.aes = list(size = 2,
    alpha = 1)))
```

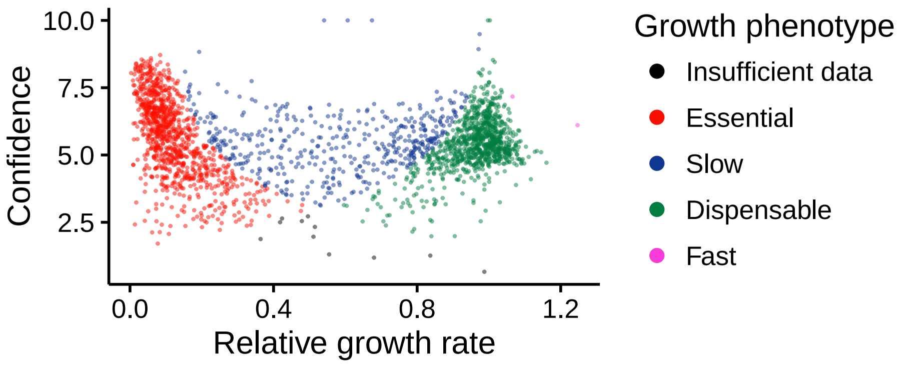


```
ggsave("output/scatterplot.pdf", width = 5, height = 2)
```

Distribution of replicates of control vectors

Since the control vectors have been transfected >50 times we can observe what distribution their RGR's create. *[There is some code not shown here which hardcodes offset phenotype colours and gene IDs for distribution presentation]*

```
starter <- addPhenotypes(singlecomb)
starter <- filter(starter, phenotype != "Insufficient data")
```

```
ggplot(starter, aes(x = Relative.Growth.Rate, fill = PrettyNames, color = PrettyNames)) +
    geom_density(alpha = 0.5, weight = 0.3, size = 0.3, bw = "SJ") + theme_classic() +
    theme(legend.position = "none") + labs(x = "Relative growth rate", y = "Rel. frequency") +
    theme(axis.line.x = element_line(color = "black", size = 0.5), axis.line.y = element_line(color = "
        size = 0.5)) + scale_x_continuous(expand = c(0, 0), breaks = c(0.25,
    0.5, 0.75, 1)) + scale_y_continuous(expand = c(0, 0), breaks = c(0, 10,
    20)) + scale_fill_manual(values = custpalette) + scale_color_manual(values = custpalette) +
    geom_text(data = annotatedf, aes(x = x, y = y, color = color, label = text,
        fill = NA), parse = T, size = 3.5) + annotate(geom = "text", lineheight = 0.9,
    hjust = 0.2, label = "Essential\nribosomal genes", x = x[1], y = y[1], size = 3.5,
    color = essentialcolor)
```

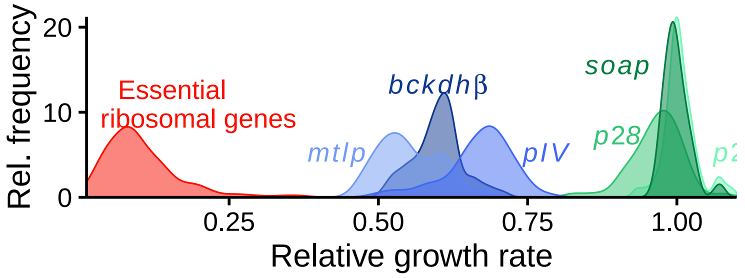


```
ggsave("output/distrplot.pdf", width = 4, height = 1.5)
```

Assessing link between calculated confidence and actual experimental reproducibility

Here we assess the link between the calculated theoretical confidence of a measurement and its true experimental reproducibility.

```
starter <- addPhenotypes(singlecomb)
maxconf <- group_by(singlecomb, gene) %>% summarise(maxconf = max(Confidence))

# starter<-filter(starter,phenotype!='Insufficient data')

starter <- starter[sample(nrow(starter)), ]  #scramble rows to avoid any effect of experiment order
cutoffs = 0:8
plotList <- vector("list", 9)
storedata = data.frame(ConfidenceGreaterThan = cutoffs, Rsquared = rep(NA, 9),
    NumberOfObservations = rep(NA, 9), NumberOfGenes = rep(NA, 9))

for (cutoff in cutoffs) {

    cs <- starter
    cs <- cs[cs$Confidence >= cutoff, ]
```

```
    table <- as.data.frame(table(cs$gene))
    multiples <- unique(table[table$Freq > 1, ]$Var1)
    tempcs <- cs
    matches <- match(multiples, cs$gene)
    tempcs[matches, ]$gene = NA
    matches <- match(multiples, cs$gene)
    matches2 <- match(multiples, tempcs$gene)
    df1 <- cs[matches, ]
    df2 <- cs[matches2, ]
    corr <- cor(df1$Relative.Growth.Rate, df2$Relative.Growth.Rate, use = "complete.obs")
    a <- ggplot(df1, aes(x = df1$Relative.Growth.Rate, y = df2$Relative.Growth.Rate,
        label = df1$gene)) + geom_point(alpha = 1, size = 0.1) + ggtitle(paste(c("Conf>",
        cutoff, ", R^2: ", round(corr^2, 2)), collapse = "", sep = "")) + geom_abline(intercept = 0,
        alpha = 0.2) + coord_cartesian(xlim = c(0, 1.2), ylim = c(0, 1.2)) +
        labs(x = "RGR 1", y = "RGR 2") + theme_bw() + scale_x_continuous(breaks = c(0,
        0.5, 1)) + scale_y_continuous(breaks = c(0, 0.5, 1)) + theme(legend.position = "none")

    print(a)
    ggsave(paste0("output/conf", cutoff, ".pdf"), a, width = 2, height = 2)

    storedata[storedata$ConfidenceGreaterThan == cutoff, "Rsquared"] = corr^2
    storedata[storedata$ConfidenceGreaterThan == cutoff, "NumberOfObservations"] = sum(starter$Confiden[illegible]
        cutoff)
    storedata[storedata$ConfidenceGreaterThan == cutoff, "NumberOfGenes"] = sum(maxconf$maxconf >=
        cutoff)
}
```

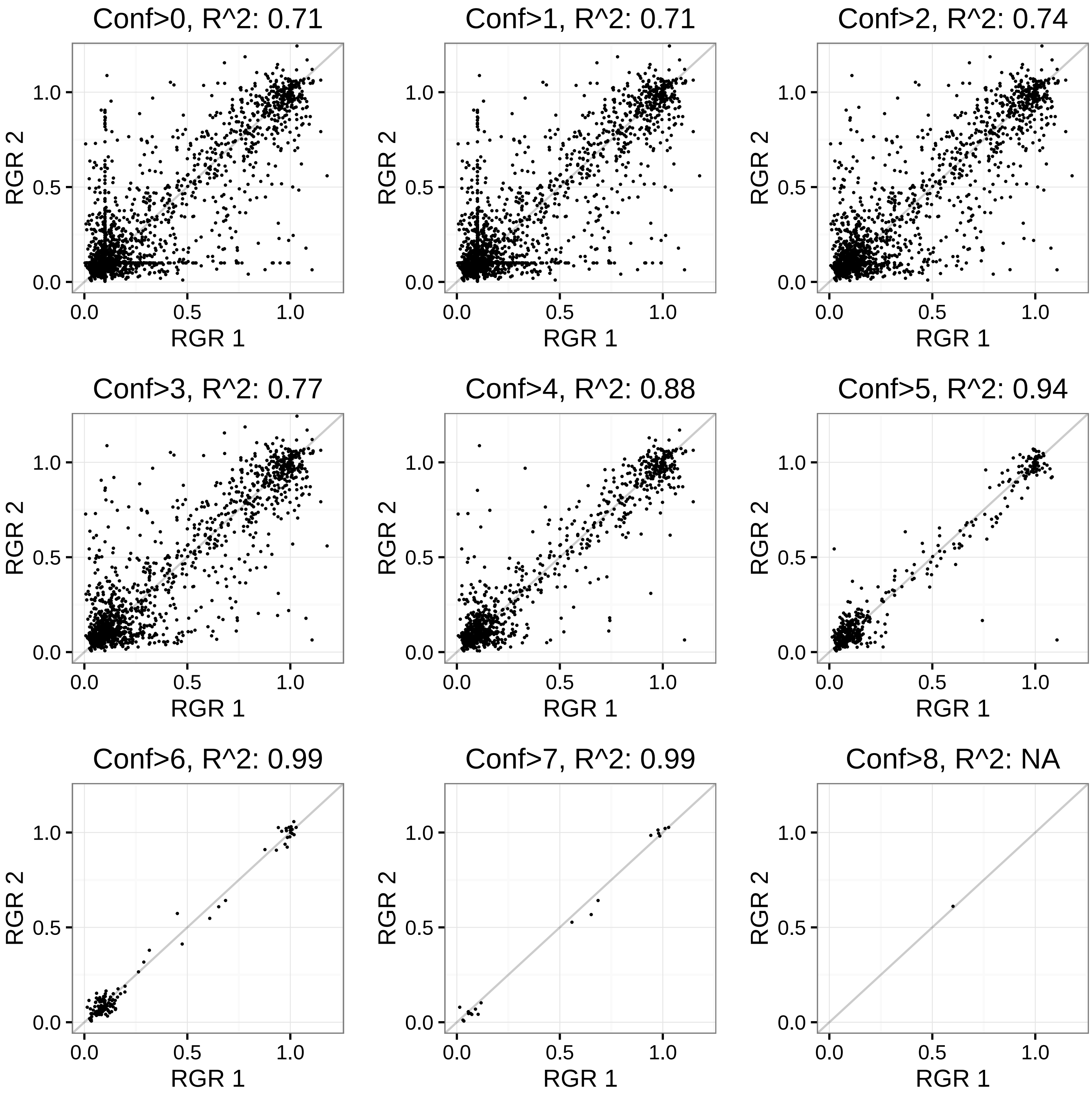
Conf>0, R^2: 0.71
Conf>1, R^2: 0.71
Conf>2, R^2: 0.74
Conf>3, R^2: 0.77
Conf>4, R^2: 0.88
Conf>5, R^2: 0.94
Conf>6, R^2: 0.99
Conf>7, R^2: 0.99
Conf>8, R^2: NA
RGR 1
RGR 2
0.0
0.5
1.0

Number of observations at each confidence level and experimental reproducibility:

```
print(storedata)
```

```
##   ConfidenceGreaterThan  Rsquared NumberOfObservations NumberOfGenes
## 1                     0 0.7055775                 5068          2578
## 2                     1 0.7098776                 5062          2503
## 3                     2 0.7350483                 4391          2480
## 4                     3 0.7686770                 4175          2407
## 5                     4 0.8770879                 3477          2190
## 6                     5 0.9395596                 2341          1597
## 7                     6 0.9870316                 1072           756
## 8                     7 0.9938522                  320           246
## 9                     8        NA                   50            49
```

```
write.csv(storedata, "storedata.csv")
```

Aggregating multiple experiments

We here visualise the effect of aggregating observations from multiple experiments, which significantly increases confidence on a per-gene basis:

```
df1 <- data.frame(type = "Before aggregation", Confidence = singlecomb$Confidence)
df2 <- data.frame(type = "After aggregation", Confidence = multicomb$Confidence)
ggplot(rbind(df1, df2), aes(x = Confidence, fill = type, color = type)) + geom_density(alpha = 0.3) +
    theme_classic() + labs(y = "Distribution", fill = "", color = "")
```

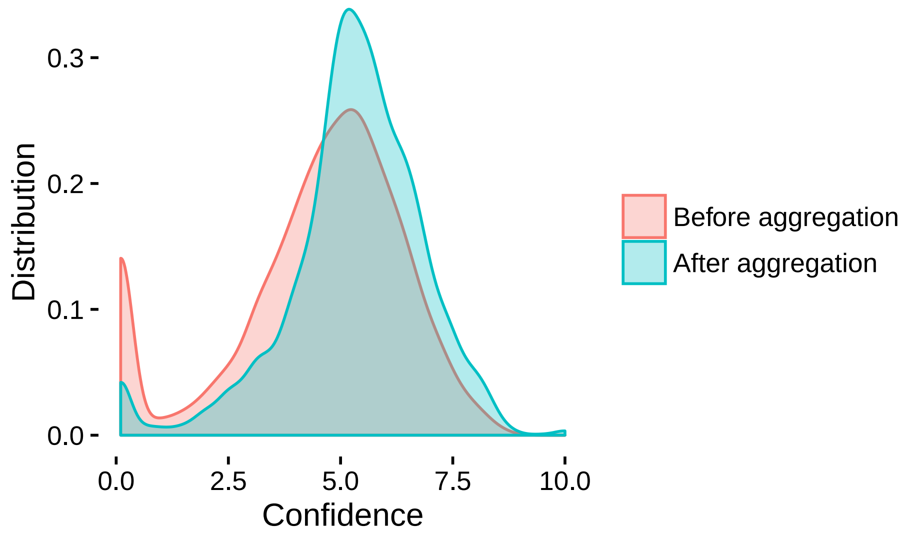

Illustrative examples of likelihood distributions and the associated phenotype

The model assumes normally distributed RGR values with a specific fitness. This panel simulates them for visualisation.

```
genes <- c("PBANKA_092520", "PBANKA_110650", "PBANKA_021550", "PBANKA_143750")
xoffset = c(0.3, 0, 0, 0)
df <- addPhenotypes(multicomb)
```

```r
df <- df[match(genes, df$gene), ]
p <- ggplot(df, aes(x = Relative.Growth.Rate))
comb = NULL
df$group = 1:nrow(df)
for (i in 1:nrow(df)) {
    newdf = data.frame(x = seq(-1, 1.5, length = 500))
    newdf$y = dnorm(newdf$x, df[i, ]$Relative.Growth.Rate, sqrt(df[i, ]$variance))
    newdf$y = newdf$y/max(newdf$y)
    newdf$phenotype = df[i, ]$phenotype
    newdf$group = i
    comb <- rbind(comb, newdf)

}
ggplot(comb, aes(x = x, y = y, color = phenotype, group = group)) + theme_bw() +
    geom_line() + facet_grid(group ~ ., scales = "free_y") + scale_color_manual(values = phenolevelscol
    geom_text(color = "black", data = df, aes(label = gene, x = xoffset), y = 0.9,
        hjust = 0) + geom_text(color = "black", data = df, aes(label = gene_product,
    x = xoffset), y = 0.6, hjust = 0) + coord_cartesian(xlim = c(0, 1.25), ylim = c(0,
    1.05)) + labs(x = "Relative Growth Rate", y = "Likelihood") + theme(axis.ticks.y = element_blank())
    scale_x_continuous(breaks = c(0, 0.5, 1)) + scale_y_continuous(breaks = NULL) +
    theme(strip.background = element_blank(), strip.text.y = element_blank()) +
    guides(color = F)
```

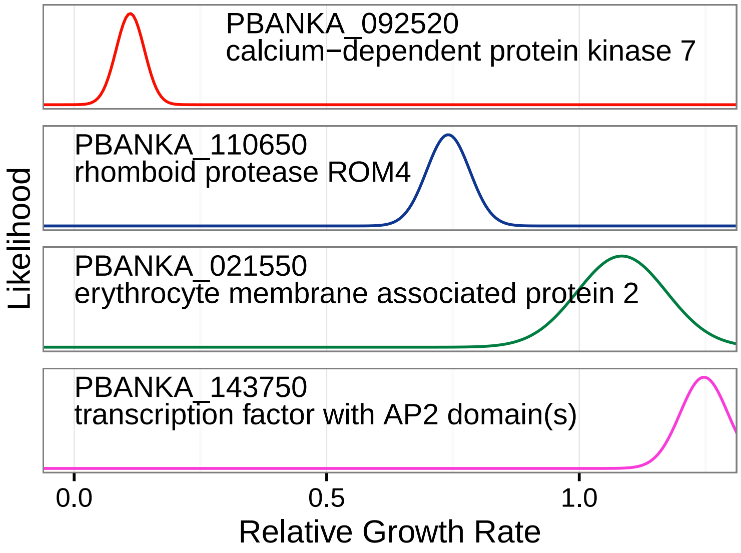


```r
ggsave("output/likelihood.pdf", width = 4, height = 3)
```

Overall phenotype distribution

Here we display the phenotype distribution both in terms of raw numbers, and after excluding vectors with a geometric mean homology arm length < 1.25 kb in case of technical artefacts.

```
multi <- addPhenotypes(multicomb)

multi <- multi[multi$phenotype != "Insufficient data", ]

nrow(multi)
```

```
## [1] 2564
```

```
df <- as.data.frame(table(multi$phenotype))
df[df$Freq > 0, ]
```

```
##          Var1 Freq
## 2   Essential 1195
## 3        Slow  456
## 4 Dispensable  911
## 5        Fast    2
```

Exclude any vector with geometric mean homology length less than 1250 bp for this analysis to avoid a potential bias for low homology arm lengths which will be seen later

```
multi <- filter(multi, sqrt(left_arm_length * right_arm_length) > 1250)

nrow(multi)
```

```
## [1] 2364
```

```
df <- as.data.frame(table(multi$phenotype))
df <- df[df$Freq > 0, ]

sum <- sum(df$Freq)
df$prop = df$Freq/sum
df
```

```
##          Var1 Freq         prop
## 2   Essential 1060 0.4483925550
## 3        Slow  425 0.1797800338
## 4 Dispensable  877 0.3709813875
## 5        Fast    2 0.0008460237
```

```
ggplot(df, aes(x = 1, fill = Var1, y = Freq)) + geom_bar(stat = "identity",
    color = "black") + scale_fill_manual(values = phenolevelscolor) + blank_theme +
    coord_polar("y", direction = 1) + theme(axis.text.x = element_blank()) +
    geom_text(aes(y = Freq/3 + c(0, cumsum(Freq)[-length(Freq)]), label = percent(Freq/sum(Freq))),
        size = 3) + theme(axis.text.y = element_blank()) + labs(fill = "Phenotype")
```

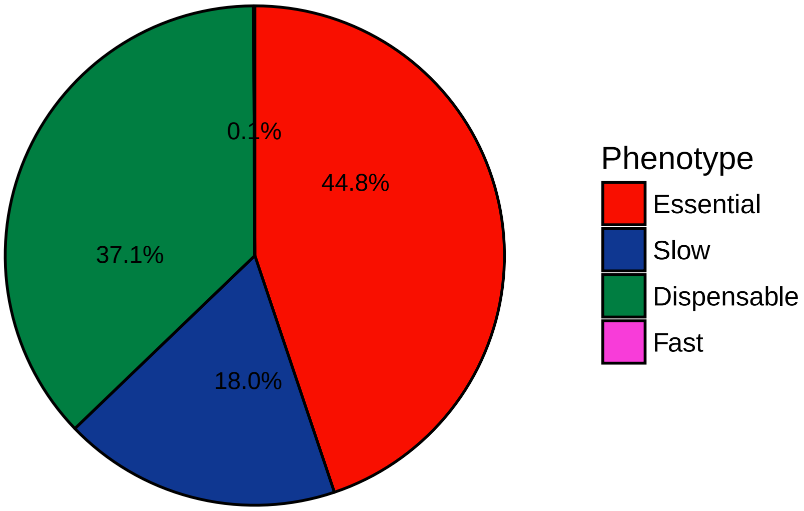
0.1%
44.8%
37.1%
18.0%
Phenotype
Essential
Slow
Dispensable
Fast

Comparison with RMgmDB

In this section we compare PlasmoGEM screening results to those recorded in the RMgmDB database..

In this fullest version we separately include genes successfully targeted on RMgmDB which revealed an asexual phenotype of some description. Reassuringly these are enriched for slow growth phenotypes in our screen.

```r
rmgm <- read.table("./otherdata/rmgmdb.txt", sep = "\t", header = T, stringsAsFactors = F)

cs <- addPhenotypes(multicomb)

m <- merge(cs, rmgm, by.x = "gene", by.y = "gene")


m <- m[m$phenotype != "Insufficient data", ]

ggplot(m, aes(x = phenotype, fill = phenotype)) + geom_bar() + facet_wrap(~RMGMpheno) +
    scale_fill_manual(values = phenolevelscolor) + theme_bw() + labs(y = "Number of genes",
    fill = "PlasmoGEM phenotype", x = "") + theme(axis.text.x = element_text(angle = 90,
    hjust = 1))
```

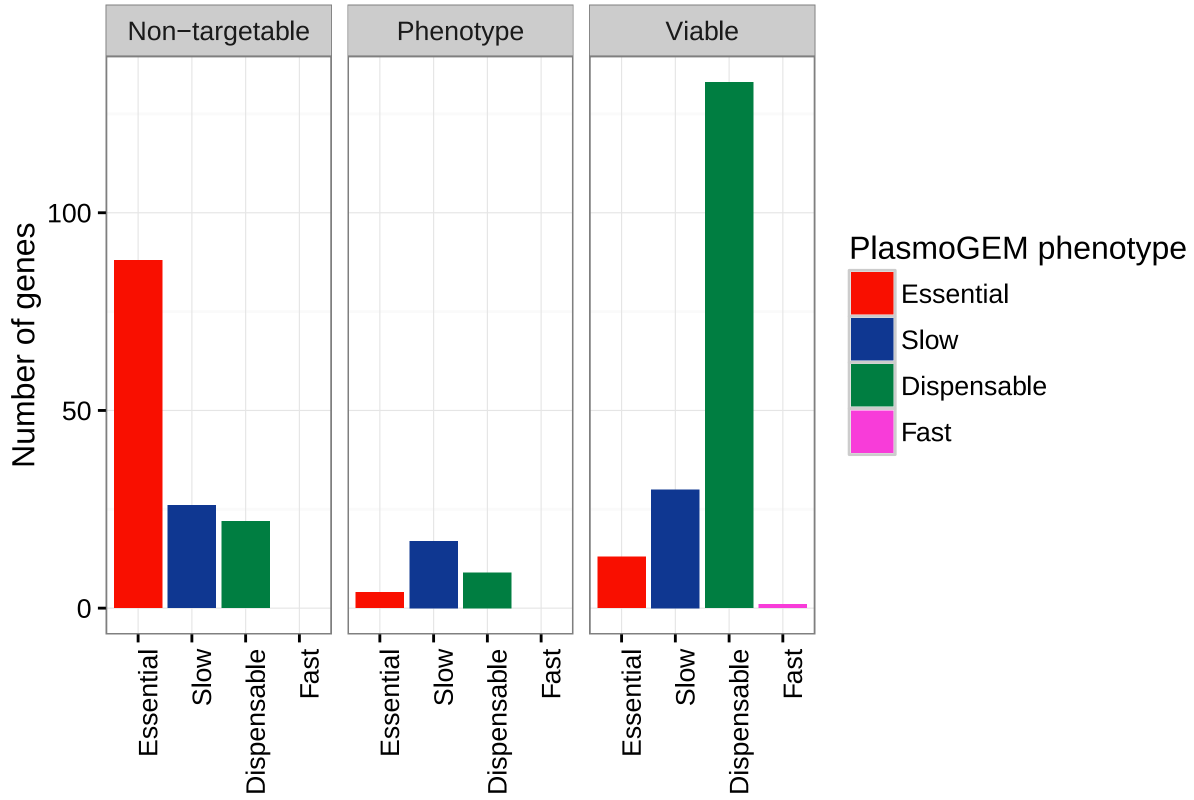

We now plot a simpler version where we simplify the RMgmDB phenotype to targetable or non-targetable.

```r
m$phenotype2 = as.character(m$phenotype)
m[m$RMGMpheno == "Phenotype", ]$RMGMpheno = "Viable"
m[m$phenotype == "Slow", "phenotype2"] = "Viable"
m[m$phenotype == "Fast", ]$phenotype2 = "Viable"
```

```r
m[m$phenotype == "Dispensable", ]$phenotype2 = "Viable"
m[m$phenotype == "Essential", ]$phenotype2 = "Non-targetable"
m$RMGMpheno2 = paste0("RMgmDB:", m$RMGMpheno)
```

```r
ggplot(m, aes(x = phenotype2, fill = phenotype)) + geom_bar(width = 0.5, color = "black",
    size = 0.2) + facet_wrap(~RMGMpheno2) + scale_fill_manual(values = phenolevelscolor) +
    labs(x = "Barseq viability", fill = "Barseq phenotype", y = "Number of genes") +
    theme_classic(base_size = 10) + scale_y_continuous(expand = c(0, 0), limits = c(0,
    200)) + theme(axis.line.x = element_line(color = "black", size = 0.5), axis.line.y = element_line(c
    size = 0.5))
```

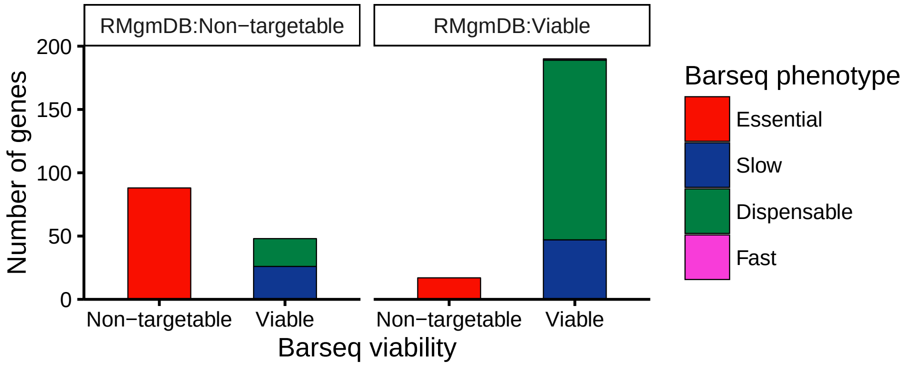

Figure 1: 1A

```r
table(m$phenotype, m$RMGMpheno)
```

```
##
##                     Non-targetable Viable
##   Insufficient data              0      0
##   Essential                     88     17
##   Slow                          26     47
##   Dispensable                   22    142
##   Fast                           0      1
##   Unselected                     0      0
```

Functional groupings

In this section we will draw various plots of phenotypes grouped by various organellar or functional categories. First we define a helper function to draw violin plots with pie charts, the code is omitted in the PDF output.

Expected essentials

Are ribosomal proteins and proteins associated with drug resistance essential as one would predict?

```r
comb <- addPhenotypes(multicomb)
comb <- comb[comb$phenotype != "Insufficient data", ]
categories1 <- read.csv("otherdata/categories1.csv", header = T)


adddata <- merge(categories1, comb)
```

```r
writeTable(adddata, "maincategories")
grid.newpage()
pushViewport(viewport(angle = -90, width = unit(1.5, "inches"), height = unit(7,
    "inches")))
grid.draw(plotViolins(filter(adddata, category %in% c("Ribosomal", "Drug-associated"))))
```

```
## [1] "Drug-associated  enriched in  Essential , p =  0.0604354693864307"
## [1] "Ribosomal  enriched in  Essential , p =  2.09079111056293e-15"
```

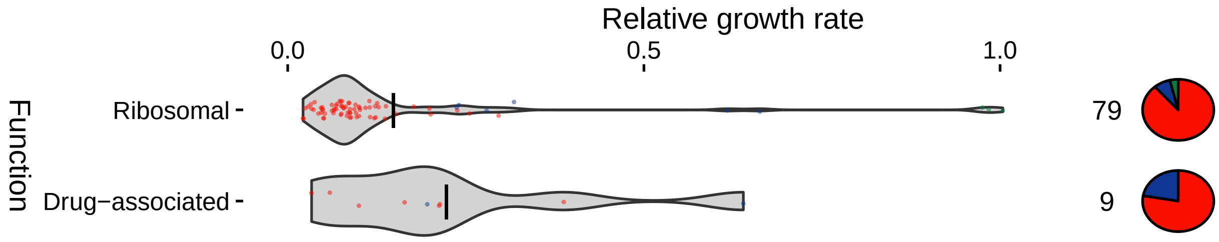

Conserved unknowns

Are *conserved Plasmodium protein unknown function* proteins, which make up much of the genome, still important?

```r
unknownfunction <- read.csv("otherdata/unknownfunction.csv", header = T, stringsAsFactors = F)
annotatedvsnot <- merge(unknownfunction, comb, all.y = T)
annotatedvsnot$category = ifelse(is.na(annotatedvsnot$category), "Annotated",
    annotatedvsnot$category)
grid.newpage()
pushViewport(viewport(angle = -90, width = unit(1.5, "inches"), height = unit(7,
    "inches")))
grid.draw(plotViolins(annotatedvsnot))
```

```
## [1] "Annotated  enriched in  Essential , p =  9.49631907835612e-05"
## [1] "Annotated  enriched in  Slow , p =  0.00128036764543094"
## [1] "unknownfunction  enriched in  Dispensable , p =  1.27160524069976e-10"
```

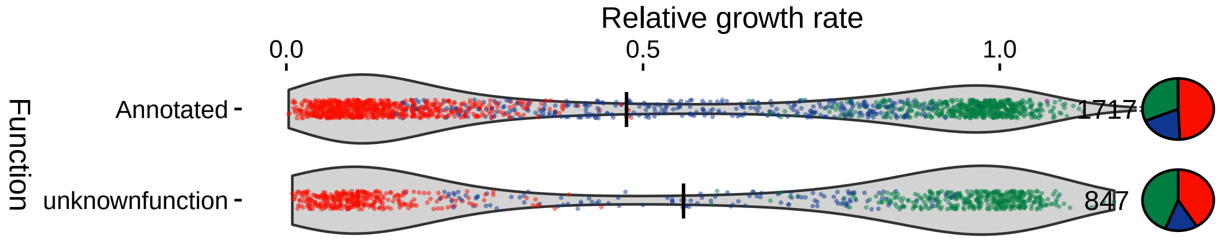

Merozoite

We assess the phenotype distributions of curated lists of merozoite genes grouped by protein localisation.

```r
grid.newpage()
pushViewport(viewport(angle = -90, width = unit(1.25, "inches"), height = unit(4,
    "inches")))
```

```
grid.draw(plotViolins(filter(adddata, category %in% c("Merozoite surface", "Rhoptry",
    "Microneme")), "merozoite"))
```

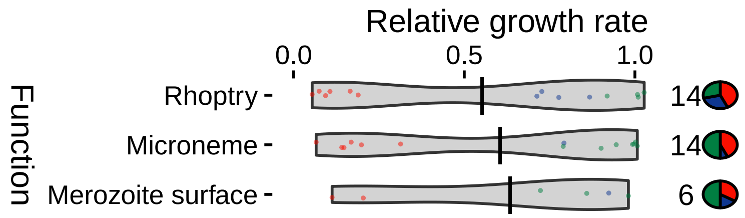

Protein export

We here assess the phenotype distributions of curated lists of exported and 'exporting' genes grouped into categories.

```
grid.newpage()
pushViewport(viewport(angle = -90, width = unit(1.5, "inches"), height = unit(4,
    "inches")))
grid.draw(plotViolins(filter(adddata, category %in% c("Bir", "Fam", "Other exported",
    "PTEX")), "export"))
```

```
## [1] "Bir  enriched in  Dispensable , p =  0.0138027915734814"
## [1] "Fam  enriched in  Dispensable , p =  0.00153713246283645"
## [1] "Other exported  enriched in  Dispensable , p =  5.78506532742858e-05"
```

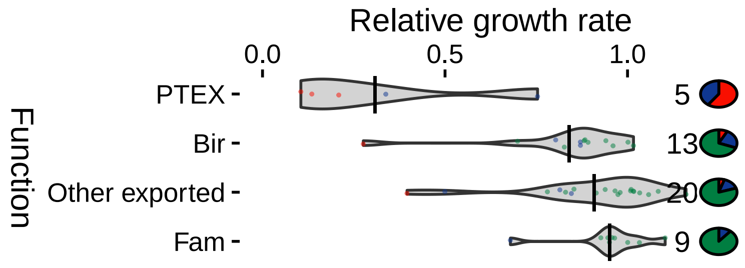

Conservation

This section uses dN/dS and inter-species conservation from Miotto *et al.* to investigate the relationship between selection pressures and phenotype distribution.

```
data<-read.table("otherdata/MiottoEtAlConservation.txt")
colnames(data)=c("PfID","Conservation.Score", "nafr","nafrR","nsea","nseaR","safr","safrR","ssea","sseaR
data$dnds=data$nafr/data$safr;
cs<-addPhenotypes(multicomb)
m<-merge(data,cs,all.y=T)
m<-m[m$phenotype %in% c("Essential","Slow","Dispensable"),]
a<-ggplot(m,aes(x=rank(-Conservation.Score),y=..count..,fill=phenotype))+stat_density(position="fill")+

df1 <- data.frame(a = c(1, 1:2,2), b = c(2.8,3,  3, 2.8))
df2 <- data.frame(a = c(2, 2:3,3), b = c(2.3,2.5,  2.5, 2.3))

b<-ggplot(m,aes(x=phenotype,y=Conservation.Score,fill=phenotype))+ coord_cartesian(ylim=c(0,3.3))+scale_
geom_line(data = df1, aes(x = a, y = b,fill=NA))+ annotate("text", x = 1.5, y = 3.05, label = "***", si
geom_line(data = df2, aes(x = a, y = b,fill=NA))+ annotate("text", x = 2.5, y = 2.55, label = "***", si
theme(axis.line.x = element_line(color="black", size = 0.5),
axis.line.y = element_line(color="black", size = 0.5))+guides(fill=FALSE) +scale_y_continuous(expand=c(



model<-aov(Conservation.Score~phenotype,data=m)
summary(model)
```

```
##               Df Sum Sq Mean Sq F value Pr(>F)
## phenotype      2    179   89.32    43.3 <2e-16 ***
## Residuals   2415   4981    2.06
## ---
## Signif. codes:  0 '***' 0.001 '**' 0.01 '*' 0.05 '.' 0.1 ' ' 1
## 144 observations deleted due to missingness
```

```
TukeyHSD(model)
```

```
##   Tukey multiple comparisons of means
##     95% family-wise confidence level
##
## Fit: aov(formula = Conservation.Score ~ phenotype, data = m)
##
## $phenotype
##                              diff        lwr         upr     p adj
## Slow-Essential         -0.3296066 -0.5204046 -0.13880850 0.0001553
## Dispensable-Essential -0.6040727 -0.7572475 -0.45089794 0.0000000
## Dispensable-Slow      -0.2744661 -0.4755677 -0.07336453 0.0039676
```

```
m$dndsbin<-ifelse(is.na(m$dnds),"NC", ifelse(m$dnds>1,">1", "<1"))
d2 <- m %>%
group_by(dndsbin,phenotype) %>%
summarise(count=n()) %>%
mutate(perc=count/sum(count))
plotc<- ggplot(d2,aes(x=dndsbin,fill=phenotype,y=perc))+geom_bar(position=position_dodge(width=.8),stat
axis.line.y = element_line(color="black", size = 0.5)) +scale_y_continuous(expand=c(0,0),labels = scale
grid.arrange(a,b,plotc , ncol=3, nrow = 1,
```

```
widths = c(1.8, 2.7,2.7))
```

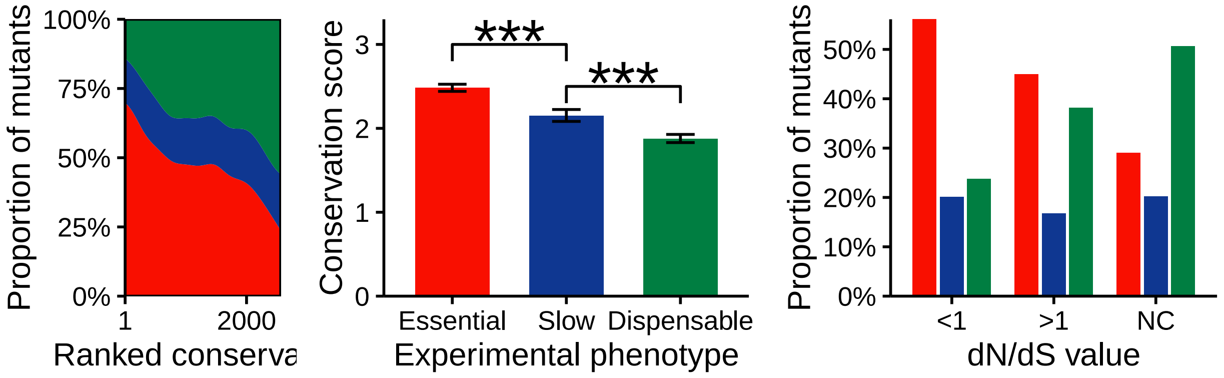

Mitochondrion

We here assess the phenotype distributions of curated lists of mitochondrial genes grouped into pathways.

```
comb <- addPhenotypes(multicomb)
comb <- comb[comb$phenotype != "Insufficient data", ]
mitochondria <- read.csv("otherdata/Mitochondria.csv", header = T)
merge <- merge(comb, mitochondria)

grid.newpage()
pushViewport(viewport(angle = -90, width = unit(7, "inches"), height = unit(6,
    "inches")))
subset <- merge[!(merge$category %in% c("nucleobase and nucleotide metabolism",
    "heme biosynthesis", "NADH/NADPH metabolism")), ]
writeTable(subset, "mitochondria")
grid.draw(plotViolins(subset, "mitochondria"))
```

```
## [1] "mETC & assembly  enriched in  Essential , p =  0.000823165547329775"
## [1] "ubiquinone-dependent oxidoreductases  enriched in  Slow , p =  0.0733453071075358"
## [1] "iron-sulfur cluster biogenesis  enriched in  Essential , p =  0.0544616442607017"
## [1] "mitochondrial protein import  enriched in  Essential , p =  0.0343173763077588"
## [1] "mitochondrial translation  enriched in  Essential , p =  9.97436529699247e-05"
## [1] "ATP synthase complex and assembly  enriched in  Slow , p =  0.000174732396246323"
## [1] "TCA and BCKDH  enriched in  Slow , p =  0.00187219980746497"
```

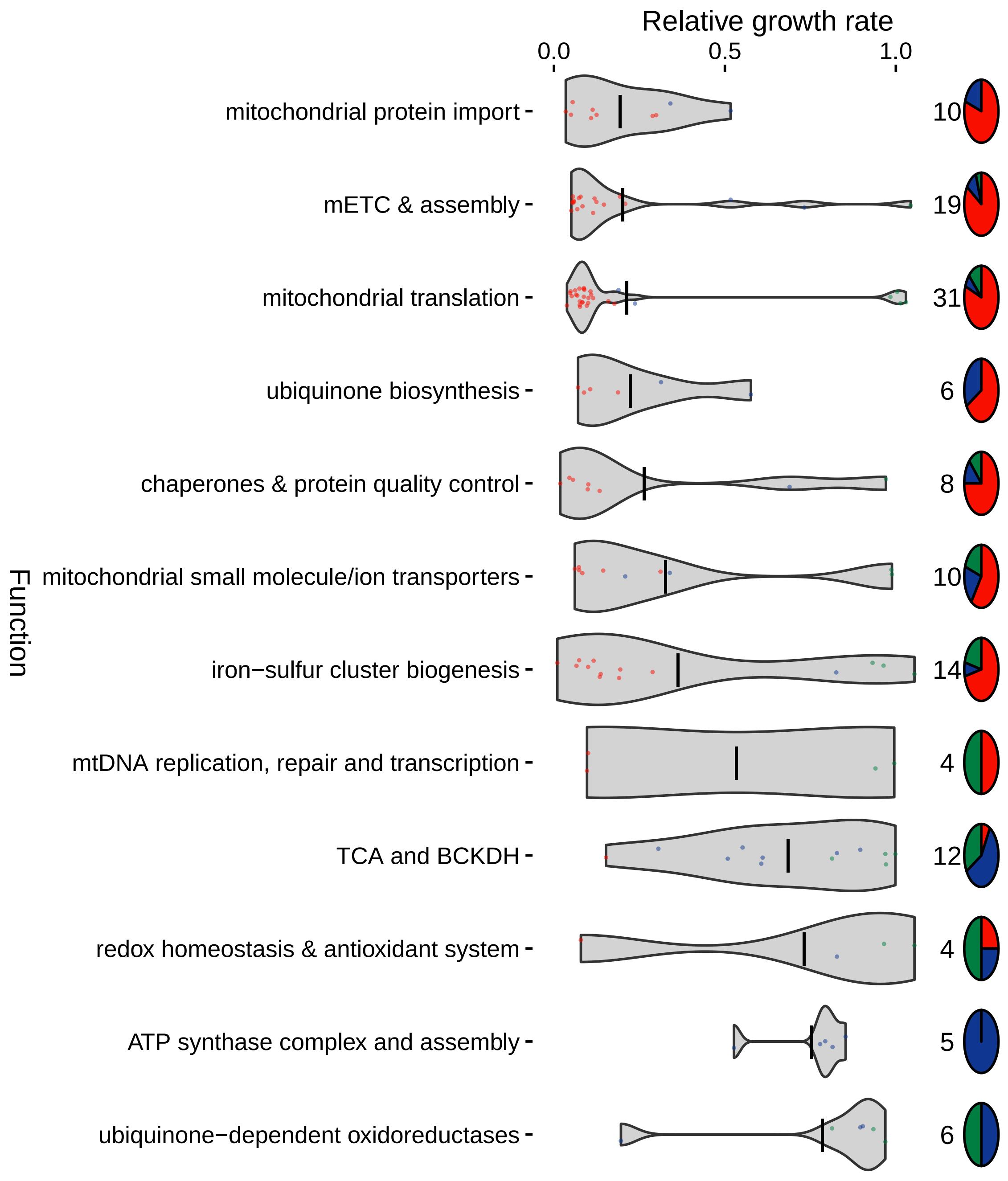
Relative growth rate
0.0
0.5
1.0
Function
mitochondrial protein import
mETC & assembly
mitochondrial translation
ubiquinone biosynthesis
chaperones & protein quality control
mitochondrial small molecule/ion transporters
iron−sulfur cluster biogenesis
mtDNA replication, repair and transcription
TCA and BCKDH
redox homeostasis & antioxidant system
ATP synthase complex and assembly
ubiquinone−dependent oxidoreductases
10
19
31
6
8
10
14
4
12
4
5
6

Apicoplast

We here assess the phenotype distributions of curated lists of apicoplast genes grouped into pathways.

```
comb <- addPhenotypes(multicomb)
comb <- comb[comb$phenotype != "Insufficient data", ]
apicoplast <- read.table("otherdata/Apicoplast.txt", header = T, sep = "\t")
apicoplast$category = as.character(apicoplast$category)
apicoplast[apicoplast$category == "apicoplast DNA replication, repair & transcription",
    ]$category = "apicoplast DNA.."
apicoplast[apicoplast$category == "chaperones & protein quality control & post-translational modificati
    ]$category = "protein folding & QC"
merge <- merge(comb, apicoplast)

grid.newpage()
pushViewport(viewport(angle = -90, width = unit(5.15, "inches"), height = unit(5,
    "inches")))
grid.draw(plotViolins(merge, "apicoplast"))
```

```
## [1] "protein translation  enriched in  Essential , p =  0.000738302368182143"
## [1] "heme biosynthesis  enriched in  Dispensable , p =  0.0568562550822029"
## [1] "isopentenyl diphosphate synthesis  enriched in  Essential , p =  0.0428724079457538"
## [1] "apicoplast DNA..  enriched in  Essential , p =  0.0627188550193605"
## [1] "fatty acid biosynthesis  enriched in  Dispensable , p =  0.0726492081425276"
## [1] "protein folding & QC  enriched in  Essential , p =  0.00493676357788202"
```

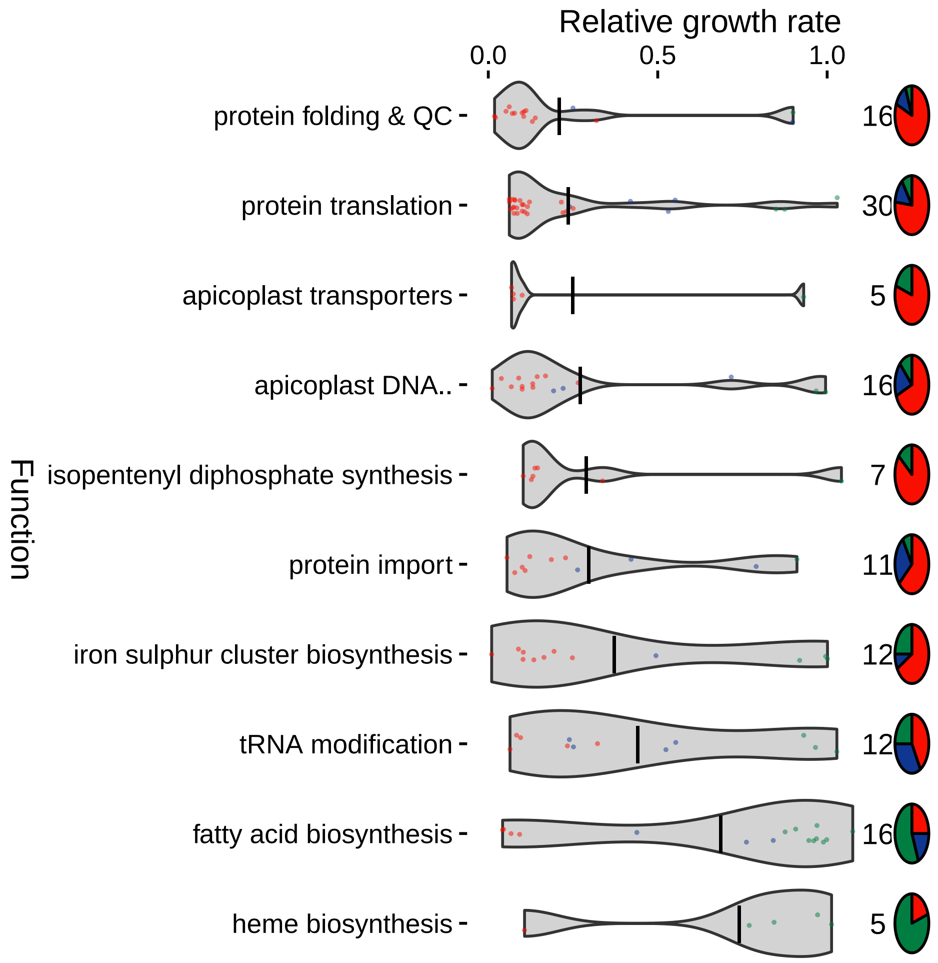


```
writeTable(merge, "apicoplast")
```

Integration efficiency

Next we will measure integration efficiency by combining 4 abundance measurements per gene-experiment, one pegged to each of the control genes. We normalise these to one another and then take their median. For dispensable genes this relative abundance represents integration efficiency. We find that the square root function normalises the distribution, and we measure its reproducibility, and model it using a loess model of the two homology arms.

```
cs <- addPhenotypes(singlecomb)
cs <- cs[sample(nrow(cs)), ]
cs <- cs[cs$phenotype == "Dispensable", ]
estimates <- as.matrix(cs[grep("normd6toinput", names(cs))])

factors <- colMeans(estimates, na.rm = T)
estimates2 <- sweep(estimates, c(2), factors, "/")
cs$median <- apply(estimates2, c(1), median, na.rm = T)
cs$sqrtmedian <- sqrt(cs$median)
cs <- cs[!is.na(cs$median) & is.finite(cs$median), ]


p1 <- ggplot(cs, aes(cs$median)) + geom_density(aes(color = "blue"), alpha = 0.1,
    fill = "blue") + stat_function(fun = dnorm, args = list(mean = mean(cs$median),
    sd = sd(cs$median)), aes(color = "red")) + xlab(expression(Integration ~
    efficiency)) + ylab("Density") + theme_classic() + theme(axis.line.x = element_line(color = "black"
    size = 0.5), axis.line.y = element_line(color = "black", size = 0.5)) +
    scale_x_continuous(expand = c(0, 0)) + scale_y_continuous(expand = c(0,
    0)) + coord_cartesian(xlim = c(0, 8)) + scale_colour_manual(name = "", values = c(blue = "blue",
    red = "red"), labels = c("Observed values", "Fitted normal distribution")) +
    theme(legend.position = "top")

p2 <- ggplot(cs, aes(cs$sqrtmedian)) + geom_density(color = "blue", fill = "blue",
    alpha = 0.1) + stat_function(fun = dnorm, args = list(mean = mean(cs$sqrtmedian),
    sd = sd(cs$sqrtmedian)), color = "red") + xlab(expression(sqrt(Integration ~
    efficiency))) + ylab("Density") + theme_classic() + theme(axis.line.x = element_line(color = "black
    size = 0.5), axis.line.y = element_line(color = "black", size = 0.5)) +
    scale_x_continuous(expand = c(0, 0)) + scale_y_continuous(expand = c(0,
    0)) + coord_cartesian(xlim = c(0, 3))

library(gridExtra)
get_legend <- function(myggplot) {
    tmp <- ggplot_gtable(ggplot_build(myggplot))
    leg <- which(sapply(tmp$grobs, function(x) x$name) == "guide-box")
    legend <- tmp$grobs[[leg]]
    return(legend)
}
legend <- get_legend(p1)
p1 <- p1 + theme(legend.position = "none")

grid.arrange(legend, p1, p2, ncol = 2, nrow = 2, layout_matrix = rbind(c(1,
    1), c(2, 3)), widths = c(2.7, 2.7), heights = c(0.2, 2.5))
```

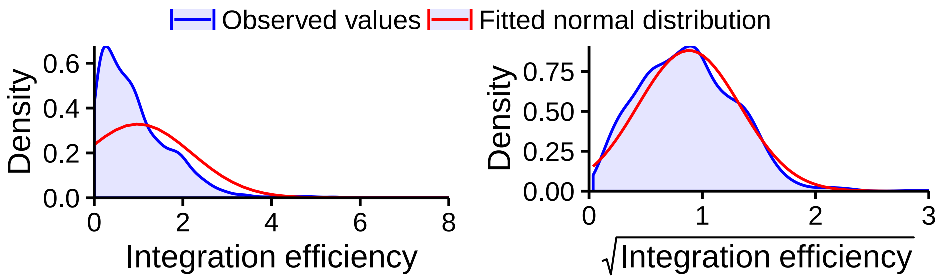

Reproducibility of integration efficiency

```
table <- as.data.frame(table(cs$cloneid))
multiples <- unique(table[table$Freq > 1, ]$Var1)
tempcs <- cs
matches <- match(multiples, cs$cloneid)
tempcs[matches, ]$cloneid = NA
matches <- match(multiples, cs$cloneid)
matches2 <- match(multiples, tempcs$cloneid)
df1 <- cs[matches, ]
df2 <- cs[matches2, ]

ggplot(df1, aes(df1$median, df2$median)) + geom_point() + geom_abline(intercept = 0) +
    labs(x = "Integration efficiency (experiment 1)", y = "Integration efficiency (experiment 2)") +
    theme_classic() + theme(axis.line.x = element_line(color = "black", size = 0.5),
    axis.line.y = element_line(color = "black", size = 0.5)) + scale_x_log10(breaks = c(0.1,
    1, 10), expand = c(0, 0)) + scale_y_log10(breaks = c(0.1, 1, 10), expand = c(0,
    0)) + coord_cartesian(ylim = c(0.05, 10), xlim = c(0.05, 10))
```

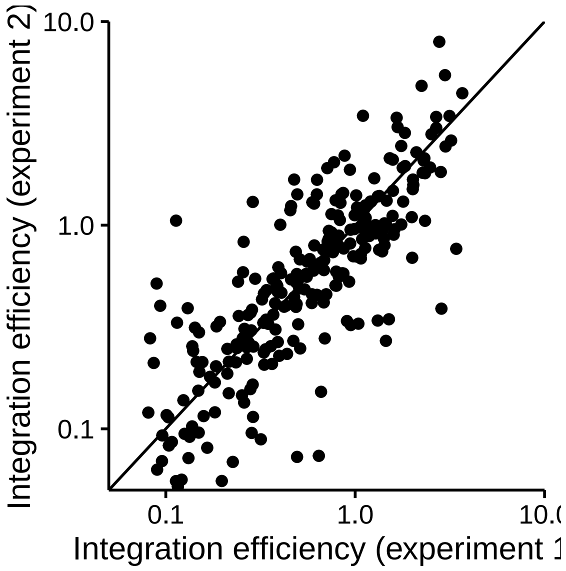

Modelling of integration efficiency

```r
csmono <- cs %>% group_by(cloneid, gene, PfID, current_version_ID) %>% summarise(timesAnalysed = length
    left_arm_length = mean(left_arm_length), right_arm_length = mean(right_arm_length),
    median = mean(median, na.rm = T))

elevation.loess = loess(median ~ left_arm_length * right_arm_length, data = csmono,
    degree = 2, span = 0.31)

elevation.fit = expand.grid(list(left_arm_length = seq(1, 11000, 400), right_arm_length = seq(1,
    11000, 400)))
z = predict(elevation.loess, newdata = elevation.fit)
elevation.fit$Height = as.numeric(z)

wireframe(Height ~ left_arm_length + right_arm_length, elevation.fit, zlab = list("Integration effiency
    rot = 90), xlab = "Left arm length", ylab = list("Right arm length", rot = 285),
    drape = TRUE, colorkey = TRUE, screen = list(z = 15, x = -65), scales = list(arrows = FALSE,
        cex = 0.5, tick.number = 5, z = list(arrows = T)))
```

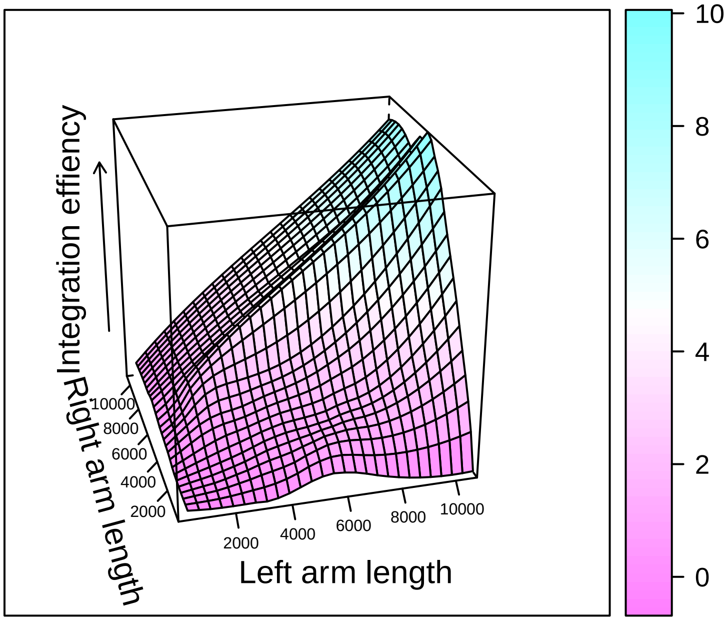

Look for an effect of homology arm length on phenotype calls

Here we split vectors up into equal sized groups by their homology arm length and observe phenotype distributions within each group, in case shorter homology arms cause increased essential calls due to the failure of the vector to integrate. We find that this effect, if exists, only applies to vectors with geometric mean homology arm length < 1.25 kb and we take account of this in the analysis.

```
mc <- addPhenotypes(multicomb)
mc <- mc[mc$phenotype != "Insufficient data", ]

mc$geometricmean = sqrt(mc$left_arm_length * mc$right_arm_length)
divisions = c(400, 1040, 1250, 1420, 1550, 1680, 1790, 1880, 2000, 2080, 2150,
    2200, 2300, 2360, 2440, 2510, 2580, 2670, 2760, 2850, 2940, 3060, 3190,
    3350, 3600, 3980, 7000)
lagdivisions = lag(divisions, 1)
groups = paste(lagdivisions, divisions, sep = " - ")[2:27]
mc$gmcut <- cut(mc$geometricmean, breaks = divisions, labels = groups)
levels(mc$gmcut) = gsub(",", " - ", levels(mc$gmcut))
levels(mc$gmcut) = gsub("[", "", levels(mc$gmcut), fixed = T)
levels(mc$gmcut) = gsub("(", "", levels(mc$gmcut), fixed = T)
levels(mc$gmcut) = gsub("]", "", levels(mc$gmcut), fixed = T)
levels(mc$gmcut) = gsub("e+0", "e", levels(mc$gmcut), fixed = T)
ggplot(mc, aes(x = gmcut, fill = phenotype)) + geom_bar(position = "fill") +
    scale_fill_manual(values = phenolevelscolor) + theme_bw() + theme(axis.text.x = element_text(angle =
    hjust = 1)) + labs(x = "Geometric mean arm length (range)", y = "Phenotype distribution",
    fill = "Phenotype") + scale_y_continuous(labels = scales::percent, expand = c(0,
    0))
```

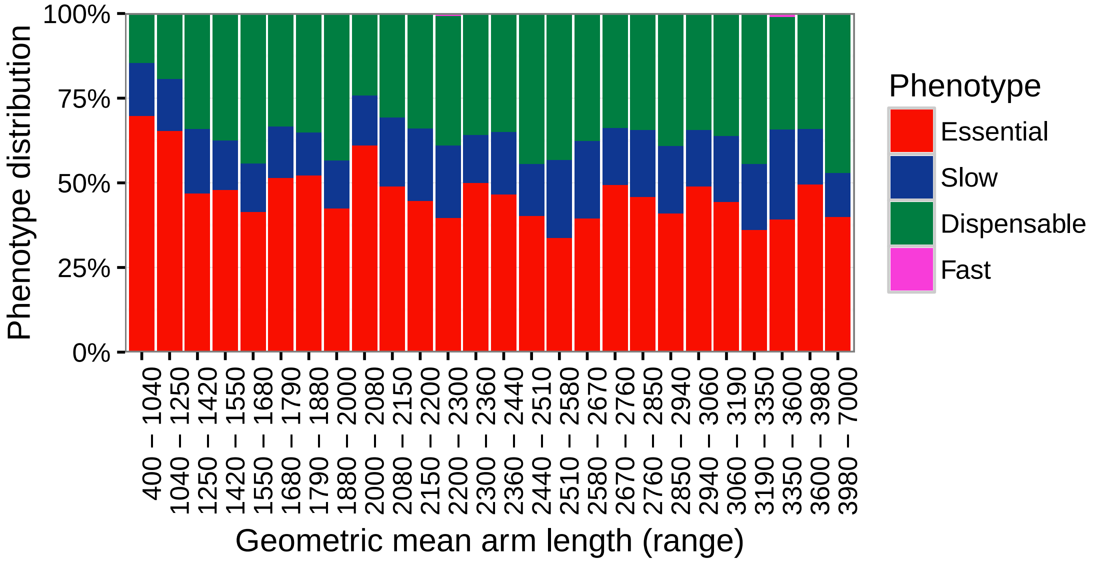


```
ggsave("output/homologyarmlength.pdf")
```

Expression pattern profiling

Next we perform k-means clustering using expression data from Otto *et al.* and observe the proportion of dispensable genes in the clusters compared to the proportion of sexual expression in the clusters.

```
set.seed(283)
data<-read.csv("./otherdata/ExpressionPatternOttoEtAl.csv",header=T,stringsAsFactors = FALSE)
cs<-addPhenotypes(multicomb)
cs<- filter(cs,sqrt(left_arm_length*right_arm_length)>1250)
cs<-cs[cs$phenotype!="Insufficient data",]
m<-merge(cs,data,by.x="gene",by.y="Gene",all.y=F)
df=m[m$Total>0,c("Ring","Tro","Sch","Gam","Ook")]


clusters<-kmeans(df, 9)


df$cluster=clusters$cluster

df$Ring=as.numeric(as.character(df$Ring))
df$Tro=as.numeric(as.character(df$Tro))
df$Sch=as.numeric(as.character(df$Sch))
df$Gam=as.numeric(as.character(df$Gam))
df$Ook=as.numeric(as.character(df$Ook))
df$sexiness=df$Gam+df$Ook
bigdf=cbind(m[m$Total>0,],df)
df$binarised=ifelse(bigdf$phenotype %in% c("Essential", "Slow"),0,1)




meltall<-melt(bigdf,id.vars=c("cluster","gene"))
meltall$cluster=factor(meltall$cluster)
df2<- as.data.frame(df %>%
group_by(cluster) %>%
summarise_each(funs(mean(., na.rm = TRUE))))

df2<-df2[order(df2$sexiness),]
df2$cluster=factor(as.character(df2$cluster),levels=as.character(df2$cluster))
meltall$cluster=factor(as.character(meltall$cluster),levels=as.character(df2$cluster))

cor<-cor.test(df2$binarised,df2$sexiness)$estimate

melt<-melt(df2,id.vars="cluster")
co<-coef(lm(binarised ~ sexiness, data = df2))
mylabel = paste("italic(R)^2 == ",round(cor^2,3))

df$cluster=factor(df$cluster)
pal<-c("#e41a1c","#377eb8","#4daf4a","#984ea3","#ff7f00","#ffff33","#8dd3c7","#f781bf","#a65628")
p1<- ggplot(df2,aes(x=sexiness,y=binarised,fill=cluster))+geom_point(color="black",shape=21,size=4,strol
panel.grid = element_blank()


)
```

```
annotation=as.data.frame(table(df$cluster))
colnames(annotation)=c("cluster","n")

p2<-ggplot(meltall[(meltall$variable %in% c("Ring","Tro","Sch","Gam","Ook")),],aes(x=as.factor(variable)
panel.grid = element_blank()


)


grid.arrange(p2,p1,ncol=2)
```

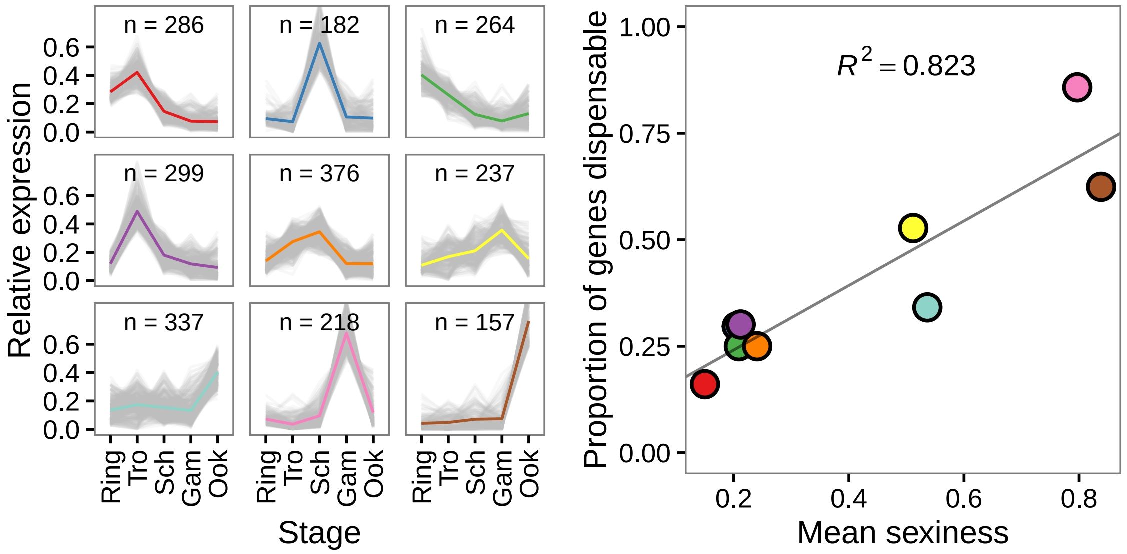


```
df2$orderedcluster=1:9
bigdf$orderedcluster=df2[match(as.character(bigdf$cluster),as.character(df2$cluster)),"orderedcluster"]
subcols=bigdf[,c("orderedcluster","gene","gene_name","gene_product","phenotype","Relative.Growth.Rate",
write.csv(subcols,"output/expressionphenotypes.csv")
```

Is the resource representative of the genome?

We will now test whether the resource is representative of the genome in terms of basic gene properties: gene length, AT-content and expression levels.

```
data <- addPhenotypes(multicomb)

pbgenes <- read.table("otherdata/pbergheigenes.txt", stringsAsFactors = F, sep = "\t",
    header = T)
library(stringr)
pbgenes$length = nchar(pbgenes$code)
pbgenes$Acount = str_count(pbgenes$code, pattern = "A")
pbgenes$Tcount = str_count(pbgenes$code, pattern = "T")
pbgenes$Gcount = str_count(pbgenes$code, pattern = "G")
pbgenes$Ccount = str_count(pbgenes$code, pattern = "C")
pbgenes$at = (pbgenes$Acount + pbgenes$Tcount)/pbgenes$length

small = as.data.frame(data[, c("current_version_ID", "gene")])
pbgenes$included = "All"

shared <- merge(pbgenes, small, by.x = "PbNew", by.y = "current_version_ID")

shared$included = "Yes"

combo <- bind_rows(pbgenes, shared)
genomiclocations$totallength = genomiclocations$end - genomiclocations$start


originalgeneset <- read.csv("otherdata/plasmogemenrichment.csv")
merge <- merge(originalgeneset, geneinfo, by.x = "gene", by.y = "Old.Gene.ID")
merge <- merge(merge, genomiclocations, by.x = "current_version_ID", by.y = "gene")

merge <- merge(merge, pbgenes, by.x = "current_version_ID", by.y = "PbNew")
merge$included = "all"


merge2 <- merge[merge$gene %in% data$gene, ]
merge2$included = "yes"

combo2 <- bind_rows(merge, merge2)


ggplot(combo2, aes(x = totallength, color = included)) + geom_density() + scale_x_log10() +
    theme_classic() + theme(axis.line.x = element_line(color = "black", size = 0.5),
    axis.line.y = element_line(color = "black", size = 0.5)) + scale_y_continuous(expand = c(0,
    0), breaks = NULL)
```

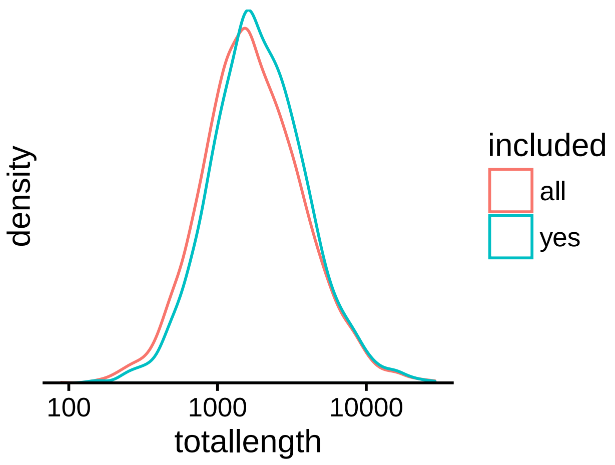


```
ggplot(combo2, aes(x = at, color = included)) + geom_density() + theme_classic() +
    theme(axis.line.x = element_line(color = "black", size = 0.5), axis.line.y = element_line(color = "
        size = 0.5)) + scale_y_continuous(expand = c(0, 0), breaks = NULL) +
    scale_x_continuous(labels = scales::percent) + theme(legend.position = "bottom")
```

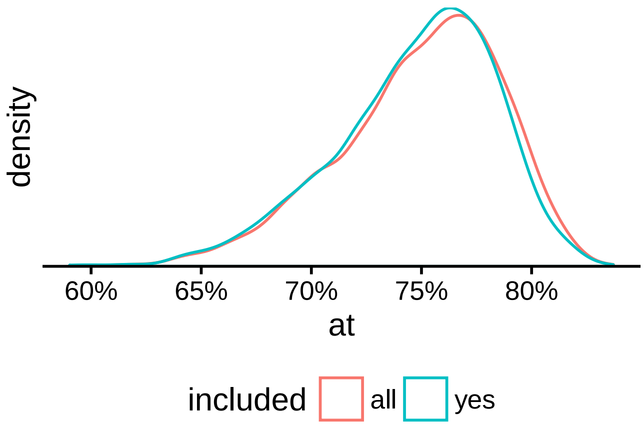


```
ggsave("ATcontent.pdf", width = 2.5, height = 2.7)

rpkm <- read.csv("otherdata/OttoEtAlRPKM.csv")
rpkm$Ring = (rpkm$Ring1 + rpkm$Ring2)/2
rpkm$Tro = (rpkm$Tro1 + rpkm$Tro2)/2
rpkm$Sch = (rpkm$Sch1 + rpkm$Sch2)/2
rpkm$Gam = (rpkm$Gam1 + rpkm$Gam2)/2
rpkm$Ook = (rpkm$Ook1 + rpkm$Ook2)/2
rpkm$Ring = (rpkm$Ring1 + rpkm$Ring2)/2

rpkm$rankRing = rank(-rpkm$Ring)
rpkm$rankTro = rank(-rpkm$Tro)
rpkm$rankSch = rank(-rpkm$Sch)
rpkm$rankGam = rank(-rpkm$Gam)
rpkm$rankOok = rank(-rpkm$Ook)
rpkm$Gene = as.character(rpkm$Gene)
combo2$current_version_ID = as.character(combo2$current_version_ID)
combo2$code = NULL
```

```
abc <- merge(combo2, rpkm, by.x = "gene", by.y = "Gene")

subset <- abc[, c("included", "Ring", "Tro", "Sch", "Gam", "Ook")]
# subset<-abc[,c('included','rankRing','rankTro','rankSch','rankGam','rankOok')]
melted <- melt(subset, id.vars = "included")
ggplot(melted, aes(x = value)) + geom_density(color = "gray", data = filter(melted,
    included == "all")) + geom_density(color = "blue", data = filter(melted,
    included == "yes")) + theme_classic() + theme(axis.line.x = element_line(color = "black",
    size = 0.5), axis.line.y = element_line(color = "black", size = 0.5)) +
    scale_y_continuous(expand = c(0, 0), breaks = NULL) + facet_grid(variable ~
    .) + scale_x_log10() + labs(x = "RPKM", y = "Relative frequency")
```

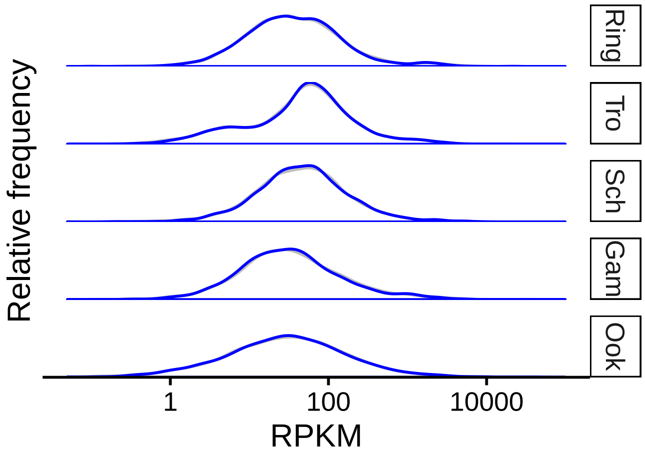


```
ggsave("ExpressionTargeting.pdf", width = 2.7, height = 2.7)
```

We will also check that there are not major biases in gene selection in the portion of the genome we phenotyped by checking for any over-represented GO terms, excluding them and looking at phenotype distribution again. (There is no substantial change.)

```
algorithm = "weight01"
pval = 0.05
mergedf = NULL
types <- c("BP", "MF", "CC")
for (t in 1:3) {
    gomappf <- read.csv("./otherdata/geneid2gopf.csv", stringsAsFactors = FALSE)
    gomappb <- read.csv("./otherdata/geneid2gopbnew.csv", stringsAsFactors = FALSE)
    nonproteincoding <- read.csv("./otherdata/NonProteinCoding", stringsAsFactors = FALSE)
    info <- geneinfo[, c("Old.Gene.ID", "current_version_ID", "PfID")]
    info <- info[!(info$current_version_ID %in% nonproteincoding$gene), ]
    gomappf <- merge(info, gomappf, by.y = "ID", by.x = "PfID")
    gomappb <- merge(info, gomappb, by.y = "ID", by.x = "current_version_ID")
    gomap <- rbind(gomappf, gomappb)[, c("Old.Gene.ID", "GO")]
    colnames(gomap) = c("ID", "GO")
    gomap <- unique(gomap)
    go <- aggregate(GO ~ ID, data = gomap, c)
    godb <- setNames(as.list(go$GO), go$ID)
    comb <- addPhenotypes(multicomb)
    comb <- comb[comb$phenotype != "Insufficient data", ]
    myInterestingGenes <- comb$gene
    geneList <- factor(as.integer((go$ID %in% myInterestingGenes)))
```

```r
    names(geneList) <- go$ID
    GOdata <- new("topGOdata", ontology = types[t], allGenes = geneList, annot = annFUN.gene2GO,
        gene2GO = godb)
    resultant <- runTest(GOdata, algorithm = algorithm, statistic = "fisher")
    allRes <- GenTable(GOdata, res = resultant, orderBy = "res", ranksOf = "res",
        topNodes = min(200, length(resultant@score)))
    tempdf <- as.data.frame(allRes)
    tempdf <- tempdf[tempdf$res < pval, ]
    myterms = tempdf$GO.ID
    mygenes <- genesInTerm(GOdata, myterms)
    termdf = NULL
    for (i in 1:length(myterms)) {
        myterm <- myterms[i]
        mygenesforterm <- mygenes[myterm][[1]]
        temptermdf <- data.frame(term = rep(myterm, length(mygenesforterm)),
            ID = mygenesforterm)
        if (!is.null(termdf)) {
            termdf <- rbind(termdf, temptermdf)
        } else {
            termdf <- temptermdf
        }



    }
    tempdf$type = ontology = types[t]
    tempdf <- merge(tempdf, termdf, by.x = "GO.ID", by.y = "term")
    if (!is.null(mergedf)) {

        mergedf <- rbind(mergedf, tempdf)
    } else {
        mergedf <- tempdf
    }

}

adddata <- merge(mergedf, comb, by.x = "ID", by.y = "gene")


all <- addPhenotypes(multicomb)
all <- all[all$phenotype != "Insufficient data", ]

notenriched <- all[!(all$gene %in% adddata$ID), ]
enriched <- all[(all$gene %in% adddata$ID), ]


all$class = "Overall screen"
notenriched$class = "Enriched GO\n terms removed"

ggplot(rbind(all, notenriched), aes(x = factor(as.character(class), levels = c("Overall screen",
    "Enriched GO\n terms removed")), fill = phenotype)) + theme_classic() +
    geom_bar(position = "fill", width = 0.5) + scale_fill_manual(values = phenolevelscolor) +
    scale_y_continuous(expand = c(0, 0), labels = scales::percent) + labs(x = "",
```

```
    y = "Phenotype proportions", fill = "Phenotype") + theme(axis.line.x = element_line(color = "black"
    size = 0.5), axis.line.y = element_line(color = "black", size = 0.5))
```

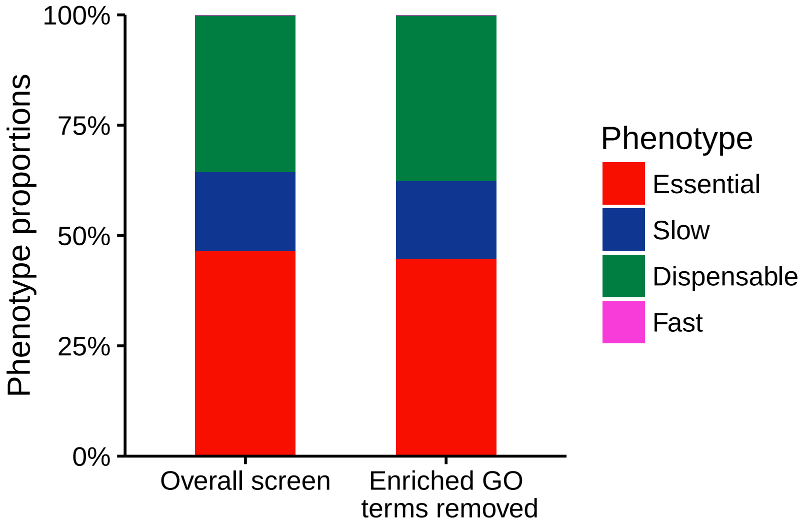

We can also plot a simpler scatter plot to observe if functional categories are generally represented similarly to the genome as a whole. Since BIR and FAM families are known to have grouped phenotypes but are not represented in GO terms we have manually added these categories to the plot. This demonstrates a depletion in BIR genes, but these only correspond to 2% of *P. berghei* genes.

```
m <- merge(gomappb, all, all.x = T)
m$inscreen = ifelse(!is.na(m$phenotype), "yes", "no")
df <- as.data.frame.matrix(table(m$GO, m$inscreen))
birgenes = data.frame(yes = 15, no = 113 - 15)
famgenes = data.frame(yes = 12, no = 25 - 12)
ggplot(df, aes(x = yes + no + 0.5, y = yes + 0.5)) + geom_point(alpha = 1, size = 0.5) +
    scale_x_log10() + scale_y_log10() + labs(x = "Total number of genes annotated with term",
    y = "Screened genes annotated with term") + geom_point(data = birgenes,
    color = "red", size = 0.5) + geom_point(data = famgenes, color = "blue",
    size = 0.5) + theme_bw()
```

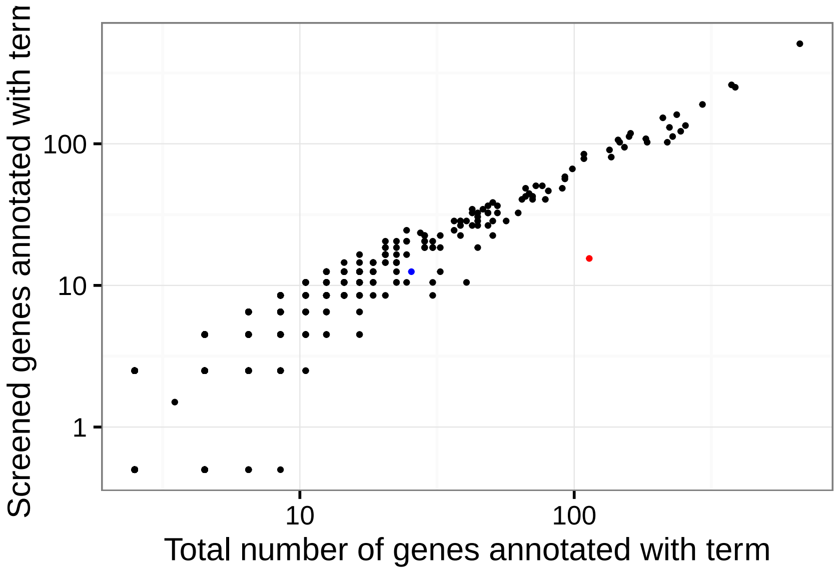


```
ggsave("GoScatter.pdf", width = 2.5, height = 2.5)
```

Gene ontology enrichment

Next we we use GO annotation from both *P. berghei* and the better annotated *P. falciparum* to check for enrichment in *Dispensable*, *Slow* and *Essential* genes. We use the *topGO* algorithm *weight01* which helps to control for false-positives by using the structure of the GO hierarchy.

```
algorithm = "weight01"
pval = 0.05
mergedf = NULL

types <- c("BP", "MF", "CC")
phens <- c("Essential", "Slow", "Dispensable")
for (p in 1:3) {
    for (t in 1:3) {

        gomappf <- read.csv("./otherdata/geneid2gopf.csv", stringsAsFactors = FALSE)
        gomappb <- read.csv("./otherdata/geneid2gopbnew.csv", stringsAsFactors = FALSE)
        info <- geneinfo[, c("Old.Gene.ID", "current_version_ID", "PfID")]

        gomappf <- merge(info, gomappf, by.y = "ID", by.x = "PfID")

        gomappb <- merge(info, gomappb, by.y = "ID", by.x = "current_version_ID")

        gomap <- rbind(gomappf, gomappb)[, c("Old.Gene.ID", "GO")]
        colnames(gomap) = c("ID", "GO")
        gomap <- unique(gomap)

        go <- aggregate(GO ~ ID, data = gomap, c)
        godb <- setNames(as.list(go$GO), go$ID)
        comb <- addPhenotypes(multicomb)
        comb <- comb[comb$phenotype != "Insufficient data", ]
        allgenes = setNames(rep(1, length(comb$gene)), comb$gene)
        myInterestingGenes <- comb[comb$phenotype == phens[p], ]$gene
        geneList <- factor(as.integer(comb$gene %in% myInterestingGenes))
        names(geneList) <- comb$gene


        GOdata <- new("topGOdata", ontology = types[t], allGenes = geneList,
            annot = annFUN.gene2GO, gene2GO = godb)

        resultant <- runTest(GOdata, algorithm = algorithm, statistic = "fisher")

        allRes <- GenTable(GOdata, res = resultant, orderBy = "res", ranksOf = "res",
            topNodes = min(200, length(resultant@score)))


        tempdf <- as.data.frame(allRes)
        tempdf <- tempdf[tempdf$res < pval, ]

        myterms = tempdf$GO.ID
        mygenes <- genesInTerm(GOdata, myterms)
        termdf = NULL
        for (i in 1:length(myterms)) {
            myterm <- myterms[i]
```

```
            mygenesforterm <- mygenes[myterm][[1]]
            temptermdf <- data.frame(term = rep(myterm, length(mygenesforterm)),
                ID = mygenesforterm)
            if (!is.null(termdf)) {
                termdf <- rbind(termdf, temptermdf)
            } else {
                termdf <- temptermdf
            }



        }
        tempdf$type = ontology = types[t]
        tempdf$phen = phens[p]
        tempdf <- merge(tempdf, termdf, by.x = "GO.ID", by.y = "term")
        if (!is.null(mergedf)) {

            mergedf <- rbind(mergedf, tempdf)
        } else {
            mergedf <- tempdf
        }
    }
}

pvals <- mergedf %>% group_by(GO.ID, Term, phen) %>% summarise(p = unique(res))
write.csv(pvals, "output/GOpvals.csv")


adddata <- merge(mergedf, comb, by.x = "ID", by.y = "gene")
subcols <- adddata[, c("GO.ID", "Term", "ID", "gene_product", "phenotype", "Relative.Growth.Rate",
    "Confidence")]
write.csv(subcols, "output/GOgenes.csv")
adddata$xlabel <- paste(adddata$type, adddata$Term, sep = ": ")
adddata$termphen <- paste(adddata$Term, adddata$phen, sep = "")

agg <- aggregate(Relative.Growth.Rate ~ termphen + phen, data = adddata, mean)

agg$phen = factor(as.character(agg$phen), levels = c("Essential", "Slow", "Dispensable"))
agglength <- aggregate(Relative.Growth.Rate ~ termphen, data = adddata, length)
grouped <- group_by(adddata, termphen, Term, phen)
summary <- summarise(grouped, mean = mean(Relative.Growth.Rate), count = n())

agg <- agg[order(agg$phen, agg$Relative.Growth.Rate), ]

adddata$termphen = factor(as.character(adddata$termphen), levels = as.character(agg$termphen))
adddata$phen = factor(as.character(adddata$phen), levels = c("Essential", "Slow",
    "Dispensable"))




give.n <- function(x) {
    return(c(y = 1.15, label = length(x)))
```

```
}


p1 <- ggplot(adddata, aes(x = 1, fill = phenotype)) + theme_bw() + geom_bar(position = "fill",
    color = "black") + coord_polar(theta = "y", start = -3.14/2) + facet_grid(. ~
    termphen) + scale_fill_manual(values = phenolevelscolor) + theme(axis.ticks = element_blank(),
    axis.text.y = element_blank(), axis.text.x = element_blank()) + theme(panel.border = element_blank(
    panel.grid.major = element_blank(), panel.grid.minor = element_blank(),
    axis.line = element_line(colour = "black"))


p2 <- ggplot(adddata, aes(x = Term, y = Relative.Growth.Rate)) + theme_bw() +
    geom_violin(aes(color = phen), fill = "lightgray") + theme(axis.text.x = element_text(angle = 90,
    hjust = 1, vjust = 0.5, size = 15, color = "black")) + scale_color_manual(values = phenolevelscolor
    theme(strip.text.x = element_blank()) + theme(strip.background = element_blank()) +
    labs(y = "Relative growth rate") + geom_jitter(height = 0, width = 0.3,
    alpha = 0.1) + stat_summary(fun.y = "mean", colour = "black", size = 4,
    geom = "point") + facet_grid(. ~ termphen, space = "free_x", scale = "free_x") +
    stat_summary(fun.data = give.n, geom = "text", fun.y = median, angle = 90) +
    scale_y_continuous(breaks = c(0, 0.5, 1), limits = c(0, 1.25)) + theme(panel.border = element_blank
    panel.grid.major = element_blank(), panel.grid.minor = element_blank(),
    axis.line = element_line(colour = "black"))


g2 <- ggplotGrob(p1)
g1 <- ggplotGrob(p2)


pp <- c(subset(g1$layout, grepl("panel", g1$layout$name), select = t:r))
top = unique(pp$t)

thenames <- g1$layout[g1$layout$t == top, ]$name

g1 <- gtable_add_rows(g1, unit(0.1, "null"), pos = unique(pp$t) - 1)


g <- gtable_add_grob(g1, g2$grobs[grepl("panel", g1$layout$name)], pp$t, pp$l,
    pp$b, pp$l)
g$layout[g$layout$name %in% thenames, ]$t = top


grid.newpage()
pushViewport(viewport(angle = -90, width = unit(30, "inches"), height = unit(8.5,
    "inches")))

grid.draw(g)
```

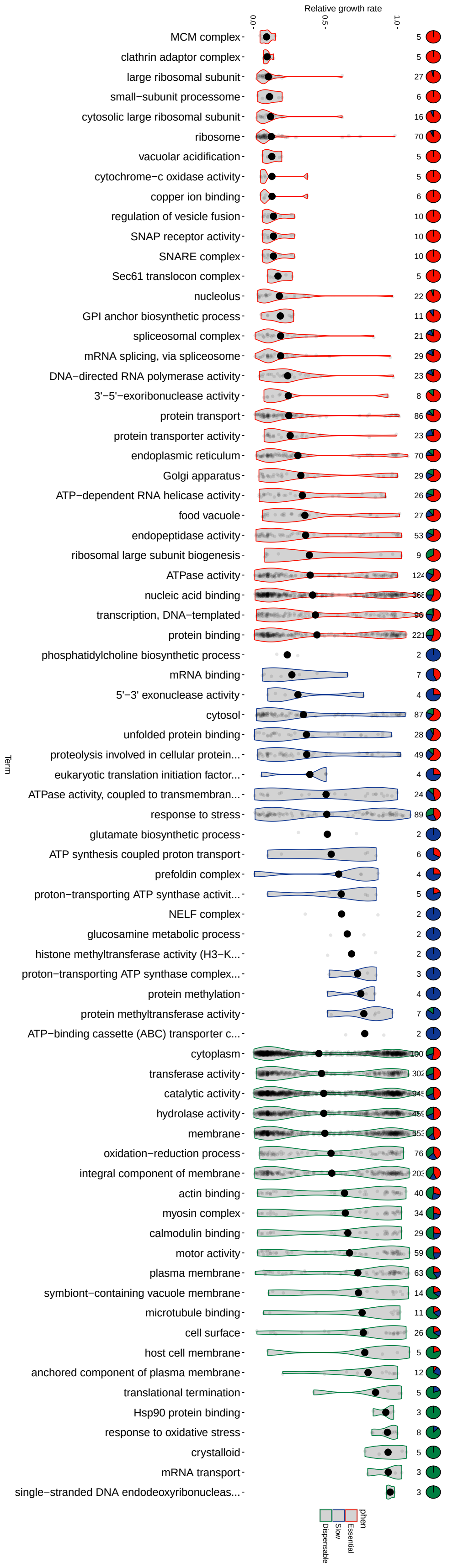
Relative growth rate
0.0
0.5
1.0
Term
MCM complex
clathrin adaptor complex
large ribosomal subunit
small−subunit processome
cytosolic large ribosomal subunit
ribosome
vacuolar acidification
cytochrome−c oxidase activity
copper ion binding
regulation of vesicle fusion
SNAP receptor activity
SNARE complex
Sec61 translocon complex
nucleolus
GPI anchor biosynthetic process
spliceosomal complex
mRNA splicing, via spliceosome
DNA−directed RNA polymerase activity
3'−5'−exoribonuclease activity
protein transport
protein transporter activity
endoplasmic reticulum
Golgi apparatus
ATP−dependent RNA helicase activity
food vacuole
endopeptidase activity
ribosomal large subunit biogenesis
ATPase activity
nucleic acid binding
transcription, DNA−templated
protein binding
phosphatidylcholine biosynthetic process
mRNA binding
5'−3' exonuclease activity
cytosol
unfolded protein binding
proteolysis involved in cellular protein...
eukaryotic translation initiation factor...
ATPase activity, coupled to transmembran...
response to stress
glutamate biosynthetic process
ATP synthesis coupled proton transport
prefoldin complex
proton−transporting ATP synthase activit...
NELF complex
glucosamine metabolic process
histone methyltransferase activity (H3−K...
proton−transporting ATP synthase complex...
protein methylation
protein methyltransferase activity
ATP−binding cassette (ABC) transporter c...
cytoplasm
transferase activity
catalytic activity
hydrolase activity
membrane
oxidation−reduction process
integral component of membrane
actin binding
myosin complex
calmodulin binding
motor activity
plasma membrane
symbiont−containing vacuole membrane
microtubule binding
cell surface
host cell membrane
anchored component of plasma membrane
translational termination
Hsp90 protein binding
response to oxidative stress
crystalloid
mRNA transport
single−stranded DNA endodeoxyribonucleas...
phen
Essential
Slow
Dispensable

Comparison with yeast phenotypes

We here compare our results to those in the yeast *S. cerevisiae* - we observe that attenuated growth is well conserved, as is essentiality, but that dispensable genes in *S. cerevisiae* often have essential *P. berghei* orthologs.

```
giaever <- read.table("./otherdata/yeastphenotypes.tsv", sep = "\t", header = T)
cs <- addPhenotypes(multicomb)

m <- merge(cs, giaever, by.x = "gene", by.y = "PbID")
m <- m[m$Yeastpheno != "", ]
m$Yeastpheno = as.character(m$Yeastpheno)
m[m$Yeastpheno == "competitive fitness: decreased ", ]$Yeastpheno = "slow"
m <- m[m$phenotype != "Insufficient data", ]
ggplot(m, aes(x = phenotype, fill = phenotype)) + geom_bar() + facet_wrap(~Yeastpheno,
    scales = "free_y") + scale_fill_manual(values = phenolevelscolor) + theme_bw() +
    theme(axis.title.x = element_blank(), axis.text.x = element_blank(), axis.ticks.x = element_blank()
    labs(fill = "Phenotype", y = "Number of genes")
```

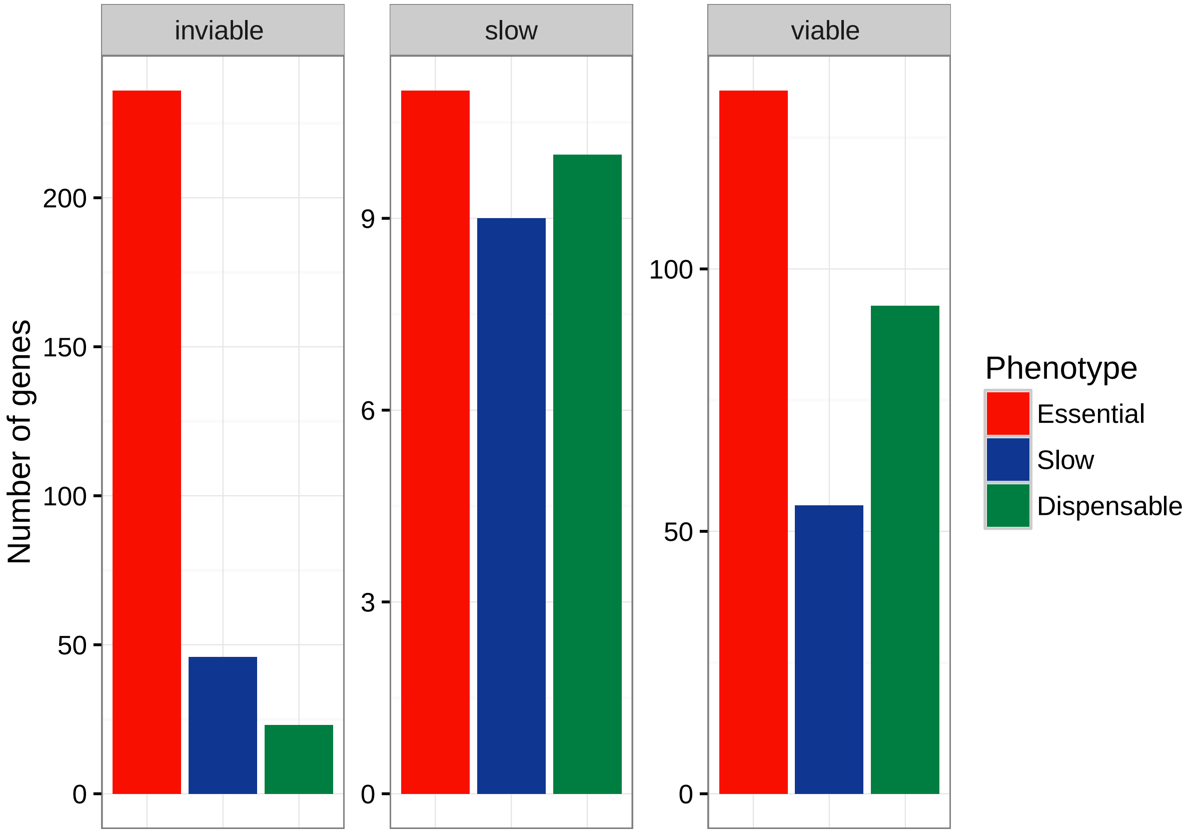


```
table(m$phenotype, m$Yeastpheno)
```

```
##
##                     inviable slow viable
##   Insufficient data        0    0      0
##   Essential              236   11    134
##   Slow                    46    9     55
##   Dispensable             23   10     93
```

```
##   Fast                            0    0       0
##   Unselected                      0    0       0
```

Apicomplexan comparisons

Naturally, one can only make comparisons between orthologous genes. However, as we have already seen, conservation is linked to increased essentiality. Thus by limiting ourselves to 1:1 genes we will inherently enrich for orthologous genes. It is important to be aware of this effect, hence we plot it below - both for our own data and that from Sidik *et al.*

```
data <- addPhenotypes(multicomb)
OT <- read.csv("./otherdata/OrthologTable.csv")
data$ortholog = ifelse(data$current_version_ID %in% OT$PbID, "1:1 ortholog in Tg",
    "No 1:1 ortholog in Tg")
p1 <- ggplot(data, aes(color = ortholog, x = Relative.Growth.Rate)) + geom_density() +
    labs(x = "Pb phenotype", y = "Relative frequency") + theme_classic() + theme(legend.position = "bot
    theme(axis.line.x = element_line(color = "black", size = 0.5), axis.line.y = element_line(color = "
        size = 0.5)) + scale_y_continuous(expand = c(0, 0)) + scale_x_continuous(expand = c(0,
    0))
sidik <- read.csv("./otherdata/sidiketalwithorthologs.csv")
sidik$ortholog = ifelse(sidik$TgID %in% OT$TgID, "1:1 ortholog in Pb", "No 1:1 ortholog in Pb")
p2 <- ggplot(sidik, aes(color = ortholog, x = toxophenotype)) + geom_density() +
    labs(x = "Tg phenotype", y = "Relative frequency") + theme_classic() + theme(legend.position = "bot
    theme(axis.line.x = element_line(color = "black", size = 0.5), axis.line.y = element_line(color = "
        size = 0.5)) + scale_y_continuous(expand = c(0, 0)) + scale_x_continuous(expand = c(0,
    0))
grid.arrange(p1, p2, ncol = 2)
```

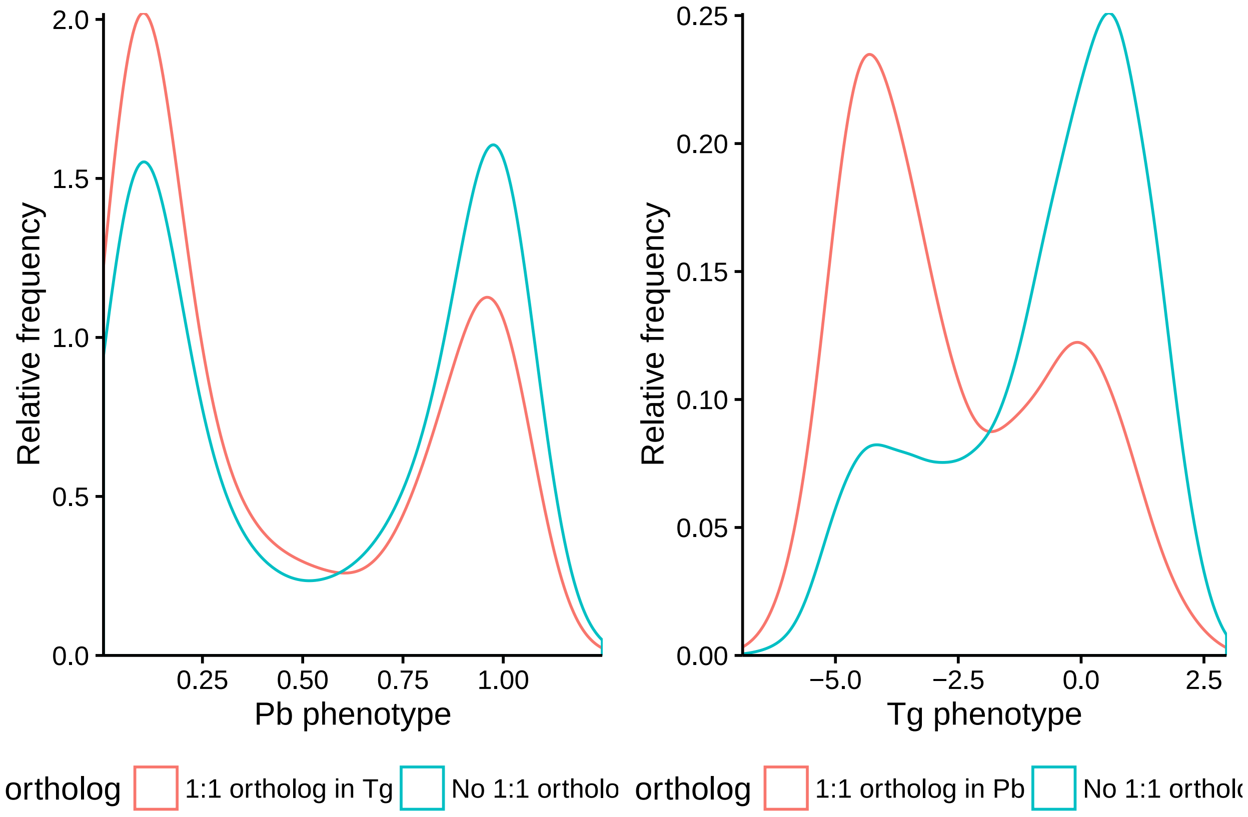

Now for those where there are orthologues, let's look at phenotype conservation:

```
dataset <- addPhenotypes(multicomb)

# Toxoplasma data from Sidik et al.
toxo <- read.csv("./otherdata/sidiketalwithorthologs.csv")
toxoortho <- subset(toxo, PbID != "#N/A")

merge <- merge(dataset, toxoortho, by.x = "current_version_ID", by.y = "PbID")  #Pb/Toxo available orth
nrow(merge)
```

```
## [1] 1165
```

```
write.csv(merge, "pbtg.csv")

pf <- read.table("otherdata/phenoplasm2.txt", header = T)
merge2 <- merge(merge, pf, by.x = "PfID.x", by.y = "gene")  #Pb/Toxo/Pf available orthologs
write.csv(merge2, "pbpftg.csv")
nrow(merge2)
```

```
## [1] 62
```

```
merge2$targetability = as.character(merge2$targetability)
merge2$targetability[merge2$targetability == "V"] = "Viable"
merge2$targetability[merge2$targetability == "R"] = "Modification unsuccessful"

p3 <- ggplot(merge, aes(y = toxophenotype, x = Relative.Growth.Rate)) + geom_point(color = "#b0b0b0",
    alpha = 0.3, stroke = 0) + geom_point(data = merge2, aes(color = targetability)) +
    theme_bw() + scale_x_continuous(breaks = c(0, 0.5, 1)) + scale_color_manual(values = c("#f90f00",
    "#007e41")) + labs(y = "T. gondii phenotype", x = "P. berghei RGR", color = "P. falciparum transfec
    scale_y_continuous(breaks = c(-7.5, -5, -2.5, 0, 2.5)) + coord_cartesian(xlim = c(0,
    1.1), ylim = c(-7.5, 2.5)) + theme(legend.position = "bottom") + theme(panel.grid.major = element_bl
    panel.grid.minor = element_blank())




p5 <- ggplot(merge, aes(y = toxophenotype, x = Relative.Growth.Rate)) + geom_point(color = "black",
    alpha = 0.4, stroke = 0, size = 0.5) + theme_bw() + scale_x_continuous(breaks = c(0,
    0.5, 1)) + scale_color_manual(values = c("#007e41", "#f90f00")) + labs(y = "T. gondii phenotype",
    x = "P. berghei RGR", color = "P. falciparum transfections") + scale_y_continuous(breaks = c(-7.5,
    -5, -2.5, 0, 2.5)) + coord_cartesian(xlim = c(0, 1.1), ylim = c(-7.5, 2.5)) +
    theme(legend.position = "bottom") + theme(panel.grid.major = element_blank(),
    panel.grid.minor = element_blank()) + geom_smooth(method = "lm")


p11 <- ggplot(merge, aes(y = toxophenotype, x = Relative.Growth.Rate)) + theme_bw() +
    scale_x_continuous(breaks = c(0, 0.5, 1)) + scale_color_manual(values = c("#007e41",
    "#f90f00")) + labs(y = "T. gondii phenotype", x = "P. berghei RGR", color = "P. falciparum transfec
    scale_y_continuous(breaks = c(-7.5, -5, -2.5, 0, 2.5)) + coord_cartesian(xlim = c(0,
    1.1), ylim = c(-7.5, 2.5)) + theme(legend.position = "bottom") + theme(panel.grid.major = element_bl
    panel.grid.minor = element_blank()) + stat_density_2d(geom = "raster", aes(fill = ..density..),
    contour = FALSE) + guides(fill = FALSE) + scale_fill_distiller(palette = "Spectral")
```

The density plot again reveals the high degree of essentiality in the orthologues, and also shows significant correlation.

```
p11
```

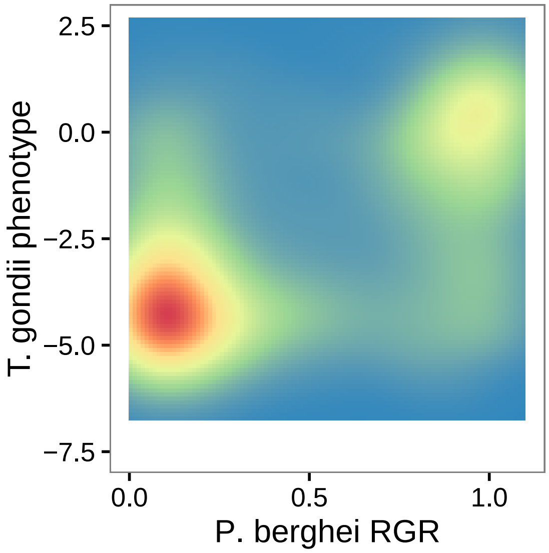

But though the correlation is highly significant there is also a large portion of variation unexplained by it.

```
cor <- cor.test(merge$toxophenotype, merge$Relative.Growth.Rate)

r <- cor$estimate
r
```

```
##       cor
## 0.5403165
```

```
r^2
```

```
##       cor
## 0.2919419
```

```
p <- cor$p.value
p
```

```
## [1] 3.989104e-89
```

Plotting a scatter plot with the fitted linear regression shows there are a significant number of non-conserved phenotypes.

```
p5
```

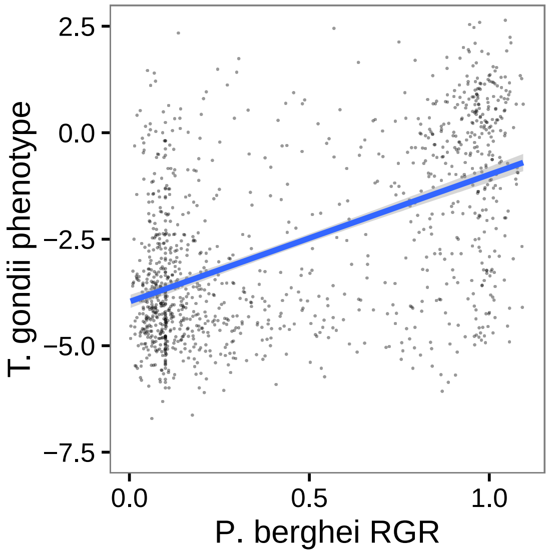

We can look for GO enrichment for the discordant quadrants of the scatterplot.

```
algorithm = "weight01"
pval = 0.05
mergedf = NULL
types <- c("BP", "MF", "CC")
interesting <- filter(merge, toxophenotype < (-2) & Relative.Growth.Rate > 0.4)$gene
# interesting<-filter(merge,toxophenotype>(-2) &
# Relative.Growth.Rate<0.4)$gene
for (t in 1:3) {
    gomappf <- read.csv("./otherdata/geneid2gopf.csv", stringsAsFactors = FALSE)
    gomappb <- read.csv("./otherdata/geneid2gopbnew.csv", stringsAsFactors = FALSE)

    info <- geneinfo[, c("Old.Gene.ID", "current_version_ID", "PfID")]

    gomappf <- merge(info, gomappf, by.y = "ID", by.x = "PfID")
    gomappb <- merge(info, gomappb, by.y = "ID", by.x = "current_version_ID")
    gomap <- rbind(gomappf, gomappb)[, c("Old.Gene.ID", "GO")]
    colnames(gomap) = c("ID", "GO")
    gomap <- unique(gomap)
    go <- aggregate(GO ~ ID, data = gomap, c)
    go <- go[go$ID %in% merge$gene, ]
    godb <- setNames(as.list(go$GO), go$ID)

    geneList <- factor(as.integer((go$ID %in% interesting)))
    names(geneList) <- go$ID
    GOdata <- new("topGOdata", ontology = types[t], allGenes = geneList, annot = annFUN.gene2GO,
        gene2GO = godb)
    resultant <- runTest(GOdata, algorithm = algorithm, statistic = "fisher")
    allRes <- GenTable(GOdata, res = resultant, orderBy = "res", ranksOf = "res",
        topNodes = min(200, length(resultant@score)))
    tempdf <- as.data.frame(allRes)
    tempdf <- tempdf[tempdf$res < pval, ]
    myterms = tempdf$GO.ID
    mygenes <- genesInTerm(GOdata, myterms)
    termdf = NULL
```

```
    for (i in 1:length(myterms)) {
        myterm <- myterms[i]
        mygenesforterm <- mygenes[myterm][[1]]
        temptermdf <- data.frame(term = rep(myterm, length(mygenesforterm)),
            ID = mygenesforterm)
        if (!is.null(termdf)) {
            termdf <- rbind(termdf, temptermdf)
        } else {
            termdf <- temptermdf
        }



    }
    tempdf$type = ontology = types[t]
    tempdf <- merge(tempdf, termdf, by.x = "GO.ID", by.y = "term")
    if (!is.null(mergedf)) {

        mergedf <- rbind(mergedf, tempdf)
    } else {
        mergedf <- tempdf
    }

}


mergedf2 <- mergedf[mergedf$type == "BP" & mergedf$Annotated < 20, ]
selected = mergedf2$ID

merge$selected = merge$gene %in% selected
merge2 <- merge(merge, mergedf2, by.x = "gene", by.y = "ID")


p6 <- ggplot(merge[merge$selected == FALSE, ], aes(y = toxophenotype, x = Relative.Growth.Rate)) +
    geom_point(color = "lightgray", stroke = 0, size = 0.5) + annotate("rect",
    xmin = 0.4, xmax = 5, ymin = -17.5, ymax = -2, fill = "black", alpha = 0.1) +
    geom_hline(yintercept = -2, color = "black") + geom_vline(xintercept = 0.4) +
    geom_point(data = merge2, shape = 21, colour = "black", aes(fill = Term),
        size = 1.5, stroke = 0.1) + theme_bw() + scale_x_continuous(breaks = c(0,
    0.5, 1)) + scale_fill_brewer(palette = "Set1") + labs(y = "T. gondii phenotype",
    x = "P. berghei RGR", color = "P. falciparum transfections") + scale_y_continuous(breaks = c(-7.5,
    -5, -2.5, 0, 2.5)) + coord_cartesian(xlim = c(0, 1.1), ylim = c(-7.5, 2.5)) +
    theme(panel.grid.major = element_blank(), panel.grid.minor = element_blank())

p6
```

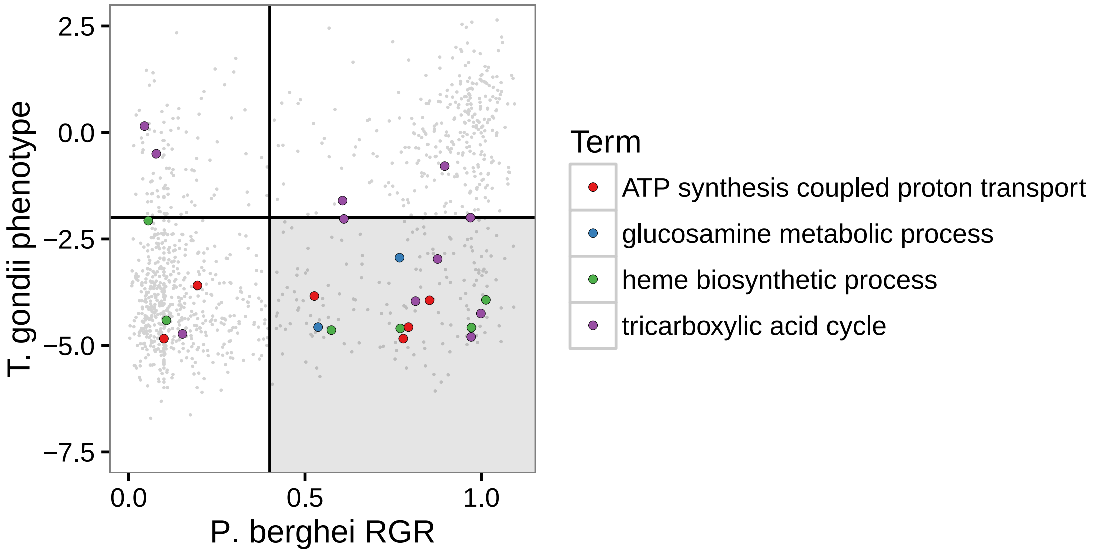


```
algorithm = "weight01"
pval = 0.05
mergedf = NULL
types <- c("BP", "MF", "CC")

interesting <- filter(merge, toxophenotype > (-2) & Relative.Growth.Rate < 0.4)$gene
for (t in 1:3) {
    gomappf <- read.csv("./otherdata/geneid2gopf.csv", stringsAsFactors = FALSE)
    gomappb <- read.csv("./otherdata/geneid2gopbnew.csv", stringsAsFactors = FALSE)

    info <- geneinfo[, c("Old.Gene.ID", "current_version_ID", "PfID")]

    gomappf <- merge(info, gomappf, by.y = "ID", by.x = "PfID")
    gomappb <- merge(info, gomappb, by.y = "ID", by.x = "current_version_ID")
    gomap <- rbind(gomappf, gomappb)[, c("Old.Gene.ID", "GO")]
    colnames(gomap) = c("ID", "GO")
    gomap <- unique(gomap)
    go <- aggregate(GO ~ ID, data = gomap, c)
    go <- go[go$ID %in% merge$gene, ]
    godb <- setNames(as.list(go$GO), go$ID)
    comb <- addPhenotypes(multicomb)
    comb <- comb[comb$phenotype != "Insufficient data", ]
    myInterestingGenes <- comb$gene
    geneList <- factor(as.integer((go$ID %in% interesting)))
    names(geneList) <- go$ID
    GOdata <- new("topGOdata", ontology = types[t], allGenes = geneList, annot = annFUN.gene2GO,
        gene2GO = godb)
    resultant <- runTest(GOdata, algorithm = algorithm, statistic = "fisher")
    allRes <- GenTable(GOdata, res = resultant, orderBy = "res", ranksOf = "res",
        topNodes = min(200, length(resultant@score)))
    tempdf <- as.data.frame(allRes)
    tempdf <- tempdf[tempdf$res < pval, ]
    myterms = tempdf$GO.ID
    mygenes <- genesInTerm(GOdata, myterms)
    termdf = NULL
```

```r
    for (i in 1:length(myterms)) {
        myterm <- myterms[i]
        mygenesforterm <- mygenes[myterm][[1]]
        temptermdf <- data.frame(term = rep(myterm, length(mygenesforterm)),
            ID = mygenesforterm)
        if (!is.null(termdf)) {
            termdf <- rbind(termdf, temptermdf)
        } else {
            termdf <- temptermdf
        }



    }
    tempdf$type = ontology = types[t]
    tempdf <- merge(tempdf, termdf, by.x = "GO.ID", by.y = "term")
    if (!is.null(mergedf)) {

        mergedf <- rbind(mergedf, tempdf)
    } else {
        mergedf <- tempdf
    }

}

mergedf2 <- mergedf[mergedf$type == "BP" & mergedf$Annotated < 20, ]
selected = mergedf2$ID

merge$selected = merge$gene %in% selected
merge2 <- merge(merge, mergedf2, by.x = "gene", by.y = "ID")

p7 <- ggplot(merge[merge$selected == FALSE, ], aes(y = toxophenotype, x = Relative.Growth.Rate)) +
    geom_point(color = "lightgray", stroke = 0, size = 0.5) + annotate("rect",
    xmin = -20, xmax = 0.4, ymin = -2, ymax = 12.5, fill = "black", alpha = 0.1) +
    geom_hline(yintercept = -2, color = "black") + geom_vline(xintercept = 0.4) +
    geom_point(data = merge2, shape = 21, colour = "black", aes(fill = Term),
        size = 1, stroke = 0.1) + theme_bw() + scale_x_continuous(breaks = c(0,
    0.5, 1)) + scale_fill_brewer(palette = "Set1") + labs(y = "T. gondii phenotype",
    x = "P. berghei RGR", color = "P. falciparum transfections") + scale_y_continuous(breaks = c(-7.5,
    -5, -2.5, 0, 2.5)) + coord_cartesian(xlim = c(0, 1.1), ylim = c(-7.5, 2.5)) +
    theme(panel.grid.major = element_blank(), panel.grid.minor = element_blank())
p7
```

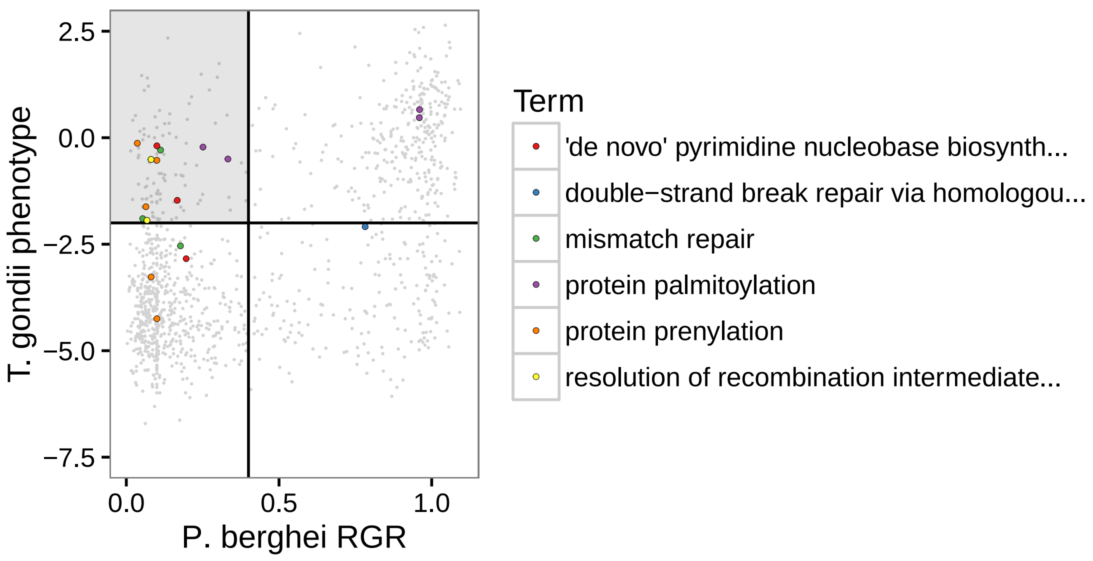

Now we will overlay data from transfection attempts in *P. falciparum* recorded in PhenoPlasm.

```
merge2 <- merge(dataset, pf, by.x = "PfID", by.y = "gene")
write.csv(merge2, "pfpb.csv")
nrow(merge2)
```

```
## [1] 133
```

```
merge2$rev = as.character(merge2$targetability)
merge2$rev[merge2$rev == "V"] = "a"

p1 <- ggplot(merge2, aes(x = Relative.Growth.Rate, fill = rev)) + geom_histogram(breaks = c(0,
    0.25, 0.5, 0.75, 1.06)) + scale_fill_manual(values = c("#007e41", "#f90f00")) +
    guides(color = FALSE) + theme_bw() + coord_cartesian(ylim = c(0, 63)) +
    scale_y_continuous(breaks = c(0, 20, 40, 60), expand = c(0, 0)) + scale_x_continuous(breaks = c(0,
    0.25, 0.5, 0.75, 1), expand = c(0, 0)) + theme(panel.grid.major.y = element_blank()) +
    guides(fill = FALSE) + labs(x = "P. berghei relative growth rate", y = "Pf frequency")

p1
```

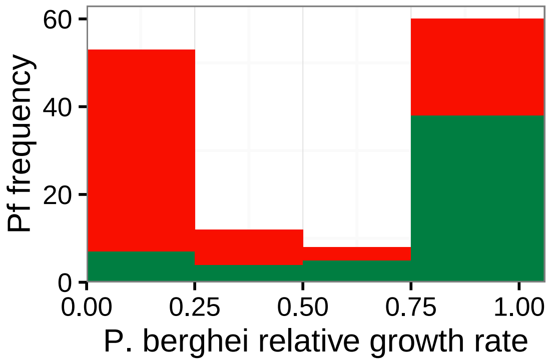


```
merge2 <- merge(toxo, pf, by.x = "PfID", by.y = "gene")
write.csv(merge2, "pftg.csv")
nrow(merge2)
```

```
## [1] 90
```

```r
merge2$rev = as.character(merge2$targetability)
merge2$rev[merge2$rev == "V"] = "a"

p2 <- ggplot(merge2, aes(x = toxophenotype, fill = rev)) + geom_histogram(breaks = c(-6,
    -4, -2, 0, 2)) + scale_x_continuous(breaks = c(-6, -4, -2, 0, 2), expand = c(0,
    0)) + scale_fill_manual(values = c("#007e41", "#f90f00")) + guides(color = FALSE) +
    theme_bw() + coord_cartesian(ylim = c(0, 31)) + scale_y_continuous(breaks = c(0,
    20, 40, 60), expand = c(0, 0)) + theme(panel.grid.major.y = element_blank()) +
    guides(fill = FALSE) + labs(x = "Toxo phenotype", y = "Pf frequency")
p2
```

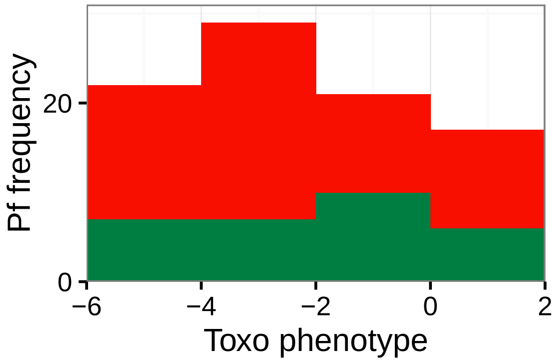


```r
p3
```

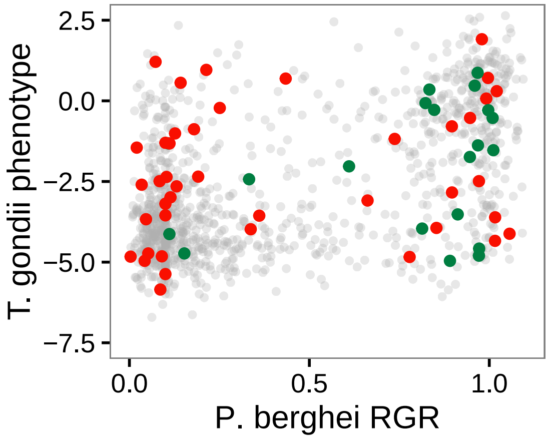

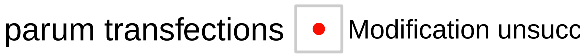

And put it all together..

```r
grid.arrange(heights = c(6, 6, 6, 3, 3, 1), p5, p4, p1, p2, legend, p8, legend6,
    p9, legend7, p11, ncol = 2, nrow = 6, layout_matrix = lay)
```

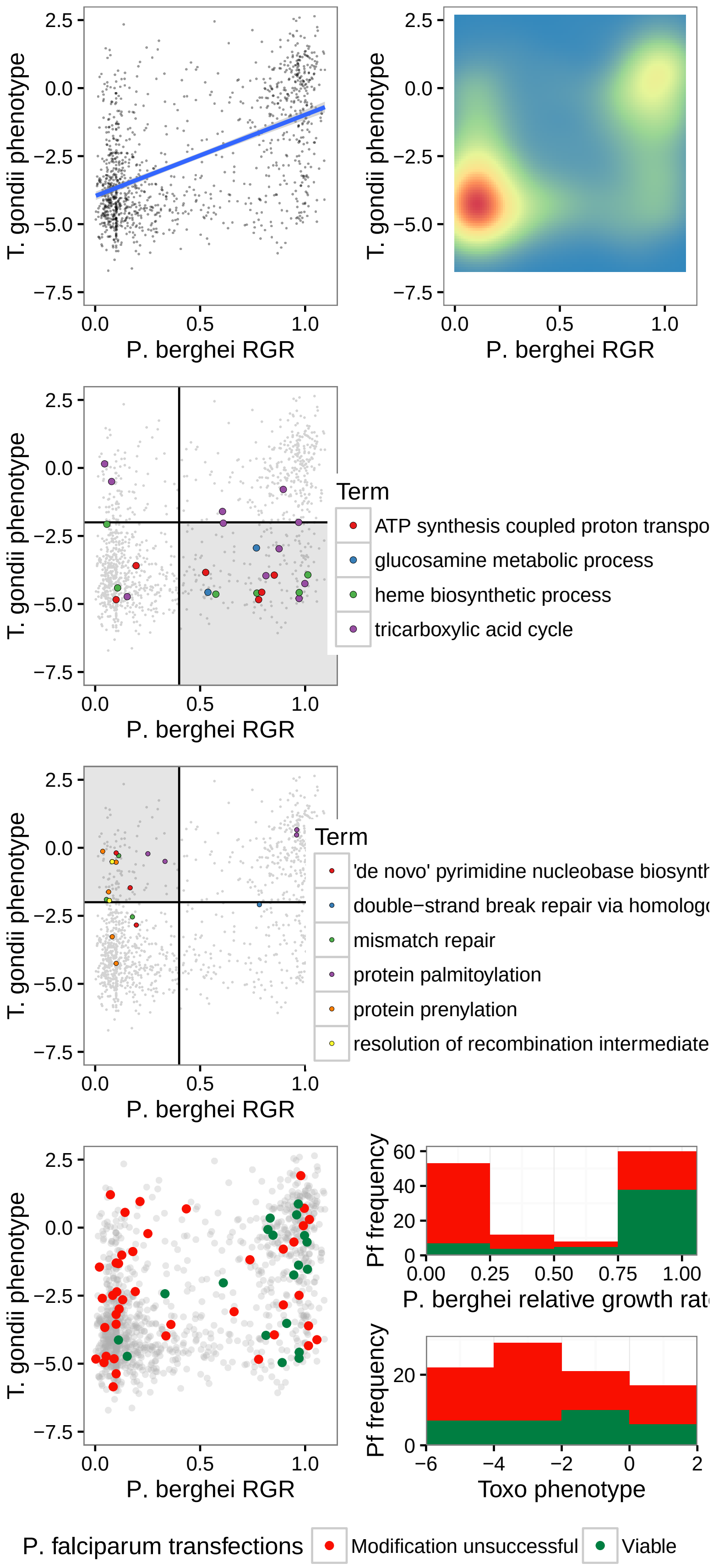

T. gondii phenotype
P. berghei RGR
Term
ATP synthesis coupled proton transpo
glucosamine metabolic process
heme biosynthetic process
tricarboxylic acid cycle
'de novo' pyrimidine nucleobase biosynth
double−strand break repair via homologo
mismatch repair
protein palmitoylation
protein prenylation
resolution of recombination intermediate
Pf frequency
P. berghei relative growth rat
Toxo phenotype
P. falciparum transfections
Modification unsuccessful
Viable
